# The mitochondria-targeted peptide SS-31 binds lipid bilayers and modulates surface electrostatics as a key component of its mechanism of action

**DOI:** 10.1074/jbc.RA119.012094

**Published:** 2020-04-09

**Authors:** Wayne Mitchell, Emily A. Ng, Jeffrey D. Tamucci, Kevin J. Boyd, Murugappan Sathappa, Adrian Coscia, Meixia Pan, Xianlin Han, Nicholas A. Eddy, Eric R. May, Hazel H. Szeto, Nathan N. Alder

**Affiliations:** ‡ Department of Molecular and Cell Biology, University of Connecticut, Storrs, Connecticut 06269; § Barshop Institute for Longevity and Aging Studies, University of Texas Health Science Center at San Antonio, San Antonio, Texas 78229; ¶ Department of Medicine, University of Texas Health Science Center at San Antonio, San Antonio, Texas 78229; ‖ Institute of Materials Science, University of Connecticut, Storrs, Connecticut 06269; ** Social Profit Network Research Lab, Alexandria LaunchLabs, New York, New York 10016

**Keywords:** membrane biophysics, mitochondria, drug action, lipid structure, peptides, cardiolipin, elamipretide, electrostatics, peptide therapeutic, SS-31, Szeto-Schiller peptide, inner membrane, bioenergetics

## Abstract

Mitochondrial dysfunction underlies many heritable diseases, acquired pathologies, and aging-related declines in health. Szeto–Schiller (SS) peptides comprise a class of amphipathic tetrapeptides that are efficacious toward a wide array of mitochondrial disorders and are believed to target mitochondrial membranes because they are enriched in the anionic phospholipid cardiolipin (CL). However, little is known regarding how SS peptides interact with or alter the physical properties of lipid bilayers. In this study, using biophysical and computational approaches, we have analyzed the interactions of the lead compound SS-31 (elamipretide) with model and mitochondrial membranes. Our results show that this polybasic peptide partitions into the membrane interfacial region with an affinity and a lipid binding density that are directly related to surface charge. We found that SS-31 binding does not destabilize lamellar bilayers even at the highest binding concentrations; however, it did cause saturable alterations in lipid packing. Most notably, SS-31 modulated the surface electrostatics of both model and mitochondrial membranes. We propose nonexclusive mechanisms by which the tuning of surface charge could underpin the mitoprotective properties of SS-31, including alteration of the distribution of ions and basic proteins at the interface, and/or modulation of bilayer physical properties. As a proof of concept, we show that SS-31 alters divalent cation (calcium) distribution within the interfacial region and reduces the energetic burden of calcium stress in mitochondria. The mechanistic details of SS-31 revealed in this study will help inform the development of future compound variants with enhanced efficacy and bioavailability.

## Introduction

Mitochondria are eukaryotic organelles that orchestrate the majority of cellular energy metabolism as well as a range of other processes, including lipid biosynthesis, ion homeostasis, and programmed cell death (apoptosis). Mitochondrial dysfunction encompasses a wide array of both genetically encoded and acquired pathologies. Several mitochondrial diseases originate from heritable mutations in genes encoding mitochondrial proteins, either in nuclear or mitochondrial DNA (1). Moreover, reduced mitochondrial function accompanies the general decline in cellular bioenergetic capacity that occurs with aging as well as with many complex pathologies, including frailty, cardiomyopathy, cancer, and neurodegeneration (2). Features of mitochondrial dysfunction include gross alterations of the inner membrane cristae morphology, destabilization of respiratory complexes of the oxidative phosphorylation (OXPHOS)^5^ system, and overproduction of reactive oxygen species (ROS) and reactive nitrogen species. Yet despite recent progress aimed at finding pharmacological approaches for mitochondrial disorders (3), there are presently no Food and Drug Administration–approved therapeutic compounds to treat them.

**Figure 1.**
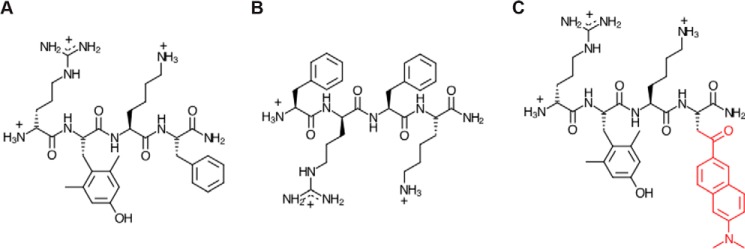
**Chemical structures of SS peptides.** *A*, SS-31 (d-Arg-2′6′-dimethyl-Tyr-Lys-Phe-NH_2_); *B*, SS-20 (Phe-d-Arg-Phe-Lys-NH_2_); *C*, [ald]SS-31, SS-31 with an aladan moiety (*red*) in place of Phe.

Szeto–Schiller (SS) peptides are among the most promising compounds currently under investigation for the treatment of mitochondrial dysfunction (4, 5). These synthetic tetrapeptides have a characteristic motif of alternating cationic and aromatic side chains (Fig. 1). Early in their development, SS peptides were shown to be cell-permeable in a wide range of cell types and to specifically target mitochondria. Despite their strong positive charge density (formal charge of +3 at neutral pH), exogenously added SS peptides traverse the plasma membrane in an energy-independent and nonsaturable manner and accumulate strongly (1000–5000-fold) at the mitochondrial inner membrane (6, 7). The intrinsic therapeutic activity of the lead compound, SS-31 (Fig. 1*A*), was first demonstrated in cell culture studies, wherein the peptides were shown to protect cells against induced oxidative stress by curtailing oxidative cell death, reducing intracellular ROS, maintaining membrane potential (ΔΨ_m_), and preventing lipid peroxidation, all in a dose-dependent manner (6, 8). SS peptides have since been shown by many independent groups using cell culture and animal disease models to have significant efficacy in restoring mitochondrial function with a wide range of pathologies, including cardiomyopathy and heart failure, skeletal muscle injury/atrophy, ischemia and ischemia-reperfusion injury, kidney injury and disease, neurodegenerative diseases, cancer, and the heritable disease Friedreich's ataxia (summarized in Ref. 5). These studies have all underscored the safety and favorable pharmacokinetic profile of SS peptides. Currently, Stealth BioTherapeutics is conducting early to advanced-phase clinical trials with SS-31 (elamipretide) for a range of primary mitochondrial and aging-related diseases.

Despite abundant evidence for the broad therapeutic potential of SS peptides, the molecular mechanism of action (MoA) of these compounds is poorly understood. It was originally proposed that SS peptides act as mitochondria-targeted antioxidants. Peptide analogs, such as SS-31, which contain a free radical-scavenging tyrosine (or dimethyltyrosine) moiety, could in principle serve to reduce ROS burden (9). However, antioxidant chemistry is not likely to be the primary mechanism of SS peptides, given (i) that such scavenging would occur stoichiometrically, not catalytically, and (ii) that other peptide variants, such as SS-20 (Fig. 1*B*), which do not contain scavenging side chains, have also proven effective in preclinical studies.

Perhaps the most critical insight into the MoA of SS peptides is that they are believed to target the mitochondrial inner membrane (MIM) by virtue of its enrichment in the anionic phospholipid cardiolipin (CL) (10–13). CL has an unusual dimeric structure with a two-phosphate headgroup and four acyl chains (Fig. S1*A*) and comprises roughly 10–20 mol % of the total lipids within the MIM. CL plays a central role in many mitochondrial processes, including apoptosis, mitophagy, signaling, fission/fusion dynamics, and energy metabolism (14, 15). Within the protein-rich MIM, CL mediates many interactions with peripheral and integral membrane proteins (16, 17), underpinning its role in the assembly of OXPHOS complexes into supercomplexes (18, 19). Moreover, with its unique physicochemical properties and propensity for conical molecular geometry and structural polymorphism, CL has a complex phase behavior in lipid bilayers (20, 21). Thus, local enrichment of CL can promote negative curvature, which likely helps to stabilize cristae architecture (22, 23). Nascent CL is synthesized *de novo* within the MIM and subsequently undergoes a remodeling process to render mature species with a highly unsaturated complement of acyl chains that is species- and tissue-specific (24) (Fig. S1*B*). Alterations in CL distribution and biogenesis are associated with a number of diseases (25), including Barth syndrome (26, 27), which is caused by defects in the transacylase tafazzin with concurrent aberrant remodeling of CL and buildup of its lysolipid form, monolysocardiolipin (MLCL). Hence, being critical for mitochondrial structure and function, the anionic phospholipids of mitochondrial membranes are promising targets for therapeutic agents.

A primary mode of interaction between SS peptides and CL-rich lipid bilayers would be a truly unparalleled type of MoA. This is because the vast majority of drug compounds are designed to target and act upon specific proteins, not the lipid bilayer (with rare exceptions, such as general anesthetics, whose mechanisms may involve alteration of bilayer properties (28)). In the present study, we analyzed the nature of the SS-31 interaction with biomimetic model membranes and intact mitochondria to understand the forces that drive the peptide-membrane interaction and how SS-31 binding affects physical properties of model and natural lipid bilayers. Our results support a molecular MoA that involves alteration of bulk lipid bilayer properties, most notably the membrane surface electrostatic profile. We discuss multiple nonexclusive mechanisms by which the regulated tuning of surface electrostatics could underpin the broad therapeutic efficacy of SS peptides.

## Results

### Binding of SS-31 to model membranes

The interaction of basic amphipathic peptides with negatively charged membranes depends on the electric field originating from the bilayer surface as well as specific electrostatic and hydrophobic interactions between the peptide and lipids (Fig. S2). The first objective of this study was to quantitatively evaluate the binding of SS-31 to model membranes with specific lipid composition. For spectroscopic analysis of SS-31 binding, we made use of the endogenous spectral properties of the 2′,6′-dimethyltyrosine (2′,6′-Dmt) side chain at the second position in the peptide. Our initial spectral characterization of SS-31 confirmed the feasibility of using endogenous peptide fluorescence to quantify peptide-membrane interactions (supporting Results A and B and Figs. S3 and S4). We therefore proceeded to measure the binding of SS-31 to large unilamellar vesicles (LUVs) containing anionic lipids in a host background of the zwitterionic lipid 1-palmitoyl-2-oleoyl-*sn*-glycero-3-phosphocholine (POPC, 16:0/18:1 PC). The anionic lipids included 1′,3′-bis[1,2-dioleoyl-*sn*-glycero-3-phospho]-glycerol (tetraoleoylcardiolipin (TOCL), all acyl chains 18:1), monolysocardiolipin (MLCL, all acyl chains 18:1), or 1-palmitoyl-2-oleoyl-*sn*-glycero-3-phosphoglycerol (POPG, 16:0/18:1 PG). Binding measurements were performed using two complementary approaches: (i) titration of fixed concentrations of LUVs with peptide and (ii) titration of fixed concentrations of peptide with LUVs (supporting Results C and Fig. S5). Note that in our analyses, we assume that peptide does not cross the bilayer of our model systems and can only access the outer leaflet; for LUVs of this size, the “effective lipid concentration” of the outer leaflet (herein referenced as [L]^eff^, [Lip]^eff^, or [lipid]^eff^) is taken as half of the total lipid concentration in the system (29).

**Figure 2.**
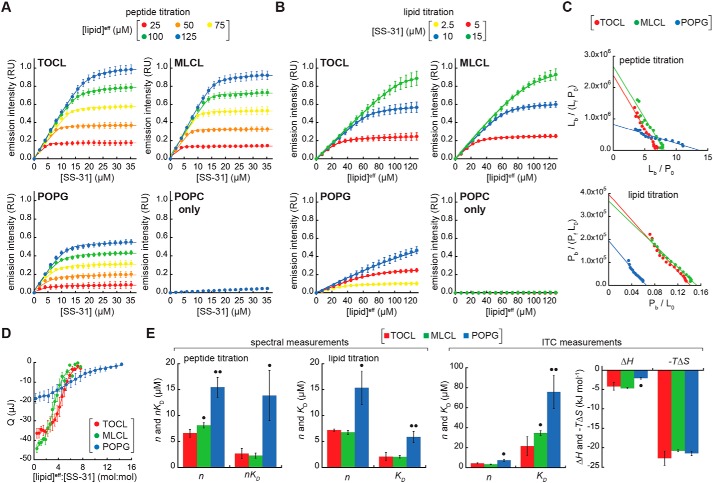
**SS-31 binding isotherms.** *A–C*, fluorescence-based measurements. *A*, peptide titrations. Liposomes containing anionic lipid (20 mol % TOCL, MLCL, or POPG in a POPC background) or POPC only at the lipid concentrations indicated were titrated with SS-31 (4-nmol increments). Values shown are means ± S.D. (*error bars*) (*n* = 3), and *lines* show data fits based on Equation S2. *B*, lipid titrations. Peptides at the concentrations indicated were titrated with liposomes (30-nmol total lipid increments). Values shown are means ± S.D. (*error bars*) (*n* = 3), and *lines* show data fits based on Equation S3. *C*, Scatchard analyses. Titration data with liposomes containing 20 mol % TOCL, MLCL, and POPG are shown as Scatchard plots. *Top*, peptide titrations ([lipid]^eff^ = 125 μm) fit according to Equation S6; *bottom*, lipid titrations (10 μm SS-31, TOCL, and MLCL; 5 μm SS-31, POPG) fit according to Equation S8. *D*, ITC analyses. A Wiseman plot shows blank-subtracted average integrated heats (means ± S.D. (*error bars*); *n* = 3) as a function of [lipid]^eff^/[SS-31] molar ratio for LUVs containing TOCL, MLCL, and POPG. *E*, summary of binding parameter data. Statistical differences within each data set were determined relative to TOCL-containing membranes: •, *p* < 0.05; ••, *p* < 0.01.

Binding isotherms based on SS-31 emission intensity are shown in Fig. 2. First, peptide titrations were conducted by the progressive addition of SS-31 to LUVs of different composition at lipid concentrations ranging from 25 to 125 μm (Fig. 2*A*; see also supporting Results D and Figs. S6 and S7). Second, lipid titrations were conducted by the progressive addition of LUVs to SS-31 at concentrations up to 15 μm (Fig. 2*B*). Taken together, these binding curves reveal the following: (i) in the absence of anionic lipid (LUVs with POPC only), SS-31 binding is negligible even at the highest lipid concentrations used, consistent with the established requirement of anionic lipids for binding (10–13); (ii) in the presence of anionic membranes, SS-31 binding displays saturation binding behavior; and (iii) membranes containing dianionic lipid (TOCL and MLCL) have a higher SS-31 binding capacity than membranes containing monoanionic POPG, reflected in the roughly 2-fold higher [SS-31] required to saturate binding in peptide titrations and in the roughly 2-fold lower [lipid] required to saturate binding in lipid titrations.

To obtain equilibrium binding parameters *n* (the number of lipid molecules per peptide bound) and *K_D_* (the dissociation constant), we analyzed binding isotherm data by Scatchard analysis (Fig. 2*C*) and by fits of the data to Langmuir adsorption models (Equations S2 and S3 for peptide and lipid titrations, respectively). The equilibrium binding parameters from this work (Fig. 2*E*, *left panels*) reveal two key points. First, for membranes containing cardiolipin variants, the average lipid/peptide stoichiometry (*n* = 6.9 and 7.4 for TOCL and MLCL, respectively) is roughly half the value for membranes with POPG (*n* = 15.4). Hence, if we assume ideal lipid mixing and approximate lipid cross-sectional areas (70 Å^2^ for POPC and POPG, 130 Å^2^ for TOCL, and 110 Å^2^ for MLCL (30, 31)), then for membranes composed of 20% TOCL or MLCL, the SS-31 “binding site” comprises an area of ∼560–580 Å^2^ in which each peptide associates, on average, with 1.4–1.5 dianionic lipids. By comparison, for membranes containing 20% POPG, SS-31 binds an area of roughly 1080 Å^2^ and associates on average with three monoanionic lipids. Based on these stoichiometric relationships, then, there is an approximate charge balance between each peptide and its corresponding lipid binding site. Second, the affinity of SS-31 for membranes containing TOCL and MLCL is significantly higher than it is for membranes containing POPG. Specifically, the average dissociation constants for lipid monomers (reflected in *K_D_* values for lipid titrations and in *nK_D_* values for peptide titrations) are 2.0–2.9 μm for membranes composed of 20% TOCL and MLCL, whereas they range from 6.0 to 13 μm for membranes composed of 20% POPG. These values of *n* and *K_D_* are consistent with parameters measured for other amphipathic membrane-active peptides (32, 33). They are also consistent with previous studies showing a strong correlation between binding energies of basic peptides and the negative surface charge of lipid bilayers (*e.g.* see Ref. 34). As an independent measure of peptide binding, the molar partition coefficients (*K_P_*, Equation S9) for membranes containing 20% TOCL (*K_P_* = 5.07 ± 0.76 × 10^4^) and 20% MLCL (*K_P_* = 5.90 ± 0.44 × 10^4^) are significantly higher than that of 20% POPG-containing membranes (*K_P_* = 1.28 ± 0.63 × 10^4^).

As a complementary approach for measuring SS-31 membrane interactions, we performed isothermal titration calorimetry (ITC) measurements by the progressive injection of LUVs into solutions of peptide in the sample cell (35). The interaction of SS-31 with membranes containing anionic lipid was shown to be exothermic in all cases (Fig. S8), and fits of complete binding isotherms (Fig. 2*D*) yielded the thermodynamic binding parameters shown (Fig. 2*E*, *right panels*). The values of *n* and *K_D_* from fits to the ITC data showed consistent differences between TOCL/MLCL- and POPG-containing membranes when compared with our spectral-based measurements (Fig. 2). However, compared with our spectral analyses, there were differences in the absolute values from ITC-derived parameters (mean *n* values slightly lower and *K_D_* values ∼2–3 times higher). This difference could be related to different phenomena being measured: our spectral analyses may only reflect the binding event *per se*, whereas ITC measurements could be reflecting binding as well as peptide-dependent alterations of bilayer properties. Apropos of this point, we did observe a slight asymmetry in our exothermic peaks, particularly for TOCL- and MLCL-containing membranes, which could be related to thermotropic alterations in the bilayers following peptide interaction.

Comparison of the binding-associated changes in enthalpy (Δ*H*) and entropy (Δ*S*) provides insights into the membrane-binding mechanism. Namely, exothermic (Δ*H* < 0) values of peptide-lipid interaction, as we observe for SS-31, originate mainly from the establishment of polar contacts; by contrast, increases in the system entropy (Δ*S* > 0), as we also observe for SS-31, originate mainly from the burial of hydrophobic side chains in the acyl chain region with concurrent release of water and ions from nonpolar surfaces of the peptide and bilayer (36). For membranes containing TOCL, MLCL, and POPG, the interaction of SS-31 was associated with a favorable enthalpic change (Δ*H* = −4.2, −4.7, and −2.1 kJ mol^−1^, respectively); the 2-fold difference in Δ*H* between TOCL/MLCL- and POPG-containing membranes is consistent with the difference in formal charges of these anionic lipids. The peptide-bilayer association, however, was predominantly entropy-driven (−*T*Δ*S* = −22.7, −20.8, and −21.5 kJ mol^−1^, respectively), showing that peptide association is stabilized mostly by burial of the nonpolar residues. Indeed, earlier studies on the interaction of small basic peptides with anionic lipid bilayers indicated a dominant role of entropy over enthalpy in stabilizing the interaction (*e.g.* see Ref. 37). Given that the hydrophobic contribution to peptide binding energy is proportional to the exposed side-chain area (∼80 J mol^−1^ Å^−2^) (38), the burial of Tyr (similar to 2′,6′-Dmt) and Phe side chains (accessible surface areas 187 and 175 Å^2^, respectively) would theoretically contribute a combined ∼28 kJ mol^−1^, in reasonable agreement with our measurements. Moreover, because SS peptides are short and probably do not form any appreciable secondary structure upon membrane binding, their membrane binding does not likely incur an entropic penalty that would be associated with stabilizing a secondary structure.

Taken together, our spectral and calorimetric binding data show that the bilayer binding density and affinity of SS-31 are directly related to the anionic lipid composition, that the lipid-dependent differences in interaction energy are mostly on the net charge of anionic lipids, and that membrane interactions are enthalpically and entropically favorable.

### Dependence of SS-31 binding on model membrane surface electrostatics

**Figure 3.**
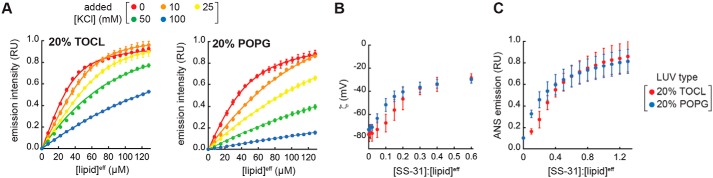
**SS-31 binding and surface electrostatics.** *A*, binding isotherms. Peptide was titrated with LUVs of the indicated composition (20% TOCL, [SS-31] = 7.5 μm and 20% POPG, [SS-31] = 3.8 μm) at 30-nmol total lipid increments in the presence of different concentrations of added KCl. Values shown are means ± S.D. (*error bars*) (*n* = 3), and *lines* show data fits based on Equation S3. Because isotherms at higher [KCl] did not reach saturation, curves within each membrane set were fit globally, assuming a common saturation point. *B* and *C*, effect of SS-31 binding on surface charge. Measurements of ζ (*B*) and ANS fluorescence (*C*) are shown as a function of [SS-31]/[lipid]^eff^ for membranes containing 20% TOCL and 20% POPG as indicated. Values shown are means ± S.D. (*error bars*) (*n* = 3).

The electrostatic profile of biomembranes consists of multiple potential energy functions (Fig. S2). Among them, the surface potential (ψ_s_) originates from charge distribution at the membrane interface based on ionizable functional groups (*e.g.* lipid headgroups) and adsorbed ions, creating a strong electrostatic driving force for the binding of charged moieties to the bilayer surface (39). Electrolytes partitioned at the bilayer interface can have a complex effect on membrane interactions of amphipathic peptides. By attenuating the ψ_s_, solution cations can reduce the electrostatic attraction of basic peptides to the anionic lipid surface; however, increasing solution ionic strength may also favor the burial of hydrophobic side chains in the nonpolar core of the bilayer. We therefore explored the relationship between SS-31 binding and the ψ_s_ of model membranes and the influence of ionic strength (Fig. 3).

SS-31 binding curves measured by titration of SS-31 with LUVs containing 20% TOCL or POPG revealed a strong effect of monovalent electrolyte (Fig. 3*A*). We confirmed that SS-31 binding to model bilayers was reversible in a manner that depends upon solution ionic strength used for these binding isotherms (supporting Results E and Fig. S9 (*A* and *B*)). When binding parameters *n* and *K_D_* calculated from the curves of Fig. 3*A* are considered as a function of salt concentration, there is a large disparity between membranes containing divalent TOCL and monovalent POPG (Fig. S9*C*, *top panels*). However, recasting these parameters as a function of ψ_s_, calculated from Gouy-Chapman-Stern (GCS) formalism (40, 41) (Equations S10–S12), reveals a much smaller difference between membranes containing the two anionic lipids (Fig. S9*C*, *bottom panels*). This GCS analysis, which accounts for the roughly 2-fold larger intrinsic surface charge (σ^max^) of TOCL- *versus* POPG-containing bilayers, indicates that SS-31 binding is more related to the prevailing surface charge (σ) *per se*, rather than to any specific features of CL.

To continue this analysis, we determined the relationship between SS-31 binding and surface electrostatics by two complementary approaches. We first performed zeta potential (ζ) measurements of LUVs containing 20 mol % TOCL or POPG with increasing [SS-31] (Fig. 3*B*). The ζ represents the electrostatic potential at the hydrodynamic shear plane of membranes and is related to the ψ_s_ by the Debye constant (κ), which describes the position-dependent decay of electrostatic potential from the membrane surface to the bulk solution. As expected for the binding of a polybasic peptide to a negatively charged surface, SS-31 caused a saturable reduction in the magnitude of ζ for both anionic bilayers. As an independent technique, we measured the effect of SS-31 on surface electrostatics using 1-anilinonaphthalene-8-sulfonic acid (ANS), a fluorescent reporter whose binding to anionic bilayers increases as the magnitude of the ψ_s_ is reduced, measured as an increase in probe emission intensity (42) (Fig. 3*C* and Fig. S9*D*). Consistent with our ζ data, SS-31 caused a saturable ANS-detected reduction in the ψ_s_ of anionic model membranes. We observed the same response with SS-20 (Fig. S9*E*), supporting that this effect on membrane surface electrostatics is a general feature of SS peptides. Beyond the observation that SS peptides attenuate membrane surface potential, two features are notable from these experiments. First, for both ζ and ANS measurements, SS-31 elicited a hyperbolic decay of ψ_s_ for PG-containing bilayers, whereas the response was more sigmoidal for CL-containing bilayers (Fig. 3 (*B* and *C*) and Fig. S9 (*D* and *E*), *blue versus red*). This suggests that at low peptide concentration, there is not a linear correspondence between peptide binding and charge attenuation for CL-containing membranes. Second, the ζ responses, which provide an absolute measure of ψ_s_, consistently saturated near −30 mV with increasing peptide. This indicates that when the model bilayers are maximally bound with peptide, there remains an appreciable negative surface charge density. This observation is in contrast to some other basic peptides (43) and multivalent cations (44) that cause charge overcompensation (lead to positive surface charge density) upon binding anionic bilayers at high concentration. Two nonexclusive explanations may account for this: (i) at binding saturation, bound SS-31 may render some ionized phosphate groups unavailable for further interaction, and/or (ii) the formal charge on SS-31 may become reduced upon binding. Regardless of the mechanism, the fact that SS-31 attenuates the ψ_s_, but does not completely reverse it, is a fundamental feature of its effect on the membrane surface electrostatic profile.

### SS-31 maintains the lamellarity of model membranes but alters lipid interactions at the interface

**Figure 4.**
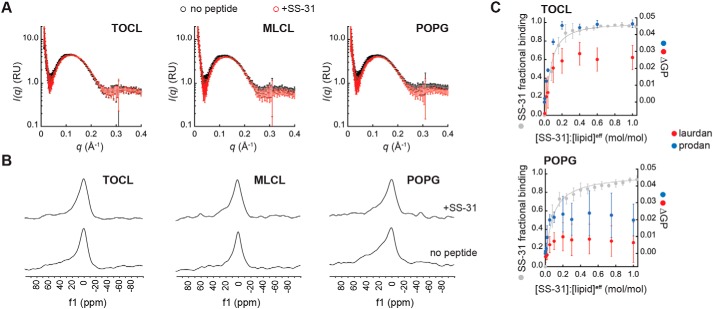
**Effects of SS-31 on structural properties of model membranes.** *A* and *B*, SS-31 does not cause major structural perturbation of model membranes. LUVs composed of 20% anionic lipid (TOCL, MLCL, or POPG) in POPC background were measured in the absence or presence of peptide ([SS-31]/[lipid]^eff^ = 1:5) as indicated. *A*, synchrotron SAXS measurements showing background-subtracted scattering density profiles for LUVs in the absence (*black*) or presence (*red*) of SS-31. *B*, ^31^P solid-state NMR spectra of LUVs in the absence (*bottom spectra*) or presence (*top spectra*) of SS-31. *C*, SS-31 alters lipid packing interactions. Shown are changes in laurdan and prodan GP values (ΔGP) with increasing [SS-31] for LUVs containing 20% TOCL or POPG in POPC background as indicated. Data and fits in *gray* show SS-31 fractional saturation (calculated from Fig. 2*A*, [lipid]^eff^ = 25 μm). Values shown are means ± S.D. (*error bars*) (*n* = 3).

Amphipathic molecules such as antimicrobial peptides can induce large structural changes in membranes, including micellization, pore formation, and induction of inverted topologies (45). We therefore addressed whether SS-31 induced structural polymorphism within model bilayers using a range of approaches (Fig. 4). Based on synchrotron small-angle X-ray scattering (SAXS) measurements, LUVs composed of POPC alone or with 20% TOCL, MLCL, or POPG yielded scattering profiles typical of lipid vesicles (46), and the presence of SS-31 did not cause structural perturbation (Fig. 4*A*, supporting Results F, and Fig. S10*A*). Similarly, based on ^31^P solid-state NMR (ssNMR) measurements, these model membranes yielded line shapes consistent with lamellar bilayers (47), and we observed no effect of SS-31 on bilayer structure (Fig. 4*B*, supporting Results F, and Fig. S10*B*). We conclude that even at the highest peptide concentration used in this study ([P]/[L]^eff^ = 1:5), SS-31 did not cause major topological alterations of our model membranes. Thus, these membranes are expected to exist stably in the liquid crystalline lamellar mesophase over all peptide concentrations.

We therefore addressed whether SS-31 altered lipid interactions within these model bilayers. To this end, we used membrane-bound fluorescent reporters of lipid dynamics and packing that partition into the bilayer at different depths (Fig. S10*C*). Using 1,6-diphenyl-1,3,5-hexatriene (DPH) anisotropy (〈*r*〉^DPH^) as a readout of the fluid dynamics of hydrocarbon tails, we observed no effect of SS-31 in TOCL- or POPG-containing membranes (Fig. S10, *D* and *E*). We then used the solvatochromic probes 6-dodecanoyl-2-dimethylaminonaphthalene (laurdan) and 6-propinoyl-2-dimethylaminonaphthalene (prodan), whose spectral properties are quantified as the generalized polarization (GP^LAU^ and GP^PRO^, respectively) that increases with enhanced lipid packing (reduced interfacial hydration) (48) (see supporting Results F and Fig. S10F). We observed modest, yet repeatable and saturable, increases in GP^LAU^ and GP^PRO^ that corresponded to the fractional occupancy of peptide on the membrane (Fig. 4*C*). Notably, the response of GP values in TOCL-containing bilayers was much higher in magnitude compared with the response in those with POPG. Based on these results, SS-31 binding causes a change in the hydration/polarity of the bilayer interface that likely correlates with an increase in lipid headgroup packing density.

### Molecular dynamics analysis of the interaction between SS-31 and lipid bilayers

**Figure 5.**
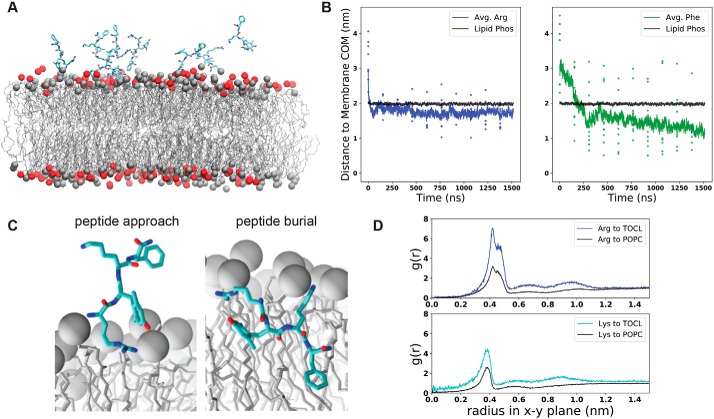
**Molecular dynamics simulations of SS-31 bilayer interactions.** *A*, snapshot of a typical MD simulation with SS-31 peptides in the aqueous phase relative to the upper leaflet of a 20% TOCL bilayer. Peptides are shown in *licorice representation* (*cyan*, carbon; *blue*, nitrogen; *red*, oxygen), lipid acyl chains are in *wireframe*, and lipid phosphates are shown as van der Waals *spheres* for POPC (*gray*) and TOCL (*red*). *B*, temporal evolution of SS-31 Arg (*blue*) and Phe (*green*) side-chain positions relative to the membrane center of mass (*COM*). *Black lines* show the average position of all phosphates in the upper leaflet, *colored dots* show *z*-coordinates (*z*^POS^) of individual side chains, and *colored lines* represent average *z*^POS^ values. *C*, orientation of SS-31 bound to a lipid bilayer consisting of 20 mol % TOCL in a POPC background in the peptide approach (*left*) and buried (*right*) states. Peptide representation is as in *A*, with all lipid phosphates shown as *gray* van der Waals *spheres. D*, lipid distributions around basic SS-31 residues. Shown are lateral (*x-y* plane) radial distribution profiles from Arg and Lys side chains to lipid headgroup phosphate atoms of TOCL or POPC as indicated.

We next used all-atom molecular dynamics (MD) simulations to analyze the interaction between SS-31 and lipid bilayers (Fig. 5, supporting Results G, and Figs. S11–S14). These simulation systems contained solvated bilayers composed of 20 mol % TOCL, MLCL, or POPG in a host background of POPC and were conducted in the presence or absence of SS-31 to evaluate SS-31–bilayer interactions and how the presence of SS-31 may affect bilayer properties (Fig. 5*A*). In our initial investigations, we performed spontaneous membrane binding simulations by placing 10 SS-31 peptides in the aqueous phase in random orientations at distances of 1–3 nm from the bilayer surface and conducting simulations for up to 1.6 μs. Throughout each trajectory, we quantified the distance between each side chain and the bilayer center of mass along an axis perpendicular to the membrane (the *z*-coordinate, Fig. S11 and Fig. 5*B*). We observed rapid association (<200 ns) of all peptides to the bilayer surface, in which all side chains had docked to the membrane near the interfacial region, presumably driven by the electrostatic attraction between the polybasic SS-31 and the negative surface charge of the lipid bilayer. However, the membrane association time differed among the side chains. For example, the N-terminal Arg adsorbed to the bilayer rapidly (within 50 ns), whereas the C-terminal Phe took significantly longer to localize to the bilayer (within 180 ns) (Fig. 5*B*).

From these simulations, we observed two main types of SS-31 poses on the membrane (Fig. 5*C*): a “peptide approach” state, in which the primary points of contact with the membrane are the N terminus and Arg side chain anchored to phosphate headgroups, and a “peptide burial” state, in which the aromatic side chains have partitioned into the nonpolar core while the basic side chains remain electrostatically tethered to headgroup phosphates. Notably, the burial of the aromatic side chains of SS-31 in the acyl chain region is consistent with previous NMR studies (10). Once the peptides reached the burial state, each side chain assumed a distinct, stable membrane insertion depth, quantified as the average *z*-coordinate between the side chain and the membrane center of mass (*z*^pos^) (Fig. S11, *B* and *C*). In the 20:80 TOCL/POPC system, the basic side chains occupied the interfacial region, with Arg (*z*^pos Arg^ = 1.75 nm) residing just below the average position of lipid phosphates (*z*^pos Phos^ = 1.98 nm) and Lys residing at a slightly more distal position near the headgroups (*z*^pos Lys^ = 1.99 nm). By comparison, the aromatic residues assumed stable positions within the nonpolar core with the Phe side chain burying deeper (*z*^pos Phe^ = 1.12 nm) than the 2′,6′-Dmt side chain (*z*^pos Dmt^ = 1.37 nm). These residue-specific membrane insertion depths were consistent across the three different anionic lipid compositions tested (20% TOCL, 20% MLCL, and 20% POPG) (Fig. S11*C*).

To evaluate the association of SS-31 side chains with lipid headgroups, we analyzed radial distribution profiles from our MD trajectories (Fig. 5*D* and Fig. S12*A*). This analysis revealed that basic side chains of SS-31 preferentially interacted with anionic lipids. For example, based on *g*(*r*) peak height, in lipid systems with 20% TOCL, Arg and Lys showed 2.25- and 1.72-fold relative increases in local concentration of phosphate groups from TOCL compared with those from POPC (Fig. 5*D*). These results are consistent with peptide-lipid co-diffusion, suggesting that the SS-31 Arg can concurrently associate with multiple lipid phosphates with a preference for TOCL over POPC (Fig. S12*B*). As a complement to these analyses, we evaluated how the presence of SS-31 may influence lipid bilayer properties. First, lipid-to-lipid radial distribution profiles showed a slight peptide-dependent local clustering of anionic lipids (Fig. S12*C*). Second, based on the lateral mean square displacement of lipid phosphates, we observed marked peptide-dependent decreases in the lateral diffusivity (*D_xy_*) of all tested lipids (Fig. S13). Finally, to study how SS-31 binding could affect membrane packing defects, we measured the solvent-accessible surface area of acyl chains in systems with and without SS-31 (Fig. S14). This analysis showed peptide-dependent decreases in acyl chain solvent accessibility of all tested lipids, consistent with our observed effects of SS-31 on interfacial hydration and packing density (Fig. 4*C*). Hence, taken together, our MD simulations elucidate the nature of polar and nonpolar interactions that mediate the SS-31-lipid bilayer interaction and how peptide binding may affect lipid distribution, reduce lateral lipid mobility, and decrease the accessibility of solvent (and solvated ions) to acyl chains that may otherwise be exposed to the interface by packing defects.

### SS peptide interactions with mitochondrial membranes

Having evaluated the binding of SS-31 with model membranes, the next objective was to quantitatively assess how this peptide interacts with mitochondria. We first evaluated peptide binding interactions. The spectral complexity of mitochondria precludes analysis of SS-31 binding by endogenous peptide fluorescence, so we used instead the variant [ald]SS-31, which contains the environment-sensitive fluorophore aladan (49) in place of the C-terminal Phe residue (Fig. 1*C*). As shown previously, [ald]SS-31 displays a blue-shifted emission spectrum and an increase in fluorescence intensity when bound to bilayers, making it an excellent reporter for membrane interaction. This peptide variant has also been shown to target mitochondria in a manner similar to SS-31 (11, 12). We first thoroughly evaluated the binding of [ald]SS-31 to model membranes composed of POPC with TOCL, MLCL, and POPG in different molar ratios and under different ionic conditions (Fig. S15, *A* and *B*). Equilibrium binding parameters for [ald]SS-31 with model membranes compared favorably with those of SS-31 (Fig. S15*C*). Hence, this variant served as a suitable model for SS-31 binding to mitochondria.

**Figure 6.**
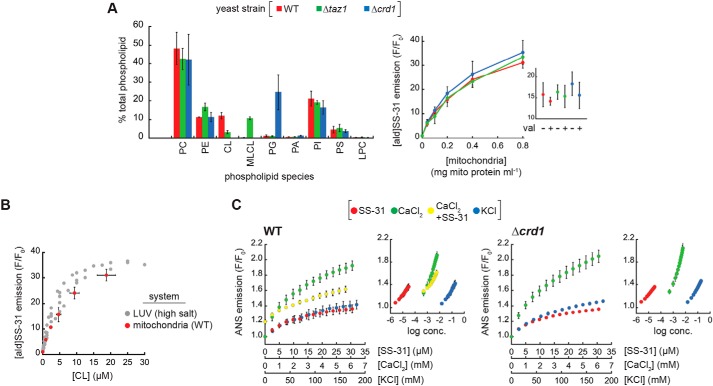
**Interaction of SS-31 with isolated mitochondria.** *A*, [ald]SS-31 binding with differing phospholipid composition. *Left*, percentages of total phospholipid of each species (phosphatidylcholine (*PC*), phosphatidylethanolamine (*PE*), cardiolipin (*CL*), monolyso-CL (*MLCL*), phosphatidylglycerol (*PG*), phosphatidic acid (*PA*), phosphatidylinositol (*PI*), phosphatidylserine (*PS*), and lysophosphatidylcholine (*LPC*)) in mitochondria from WT, Δ*taz1*, and Δ*crd1* strains measured by shotgun lipidomics. *Right*, binding of 1 μm [ald]SS-31 with mitochondria isolated from WT, Δ*taz1*, and Δ*crd1* yeast strains quantified as the mean fractional increase (*F*/*F*_0_) in aladan emission with increasing mitochondria concentration for fully energized mitochondria. *Inset*, relative binding of [ald]SS-31 to mitochondria (0.2 mg ml^−1^) in the presence or absence of 1 μm valinomycin (*val*) as indicated. Values shown are means ± S.D. (*error bars*) (*n* = 3). *B*, comparison of [ald]SS-31 binding to mitochondria and model membranes. [ald]SS-31 binding is shown as a function of CL concentration for (i) LUVs of defined TOCL composition under high-salt conditions (*gray*; Fig. S15) and (ii) mitochondria isolated from yeast WT strain (*red*; Fig. S16 and Fig. 6*A*). Calculation of [CL] for LUVs accounts for outer leaflet (accessible) CL only from known lipid concentrations; calculation of [CL] for mitochondrial samples was based on lipidomics analyses of Fig. 6*A*, accounting for total CL content. *C*, effects of SS-31 and salt cations on mitochondrial surface potential. Fractional change in ANS emission is shown for titration of mitoplasts from WT (*left*) and Δ*crd1* (*right*) yeast with SS-31, CaCl_2_, or KCl at the indicated concentrations. For WT samples, CaCl_2_ titrations were also conducted following the addition of 20 μm SS-31 (*CaCl_2_* + *SS-31*). *Insets*, ANS emission data plotted as a function of log concentration for all three titrants. Values shown are means ± S.D. (*error bars*) (*n* = 3).

In our evaluation of SS-31 mitochondrial interactions, our first goal was to assess the CL dependence of peptide binding. Because of the relative simplicity of the CL biosynthesis and remodeling pathway in yeast compared with higher eukaryotes (Fig. S1*B*), *Saccharomyces cerevisiae* is an excellent model organism to directly compare effects of altered CL metabolism among otherwise isogenic systems. We therefore used mitochondria isolated from *S. cerevisiae* strains with normal CL metabolism or with knockouts in cardiolipin synthase (Δ*crd1*) or the transacylase tafazzin (Δ*taz1*) (50). Shotgun lipidomics data confirmed the expected phospholipidome of mitochondria from these three strains (Fig. 6*A*, *left*), namely that Δ*crd1* mitochondria lack CL and have a buildup of PG, the immediate biosynthetic precursor of CL and that Δ*taz1* mitochondria show an accumulation of MLCL and reduced CL. Further, the acyl chain distribution of anionic lipids among these strains shows the expected patterns of fatty acid saturation (50) (Fig. S16*A*). Emission scans of [ald]SS-31 revealed spectral changes with increasing mitochondria consistent with membrane interaction (Fig. S16*B* and Fig. 6*A*, *right*), showing two important features. First, as shown previously (8), collapse of the transmembrane potential (ΔΨ_m_) by the ionophore valinomycin had no effect on peptide interaction (Fig. 6*A*, *inset*). Second, the relative binding of [ald]SS-31 was nearly identical among the WT, Δ*crd1*, and Δ*taz1* strains. Similar peptide binding between WT and Δ*taz1* strains is expected, given that tetraacyl TOCL and triacyl MLCL equally support SS-31 binding in our reductionist systems (Figs. 2 and 3). Uninhibited peptide binding by the Δ*crd1* strain is perhaps surprising given that POPG supports significantly less peptide binding than CL (Figs. 2 and 3). However, our lipidomics data indicate that PG levels are dramatically increased when CL synthesis is blocked. This increase in PG likely creates a negative surface charge density of the MIM large enough to promote peptide binding at WT levels. Again, this supports a model in which SS-31 interaction is related more to surface charge than it is to specific lipid headgroup identity.

The second goal of these binding experiments was to directly compare peptide binding between model membranes and mitochondria. We reasoned that the best comparison would come from quantifying [ald]SS-31 binding as a function of a common independent variable (CL composition), made possible using the known mitochondrial CL concentration from our lipidomics data. When evaluated in this way, the binding curves of [ald]SS-31 to LUVs and WT mitochondria are strikingly similar (Fig. 6*B*). This comparison, although indirect, is consistent with a model in which binding of SS-31 to mitochondria is governed by the same interactions in our model systems (*i.e.* through lipid bilayer interactions).

The final goal of these experiments was to evaluate the effect of SS peptides on the surface electrostatic properties of mitochondrial membranes. Based on our observation that SS peptides attenuated the ψ_s_ of model membranes (Fig. 3 and Fig. S9), we again used the ANS fluorescent reporter to measure the ψ_s_ of mitoplasts (mitochondria with osmotically ruptured outer membrane to allow probe access to the MIM). We therefore compared the titration of mitoplasts with SS-31 and mono- and divalent cations (K^+^ and Ca^2+^, respectively) to evaluate the relative effects of each species on ANS-detected ψ_s_ (Fig. 6*C*). All three cationic species caused an increase in ANS fluorescence, reflecting a decrease in the ψ_s_ of mitochondrial membranes from WT (*left*) and Δ*crd1* (*right*) yeast. Consistent with the exponential dependence of formal charge on accumulation of ionized species in the electric field of membranes (Equation S11), the effective concentration ranges of SS-31, Ca^2+^, and K^+^ progressively differed by about 2 orders of magnitude (Fig. 6*C*, *insets*). The first notable feature of this analysis is the relative effect of each species on surface potential. Compared with K^+^, Ca^2+^ ions caused a much stronger impact on the relative ψ_s_, as expected. Interestingly, however, within its effective concentration range, SS-31 had a reduced effect on the relative ψ_s_ that was closer to that of the monovalent cation, an observation consistent with the fact that SS-31 does not completely reverse negative surface potential of model membranes even upon binding saturation (Fig. 3*C*). The second notable feature of this analysis is the effect of SS-31 on Ca^2+^-mediated decreases in the relative ψ_s_. SS-31 addition to WT mitoplasts prior to CaCl_2_ titration strongly blunted the effects of Ca^2+^ on surface potential (Fig. 6*C*, *left*, compare *green* and *yellow symbols*), supporting a binding model in which SS-31 preferentially binds to mitochondrial membranes over Ca^2+^ ions. Taken together, we conclude from the results of Fig. 6 that the binding of SS peptides to mitochondrial membranes is strongly dependent on surface charge of the lipid bilayer, that SS peptide binding causes a “controlled down-tuning” of the electric field originating from the membrane surface, and that SS peptides at the membrane interface can mitigate the effects of polyvalent metal cations on membrane surface electrostatics.

### The effects of SS peptides on interfacial cation accumulation

**Figure 7.**
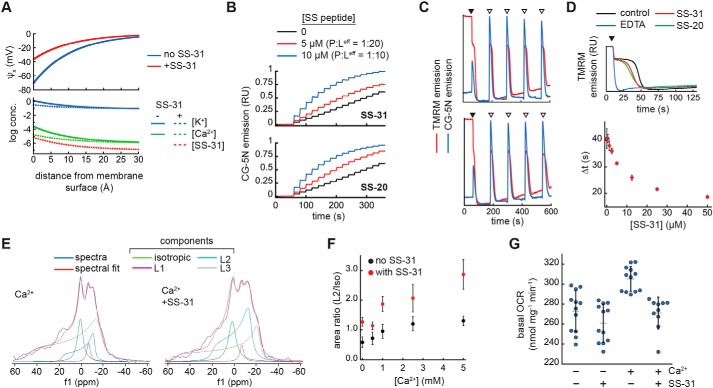
**SS peptide effects on Ca^2+^-membrane interactions and mitochondrial cation stress.** *A*, profiles of surface potential decay (*top*) and ion distribution (*bottom*) based on GCS theory in the presence or absence of SS-31 (see supporting Results H for model description). *B*, effect of SS peptides on Ca^2+^ accumulation at model membrane interfaces. Time course measurements of CG-5N emission were performed in solutions with LUVs (20 mol % TOCL in host POPC background, [lipid]^eff^ = 100 μm) prebound with SS peptide at the indicated concentration followed by titration of CaCl_2_ in increments of 1 nmol at 20-s intervals starting at *t* = 60 s. *C* and *D*, effect of SS peptides on mitochondrial Ca^2+^ uptake. *C*, time courses of TMRM or CG-5N emission in mitochondrial suspensions with ETH-129 in the absence (*top*) or presence (*bottom*) of SS-31 following the addition of NADH (*black arrowheads*) and CaCl_2_ (*open arrowheads*). See Fig. S18*A* for experimental details. *D*, *top*, TMRM traces of ΔΨ_m_ generation following the addition of 1 mm NADH (*black arrowhead*) in samples supplemented with 12.5 μm CaCl_2_ and vehicle only (control), 25 μm EDTA, SS-20, or SS-31 as indicated. *Bottom*, dose response of SS-31 on ΔΨ_m_ generation with 25 μm CaCl_2_ (defined in Fig. S18*D*); values shown are means ± S.D. (*error bars*) (*n* = 3). *E–G*, effect of SS-31 on external Ca^2+^ stress. *E* and *F*, ^31^P ssNMR analysis. Mitochondria (70 mg ml^−1^) were given acute treatment of SS-31 (0.6 mm) or vehicle only followed by calcium treatment (up to 5 mm CaCl_2_). *E*, static wide-line ^31^P NMR spectra of mitochondria subject to calcium stress (5 mm CaCl_2_) following treatment without (*left*) or with (*right*) SS-31. Spectral deconvolution yielded the indicated components (see Fig. S19, *A* and *B*), which were used to quantify the area ratios of L2/iso in *F* (values shown are means ± S.D. (*error bars*) (*n* = 3). *G*, respirometry analysis. Mitochondria (1 mg ml^−1^) were treated in the absence or presence of SS-31 (54.4 μm) or CaCl_2_ (1 mm) as indicated and measured for state 2 respiration (*n* = 10–12 each).

The final analyses of this study were focused on testing the functional implications of our observed effects of SS peptides on membrane surface electrostatics. We began with a theoretical consideration of how SS peptides may affect the decay of the electric field and ion distribution at the membrane interface based on GCS formalism (Fig. 7*A* and supporting Results H). By this analysis, SS peptides accumulate at the membrane surface with the effect of (i) reducing the magnitude of the surface charge and (ii) decreasing concentrations of other cationic species at the interface. This effect is particularly relevant for Ca^2+^, because mitochondria serve as high-capacity calcium stores in mediating cellular ion homeostasis and Ca^2+^ overload can cause severe damage to anionic lipid bilayers, particularly within mitochondria (see “Discussion”). This analysis shows that SS peptides can theoretically cause a strong reduction in surface accumulation of divalent cations, by over an order of magnitude. Hence, this model, albeit a simplified one, makes testable predictions about how SS peptides may mitigate divalent cation stress by reducing Ca^2+^ accumulation at the membrane interface. Our model was tested as follows.

First, we measured the effects of SS peptides on the accumulation of Ca^2+^ in the interfacial region of model membranes. We used a fluorescence-based approach with Calcium Green-5N (CG-5N), a membrane-impermeant probe that exhibits an emission intensity increase upon binding Ca^2+^ (characterized for our system in supporting Results H and Fig. S17). We reasoned that the binding of Ca^2+^ to the headgroup region of anionic lipid bilayers would reduce the bulk Ca^2+^ detected by the CG-5N probe, providing a direct readout of Ca^2+^ accumulation at the membrane interface. As a proof of principle, we titrated Ca^2+^ into solutions of CG-5N containing LUVs with monoanionic (POPG) or dianionic (TOCL) lipids at different concentrations (Fig. S17*B*). In the absence of LUVs, there was a saturating increase in CG-5N emission as the probe became maximally bound with Ca^2+^. However, the presence of POPG- and TOCL-containing LUVs caused a reduction in the CG-5N response to Ca^2+^ that was directly related to the amount of bilayer surface charge in the system. Hence, this assay provided a direct measure of Ca^2+^ sequestration within the headgroup region of anionic bilayers. To test the effects of SS peptides on Ca^2+^ binding to membrane surfaces, we first titrated SS-20 and SS-31 into solutions containing LUVs with 20% TOCL prebound with Ca^2+^ (Fig. S17*C*). SS-20 and SS-31 identically released Ca^2+^ from the membrane interface in a dose-dependent manner. Importantly, the CG-5N response began to saturate near P/L^eff^ of ∼1:10 (Fig. S17*C*, *green traces*), corresponding to the peptide binding saturation point, suggesting that Ca^2+^ release into the bulk corresponded to peptide-bilayer binding. As an independent assay, we performed Ca^2+^ titrations with solutions of LUVs that had been preincubated without peptide or with SS peptides at molar P/L^eff^ of 1:20 and 1:10 (Fig. 7*B*), again showing that SS peptides reduced the equilibrium binding of Ca^2+^ to anionic bilayer surfaces in a dose-dependent manner. Taken together, these results support a model in which SS peptide binding to bilayer surfaces reduces the accumulation of Ca^2+^ ions within the interfacial region of model membranes.

Second, we measured the effects of SS peptides on mitochondrial Ca^2+^ flux. External Ca^2+^ is taken up into the mitochondrial matrix electrophoretically by the ΔΨ_m_ (matrix negative). We reasoned that if SS peptides altered the accumulation of cations at the bilayer surface (Fig. 7*B* and Fig. S17*C*), then they may have a measurable effect on mitochondrial Ca^2+^ uptake. We therefore performed time course measurements of isolated mitochondria supplemented with respiratory substrate NADH followed by additions of calcium. In these experiments, we monitored the relative ΔΨ_m_ using the potentiometric probe tetramethylrhodamine methyl ester (TMRM) (the emission of which is inversely related to membrane potential; Fig. S17*E*) and, in parallel, monitored the external [Ca^2+^] using CG-5N (Fig. 7*C* and Fig. S18*A*). Note that because *S. cerevisiae* mitochondria do not contain a Ca^2+^ uniporter, Ca^2+^ uptake does not occur spontaneously but can occur in the presence of a calcium ionophore, for which we used ETH-129. These time courses show two key processes: (i) following NADH addition, there is a TMRM-detected establishment of the ΔΨ_m_, and (ii) after each calcium addition, there is a CG-5N-detected spike in external [Ca^2+^] followed by a decrease in CG-5N emission as Ca^2+^ is taken into the matrix, which is accompanied by a transient TMRM-detected partial depolarization and re-establishment of the ΔΨ_m_ (see Fig. S18*B* for expanded views of each of these transient processes). These experiments revealed two notable effects of SS peptides. The first was related to the establishment of the ΔΨ_m_ in samples containing ETH-129. Upon reaching a threshold potential, there was a temporal lag in potential generation, after which the mitochondria resumed establishing the maximal ΔΨ_m_. This is attributable to the energetic demands coupled to the uptake of external calcium. Interestingly, both SS-20 and SS-31 reduced the extent of this temporal delay in mitochondrial energization observed in the presence of increasing [Ca^2+^] (Fig. S18, *B* (ΔΨ*_m_ establishment*) and *C*). Quantitative analysis of Ca^2+^-dependent ΔΨ_m_ generation (described in Fig. S18*D*) revealed that SS-31 reduced this temporal delay in a dose-dependent and saturable manner (Fig. 7*D*). The second related effect was associated with subsequent calcium spikes. Following calcium additions, mitochondria transiently depolarized to near the same ΔΨ_m_ associated with the temporal lag. The kinetics of repolarization during the first calcium additions were faster in the presence of SS peptides; however, with increasing calcium load, the peptides no longer enhanced repolarization (see Fig. S18*A*, *Ca^2^*^+^ *transient 1–4*). By contrast, external calcium flux, judging by the extent of the CG-5N signal and by the kinetics of Ca^2+^ uptake, was not affected at all by SS peptides. Hence, under these conditions, SS peptides do not measurably alter the calcium storage capacity of mitochondria, but do improve the ability to respond energetically to calcium load. These results may provide a mechanistic underpinning to previous observations that acute treatment of isolated mitochondria with SS peptides protected against Ca^2+^-induced onset of the mitochondrial permeability transition (MPT) (8).

Finally, we measured the effects of SS peptides on mitochondrial calcium stress that may occur independently of Ca^2+^ uptake into the matrix. To this end, we used a ^31^P ssNMR–based strategy to monitor mitochondrial membrane structural integrity, based on previous studies using NMR analysis of membranes from prokaryotic cells (51) or isolated mitochondria (52–54). We first characterized ^31^P ssNMR spectra of our isolated mitochondria compared with model membranes (supporting Results H and Fig. S19), showing that mitochondria spectra contained a complex superposition of line shapes that could be deconvoluted for the direct analysis of phospholipid bilayers. Based on previous work using NMR analysis to measure the effects of calcium (up to 10 mm) on mitochondrial membranes (54), we then tested the ability of SS-31 to mitigate damage to mitochondrial membranes by calcium stress. We found that acute treatment of mitochondria with SS-31 (8.5 nmol of peptide/mg of mitochondrial protein) reduced NMR-detected membrane degradation caused by high calcium load (up to 5 mm CaCl_2_) (Fig. 7*E*). By spectral deconvolution, we observed that SS-31 treatment resulted in a significant preservation of the L2 peak, which corresponds to lamellar phospholipid bilayers (Figs. 7*F* and Fig. S19*A*). To corroborate these findings, we measured mitochondrial oxygen consumption rates under similar conditions of SS peptide treatment and calcium stress. Under these conditions, we observed a significant increase in basal respiration caused by calcium addition that was prevented by SS-31 pretreatment (Fig. 7*G*). Hence, acute SS-31 treatment rendered protective effects under these harsh conditions of calcium stress manifested as the preservation of membrane integrity and OXPHOS activity.

## Discussion

It is well-established that SS-31 specifically targets mitochondria and ameliorates the decrease in mitochondrial function associated with aging, cellular stress, and heritable diseases. Further, SS-31 is known to have affinity for aqueous dispersions of anionic lipids (12), particularly membranes containing CL (10). However, the nature of the interaction between SS-31 and mitochondrial membranes has remained elusive. Toward the goal of understanding the molecular MoA of SS-31, the present study addressed how this peptide interacts with lipid bilayers and the effect that it has on membrane properties. Our results support a mechanism in which the modulation of mitochondrial membrane surface electrostatic properties plays a critical role.

**Figure 8.**
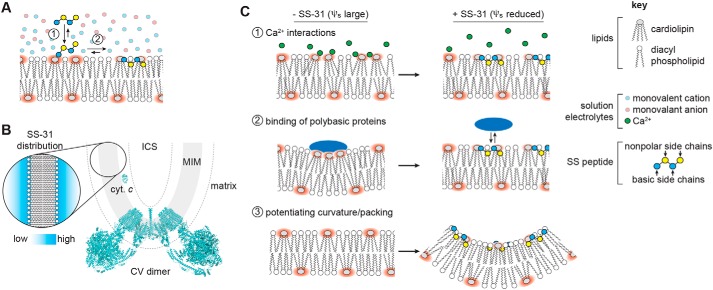
**SS peptide distribution and modulation of membrane surface electrostatics.** *A*, equilibrium binding of SS-31. *1*, polybasic peptide is drawn to the bilayer surface under the force of the electric field originating from negative surface charge density. *2*, peptide interaction stabilized by polar contacts (basic side chains and headgroup phosphates) and hydrophobic interactions (burial of nonpolar side chains in acyl chain region). *B*, depiction of SS peptide equilibrium distribution at mitochondrial membranes showing mitochondrial cristae region, including the MIM, which delineates aqueous matrix and intracristal space (*ICS*). Topological positioning of the amphitropic protein cytochrome *c* (*cyt. c*, Protein Data Bank entry 1YCC) and F_O_F_1_-ATP synthase (CV dimer, Protein Data Bank entry 6B8H) are shown for comparison. The *dashed line* represents a region of ∼30 Å (2–3 Debye lengths) from the membrane surface. *Inset*, representation of SS peptide distribution within the diffuse double layer. *C*, working models for how regulation of mitochondrial surface charge may underpin the efficacy of SS peptides. Divalent cations (*1*) and basic protein binding (*e.g.* cytochrome *c*) (*2*) cause lipid demixing and sequestration of CL with implications for membrane integrity, lipid-protein interactions, and lipid peroxidation. The binding of SS peptides to CL-containing bilayers shifts the equilibrium binding of cations and basic proteins away from the membrane surface (*3*). SS peptide binding alters physical properties of CL-containing bilayers. The ionized CL headgroup creates a strong local electric field (*red*) that alters lateral lipid interactions and creates a strong ψ_s_. Interfacial binding of SS-31 reduces ψ_s_, thereby enhancing lipid packing and promoting local curvature.

The first key finding of this study was on the lipid determinants of SS-31 membrane interactions, showing that (i) peptide binding depends on surface charge density, not the identity of particular component lipids; (ii) a key role of anionic headgroups is the establishment of a strong electric field that increases local concentration of SS peptides, enabling binding that is energetically dominated by hydrophobic contacts; and (iii) CL, although not strictly required for membrane interaction, is likely critical for SS peptide activity because it is the dominant anionic lipid of mitochondria and its structural polymorphism may be important for post-binding events in the SS peptide MoA (see supporting Discussion A). The second key finding was on the membrane binding equilibrium parameters of SS-31, including lipid binding densities (*n*) and affinities (*K_D_*) under different conditions. The relatively low affinity (*K_D_* in the micromolar range) determined for SS-31 is consistent with the interaction between a peptide and thermally disordered lipid bilayer and with the pharmacokinetic profile and cellular clearance reported previously (see supporting Discussion B). The third key finding was on the effects of SS peptides on membrane physical properties. Even at the highest concentrations used, SS peptides did not disrupt lamellar bilayer structural integrity; however, they did affect properties such as lipid packing and diffusivity and, most notably, membrane surface charge (and hence the ψ_s_) (see supporting Discussion C). We propose a binding model (Fig. 8*A*) in which the primary outcome of SS peptide binding is partial neutralization of charge on anionic lipids, which will thereby reduce the hydration, cross-sectional area, and Coulombic repulsion among headgroups and facilitate local increases in lipid packing. A critical corollary of this model is that this effect on surface electrostatics should be shared by all SS peptides due to their common positive charge density, and not depend on specific side-chain features such as the ROS-scavenging potential of the 2′,6′-Dmt moiety of SS-31. Indeed, we found that SS-20 and SS-31 are indistinguishable in terms of their effects on tuning membrane surface potential. A key feature of our binding model is that mitochondria-bound SS peptides will reside within the MIM diffuse double layer in a distribution governed by Boltzmann statistics, likely varying among mitochondrial subcompartments with different surface charge characteristics (Fig. 8*B* and supporting Discussion D).

How might the down-regulation of the ψ_s_ of anionic mitochondrial membranes underpin the broad therapeutic efficacy of SS peptides? We propose several nonexclusive possibilities (Fig. 8*C*). First, reducing the magnitude of the membrane surface charge will alter cation distribution at the interfacial region (Fig. 8*C*, *1*). This is particularly relevant for Ca^2+^ because mitochondria must accumulate large concentrations of this divalent cation in their role as cellular calcium stores. Ca^2+^ binding to the lipid headgroup region severely alters properties of anionic bilayers (53). More relevant to mitochondrial membranes, Ca^2+^ interacts strongly with CL, altering bilayer properties (*e.g.* demixing, phase behavior, and headgroup conformation) (21); Ca^2+^ can disrupt protein-lipid interactions, causing effects such as respiratory complex disintegration (55); and mitochondrial Ca^2+^ dyshomeostasis underpins a wide variety of mitochondrial diseases (56). In this study, we have directly shown that the binding of SS peptides reduces the effects of Ca^2+^ on mitochondrial surface electrostatics (Fig. 6*C*) and reduces the equilibrium binding of Ca^2+^ to the interface of anionic synthetic membranes, which act as a “sink” for divalent cations (Figs. 7*B* and Fig. S17 (*B* and *C*)). We also demonstrated that SS peptides appear to reduce the energetic burden associated with calcium uptake (Fig. 7, *C* and *D*) and preserve mitochondrial function and membrane integrity with calcium stress (Fig. 7, *E–G*). Although we do not directly show that the latter two physiological effects originate specifically from the reduction of Ca^2+^ accumulation at the bilayer surface, evidence that the site of action of SS peptides is at the membrane interface supports this as a likely mechanism. This proposed effect of SS peptides on mitigating bilayer Ca^2+^ accumulation is consistent with their demonstrated efficacy toward processes coupled to pathogenic mitochondrial Ca^2+^ overload, including ischemia-reperfusion, induction of the MPT, and apoptosis/necrosis (5). A second related mechanism is that reducing the magnitude of the ψ_s_ could reduce the mitotoxic interaction of basic proteins with CL-rich mitochondrial membranes (Fig. 8*C*, *2*). These include cytochrome *c* (*cyt. c*), whose binding to the MIM can alter lipid phase properties and cause lipid demixing (57) as well as lead to lipid peroxidation and initiate the intrinsic apoptotic pathway (58), and neurotoxic amyloid peptides, known to aggregate upon and damage mitochondrial membranes (59, 60). To this point, it is noteworthy that SS-31 has been shown to alter cytochrome *c* interactions with CL-containing bilayers (10, 11) and to reduce mitochondrial damage in models of Alzheimer's and Parkinson's disease (61, 62). Third, by down-regulating the surface charge density of mitochondrial membranes, SS peptides may act in part by altering the physical properties of CL-containing bilayers (Fig. 8*C*, *3*). Full ionization of CL phosphate groups creates strong electrostatic repulsion in the lipid headgroup region and increases the effective headgroup area, imparting a more cylindrical lipid geometry. Reduction of CL headgroup charge promotes more conical molecular geometry, thereby altering lipid packing interactions and making the establishment of membrane curvature more energetically accessible (22, 63–65). SS peptides may therefore help stabilize local regions of high curvature in the morphologically complex MIM. A final potential mechanism could relate to recent findings that dysfunctional mitochondrial protein complex assembly is coupled to the release of CL into the bulk lipid phase (66); under such pathological conditions, SS peptides could help regulate the resulting increase in membrane surface charge.

With these proposed mechanisms in mind, there are two ways to consider the broad efficacy of SS peptides in treating mitochondrial dysfunction. First, the controlled down-regulation of the mitochondrial ψ_s_ could have direct and specific impacts on different molecular pathomechanisms: for instance, by altering the molecular geometry of MLCL to impart more conical-like character, SS peptides could help stabilize cristae curvature in patients with Barth syndrome. But from a more general standpoint, SS peptides could also serve to reduce the constant stresses that mitochondria face under normal physiological conditions, including spikes in calcium levels and mitotoxic interactions between CL and polybasic proteins. Such stresses could present a manageable burden on mitochondria of healthy individuals but an excessively taxing one on mitochondria whose functional integrity is compromised, as occurs with aging and heritable mitochondrial disease. Hence, the remarkably broad effects of SS peptides in treating mitochondrial dysfunction could also be based partly on their ability to mitigate general molecular stressors at the membrane.

In summary, the results of this study support a model in which SS-31 binding to mitochondrial membranes is largely driven by, and subsequently down-regulates, the ψ_s_ as a core component of its molecular MoA. Considering SS peptides as mitochondria-targeted hydrophobic polyvalent cations, this model explains how these compounds act upon the general property of bilayer surface electrostatics to serve as broad-based therapeutic compounds for a wide array of mitochondrial disorders. Finally, it is noteworthy that a recent chemical cross-linking/MS-based study has identified several potential mitochondrial proteins with which SS-31 may interact (67); therefore, it remains possible that the SS peptide MoA may involve a combination of interactions with CL-enriched lipid bilayers and CL-associated proteins.

## Experimental procedures

### Reagents

Peptides SS-31 and SS-20 were prepared by solid-phase synthesis by Phoenix Pharmaceuticals (Burlingame, CA), and [ald]SS-31 was synthesized as described (12). All peptides were prepared as aqueous stocks. Synthetic phospholipids were purchased as chloroform stocks from Avanti Polar Lipids (Alabaster, AL), including POPC, POPG, and variants of cardiolipin with 18:1 acyl chains, including TOCL and trioleoyl MLCL. All lipid stocks were stored at −20 °C in clear vials with Teflon-lined cap closures until use. Fluorescent probes TMRM, laurdan, prodan, DPH, ANS, and CG-5N were purchased from Molecular Probes (Thermo Fisher Scientific). Solutions were prepared with deionized ultrapure water, and all buffers, salts and other reagents were obtained from Sigma–Aldrich.

### Liposome preparation

Liposomes of a specific lipid composition were prepared by mixing chloroform stocks at the appropriate molar ratios in glass vials and drying under N_2_ flow, followed by incubation in a vacuum desiccator for a minimum of 2 h to remove all organic solvent. Lipid films were hydrated in aqueous solutions to produce dispersions of multilamellar vesicles. To prepare LUVs, lipid suspensions were extruded through polycarbonate membranes with 0.1-μm pores (Whatman, Maidstone, UK) (68). Multilamellar vesicles and LUVs were prepared in aqueous solutions of buffer (20 mm HEPES-KOH, pH 7.5) in the presence or absence of different concentrations of salt (KCl), depending on the application. Hydration and extrusion steps were all performed at a temperature above the main (gel to liquid phase) temperature of the highest-melting lipid. The phospholipid concentration of each preparation was determined by a colorimetric ammonium ferrothiocyanate assay (69) against standards based on the mole fraction of lipids of a given preparation. Liposome suspensions were routinely monitored for size and monodispersity by dynamic light scattering.

### Yeast strains and isolation of mitochondria

The yeast strains used in this study are isogenic to GA74-1A, including genetic knockouts Δ*crd1* and Δ*taz1*, which were described previously (50). Yeast cells were grown at 30 °C in lactate medium containing 0.1% glucose. For the preparation of active mitochondria, yeast cells were cultivated to mid-log phase, and mitochondria were isolated by Dounce homogenization of spheroplasts and differential centrifugation as described (70). Isolated mitochondria were resuspended in buffer (600 mm sorbitol, 20 mm HEPES-KOH, 1 mm EDTA, pH 7.5), and aliquots (1–2 mg of mitochondrial protein each) were snap-frozen in liquid nitrogen and stored at −80 °C. Mitoplasts were prepared by osmotically rupturing the outer membrane as described (70) by incubating intact mitochondria in buffer (60 mm sorbitol, 20 mm HEPES, pH 7.5) on ice for 30 min.

### Analytical fluorescence spectroscopy

Steady-state fluorescence measurements were performed with a Fluorolog 3-22 spectrofluorometer (HORIBA Jobin-Yvon) equipped with photon-counting electronics, double-grating excitation and emission monochromators, automated Glan–Thompson polarizers, and a 450-watt xenon lamp. Measurements were made either in 4 × 4-mm quartz microcells or in 1 × 1-cm quartz cuvettes with a stir disc seated in a thermostated cell holder. For all spectral measurements, samples containing the fluorescent moiety (peptide or extrinsic fluorophore) were recorded in the L-configuration. Endogenous fluorescence measurements were made in parallel with blanks (lacking fluorophore but otherwise identical), which were used for background subtraction to obtain the signal originating solely from the fluorescent molecule. The following steady-state measurements were made. (*a*) Endogenous fluorescence from the 2′,6′-Dmt moiety of the native SS-31 peptide was measured by emission (λ_ex_ = 283 nm; λ_em_ = 290–400 nm) or excitation (λ_ex_ = 260–300 nm; λ_em_ = 308 nm) scans using 1-nm increments and 2-s integration times. To suppress scattered light from lipid-containing samples and eliminate spectral distortions, in-line polarizers were used in the cross-oriented configuration (Ex^pol^ = 90°, Em^pol^ = 0°) as described (71). (*b*) Endogenous steady-state anisotropy (〈*r*〉) was determined by measuring emission intensities at four different polarizer orientations (excitation with vertical polarization, *I*_VV_ and *I*_VH_, and excitation with horizontal polarization, *I*_HV_ and *I*_HH_). Each sample was background-subtracted with the cognate blank to measure the *G*-factor (*G* = *I*_HV_/*I*_HH_) and the steady-state anisotropy 〈*r*〉 = (*I*_VV_ − *GI*_VH_)/(*I*_VV_ + 2*GI*_VH_). The steady-state anisotropy of 2′,6′-Dmt was determined using λ_ex_ = 283 nm; λ_em_ = 308 nm, and the steady state anisotropy of DPH was determined using λ_ex_ = 360 nm; λ_em_ = 430 nm. (*c*) Dynamic (collisional) quenching of endogenous fluorescence was performed by titration of quenching agent (Q) and measurement of steady-state fluorescence (*F*) relative to unquenched fluorescence (*F*_0_) with linear fits of (*F*_0_/*F*) − 1 *versus* [Q] based on Stern–Volmer formalism, (*F*_0_/*F*) − 1 = *K*_SV_[Q], to yield the Stern–Volmer constant, *K*_SV_. (*d*) Fluorescence measurements of extrinsic fluorophores were performed by emission scans (1-nm increments with 1–2-s integration times) or by kinetic measurements (0.5–1-s integration times) as follows. Aladan fluorescence from the [ald]SS-31 peptide was measured by emission scans (λ_ex_ = 360 nm; λ_em_ = 400–600 nm) with excitation and emission bandpass at 4 nm. The fluorescence of laurdan and prodan-containing samples was measured by emission scans (λ_ex_ = 340 nm; λ_em_ = 370–600 nm) with excitation and emission bandpass at 2 nm. Emission intensities at 440 nm and 490 nm (*I*_440_ and *I*_490_, representing emission maxima of pure L_β_ and L_α_ phases, respectively) were used to calculate the generalized polarization (GP) of laurdan as GP^LAU^ = (*I*_440_ − *I*_490_)/(*I*_440_ + *I*_490_). The GP of prodan was calculated by the three-wavelength excitation GP (3wGP) (72) as GP^PRO^ = (*R*_12_ − 1)/(*R*_12_ + 1), where *R*_12_ = *I*_420_*k*/(*I*_480_*k* − *I*_530_); *I*_420_, *I*_480_, and *I*_530_ are emission intensities at 420, 480, and 530 nm; and *k* is a constant independently verified to be 2.8. The fluorescence of ANS was measured by time course (λ_ex_ = 380 nm; λ_em_ = 460 nm) or emission scan (λ_ex_ = 380 nm; λ_em_ = 400–500 nm) measurements with excitation and emission bandpass at 4 nm. The fluorescence of CG-5N was measured by time course (λ_ex_ = 506 nm; λ_em_ = 532 nm) or emission scan (λ_ex_ = 506 nm; λ_em_ = 512–562 nm) measurements with excitation and emission bandpass at 2 nm. And the fluorescence of TMRM was measured by time course measurements (λ_ex_ = 546 nm; λ_em_ = 573 nm) with excitation and emission bandpass at 4 nm.

Time-resolved fluorescence lifetime measurements were performed with a Fluorolog 3-22 instrument equipped with a TCSPC module and pulsed nano-LED light source (peak wavelength 280 nm ± 10 nm; pulse width <1.2 ns).

### Microcalorimetry

ITC measurements were performed as described (35) with a low-volume nano-ITC microcalorimeter (TA Instruments, New Castle, DE). Solutions of SS-31 (titrate) and LUVs (titrant) were prepared in degassed buffer, equivalent to that used for liposome preparation. Lipid-into-peptide titrations were performed with 87.5–175 μm SS-31 in the calorimeter cell (volume 170 μl), and LUVs (8 mm total lipid) were injected in aliquots of 2.5 μl (20 total injections) at time intervals of 300 s at 25 °C. To account for heats of dilution, experiments were performed by the addition of titrant into solutions of buffer only, which were used for baseline subtraction. Data from dilution-corrected and integrated heat flow time courses were fit as Wiseman plots, from which equilibrium binding and thermodynamic parameters (*K_A_*, *n*, Δ*H*, and Δ*S*) were determined by nonlinear regression fits (NanoAnalyze software version 3.10.0, TA Instruments).

### NMR spectroscopy

NMR data were recorded at 162 MHz on a Bruker AVANCE III 400-MHz WB spectrometer equipped with an HXY probe tuned for ^1^H and ^31^P. High-power decoupled spectra were acquired with a recycle delay of 2 s, a 90° pulse of 5 μs, and a 50-kHz decoupling field. All ^31^P NMR spectra were externally referenced by assigning the 85% H_3_PO_4_ peak to 0 ppm. Model membranes (LUVs) were measured at 25 °C (total of 10,240 scans/sample) with a total lipid concentration of 2 mm lipid in the presence of different concentrations of SS-31 (0, 1:500, 1:50, and 1:5 [P]/[L]^eff^). Isolated mitochondria were measured in minimal buffer (600 mm sorbitol, 20 mm HEPES-KOH, pH 7.5) at 4 °C (total of 512 scans/sample) at a total concentration of 70 mg of mitochondrial protein ml^−1^ with different concentrations of SS-31 (up to 3 mm) and/or [CaCl_2_] (up to 5 mm). Peak fitting and deconvolution were performed using the software dmFit (73).

### SAXS

SAXS scattering curves (scattered intensity, *I*(*q*), as a function of moment transfer, *q*) were recorded at the LiX beamline of the National Synchrotron Light Source II (NSLS-II), Brookhaven National Laboratory, operating at 13 keV. LUV samples (2 mm total lipid) were measured in 1.5-mm diameter quartz flow-through capillaries using a 1-s exposure, and the *q*-range covered was 0.005–3.2 Å^−1^.

### Electrokinetic measurements

Measurements of zeta potential (ζ) of LUVs were performed as we have described (74), using a Zetasizer Nano ZS (Malvern). LUVs (100 μm total lipid) were added to the sample cell (final volume, 1 ml) with different concentrations of SS-31. Values of ζ represent the electrostatic potential ψ_x_ at the hydrodynamic shear plane of lipid vesicles based on the measured electrophoretic mobility (μ) by the Helmholtz–Smoluchowski relationship,

(Eq. 1) $$\zeta =\frac{\mu \eta }{\epsilon_{\text{r}}\epsilon_{0}}$$

where η is the viscosity of the solution, ϵ_r_ is the dielectric constant of the aqueous phase, and ϵ_0_ is the permittivity of free space. Calculation of surface potential (ψ_0_) was based on the distance-dependent decay of electrostatic potential from the membrane surface,

(Eq. 2) $$\kappa x=\ln\left(\frac{\left(\exp\left(\frac{ZF\zeta }{2RT}\right)+1\right)\left(\exp\left(\frac{ZF\psi_{0}}{2RT}\right)-1\right)}{\left(\exp\left(\frac{ZF\zeta }{2RT}\right)-1\right)\left(\exp\left(\frac{ZF\psi_{0}}{2RT}\right)+1\right)}\right)$$

where *R* is the molar gas constant, *T* is the absolute temperature, *Z* is ionic charge of solution electrolyte, *x* is the distance from the membrane surface, and κ (the Debye constant) is as follows.

(Eq. 3) $$\kappa^{2}=\frac{2Z^{2}F^{2}C_{i\infty }}{\epsilon_{\text{r}}\epsilon_{0}RT}$$

The electrostatic shear plane was assumed to be at *x* = 2 Å.

### Mitochondrial activity assays

Mitochondrial calcium uptake was measured by monitoring the bulk [Ca^2+^] using the cell-impermeant calcium-indicating fluorophore CG-5N (prepared as an aqueous stock), adapted from a protocol described for measuring Ca^2+^ uptake into *S. cerevisiae* mitochondria (75). Reactions containing mitochondria (100 μg) in calcium flux buffer (300 mm sucrose, 20 mm HEPES-KOH, 2 mm KH_2_PO_4_, 0.5 mg ml^−1^ BSA, pH 7.5) were prepared with the probe (1 μm CG-5N) in the presence or absence of calcium ionophore (5 μm ETH-129) and variable concentrations of CaCl_2_. Measurements of mitochondrial membrane potential were made using the potentiometric fluorophore TMRM under identical conditions, but in the presence of 50 nm TMRM (prepared as a methanol stock). CG-5N and TMRM measurements were performed as time course measurements in stirred cuvettes with the progressive addition of respiratory substrate (1 mm NADH) and CaCl_2_ at specified time intervals in the presence or absence of the indicated concentrations of SS peptides.

Oxygen consumption rates of isolated mitochondria were measured using a closed, thermostated chamber equipped with a Clark-type oxygen electrode (Oxygraph Plus System, Hansatech Instruments, UK). Reactions were set up with 100 μg of mitochondria in one of two respiration measurement buffers. Standard respiration buffer (300 mm sucrose, 20 mm HEPES-KOH, 10 mm KH_2_PO_4_, 10 mm KCl, 5 mm MgCl_2_, 1 mg ml^−1^ BSA, 0.5 mm EDTA, pH 7.5) was used for routine testing of mitochondrial activity and coupling. Minimal respiration buffer (300 mm sucrose, 20 mm HEPES-KOH, 1 mg ml^−1^ BSA, 10 mm KH_2_PO_4_, pH 7.5) was used for measurements under NMR conditions. To test the effects of calcium stress and/or SS peptide on mitochondrial respiration, a concentrated stock of mitochondria (1 mg ml^−1^) was preincubated with CaCl_2_ (1 mm, equivalent to 1 μmol of Ca^2+^/mg of mitochondria) and/or SS-31 (54.4 μm, equivalent to 54.4 nmol of SS-31/mg of mitochondria) on ice for 20 min prior to polarographic measurements. The effect of calcium stress on mitochondrial respiration was measured by monitoring state 2 respiration for 3 min following the addition of respiratory substrate (19 mm succinate and 1 mm NADH) and by monitoring respiration without noncatalytic H^+^ flux through the F_O_ of ATP synthase for 3 min following the addition of 14.4 μm oligomycin.

### Lipidomics

Multidimensional MS-based shotgun lipidomic analysis of mitochondrial lipids was performed as described (50, 76). In brief, a mixture of internal standards was added to isolated mitochondria for quantitation of phospholipid species based on the content of mitochondrial protein, lipids were extracted using a modified Bligh and Dyer procedure (77), and each lipid extract was reconstituted in 1:1 (v/v) chloroform/methanol at a volume of 200 μl mg^−1^ mitochondrial protein. For shotgun lipidomics, lipid extracts were diluted to a final concentration of ∼500 fmol of total lipids μl^−1^. Mass spectrometric analysis was performed on a triple quadrupole mass spectrometer (TSQ Altis, Thermo Fisher Scientific) and a Q Exactive mass spectrometer (Thermo Scientific), both of which were equipped with an automated nanospray device (TriVersa NanoMate, Advion Bioscience Ltd., Ithaca, NY) as described (78). Identification and quantification of phospholipid species were performed using an automated software program (79, 80). Data processing (ion peak selection, baseline correction, data transfer, peak intensity comparison, and quantitation) was performed as described (80).

### MD simulations

The SS-31 peptide structure was generated by modifying an extended tetrapeptide with sequence Arg-Tyr-Lys-Phe. Coordinates were modified using the VMD Molefacture plugin (81) to invert the stereochemistry of the N-terminal Arg residue (from l to d) and to replace the 2′ and 6′ hydrogen atoms of the Tyr side chain with methyl groups. Parameters for the 2′,6′-Dmt were modeled after the output parameters of 3′,5′-dimethylphenol after running its structure through ParamChem's CGenFF server (82). The fully parameterized SS-31 peptide was then run through the CGenFF server again to check for energy penalties.

MD simulations were then used to characterize the binding mechanism of SS-31 and to investigate its effects on membrane dynamics. All-atom systems with explicit membrane and solvent were prepared using CHARMM-GUI with the CHARMM-36m forcefield and the TIP3 water model (83–89). Bilayers were generated with molar ratios of TOCL/POPC (20:80), MLCL/POPC (20:80), and POPG/POPC (20:80). Each system contained a total of 300 lipids (150 per leaflet). As done in a previous study (30), the MLCL system was created from the TOCL system by replacing an *sn*-2 acyl chain with a hydroxyl, appending the topology, and using a GROMACS-compatible topology file (.itp) for MLCL. Following the CHARMM-GUI standard protocol for membranes, the bilayer systems were energy-minimized using the steepest-descent algorithm for 5000 steps, followed by canonical ensemble equilibration for 50 ps with 1-fs timestep, and 325 ps of NPT equilibration with a 2-fs timestep accomplished using the semiisotropic pressure-coupling scheme and the Berendsen barostat. Position and dihedral restraints were used during equilibration on the lipids to maintain lipid geometry and bilayer morphology. Production simulations of the bilayers alone were each run for 1 μs in an NVT ensemble with a timestep of 2 fs. In our simulations, we define the “upper leaflet” as the side of the membrane exposed to peptides and the “lower leaflet” as the opposing side, which was not exposed to peptides.

Peptide-bilayer systems were constructed by removing solvent from the equilibrated bilayers, placing 10 peptides 1–3 nm away from the upper leaflet headgroup region, and then resolvating and adding neutralizing sodium ions. The sizes of the systems were ∼88,000 atoms for the TOCL systems, ∼85,000 atoms for the MLCL systems, and ∼73,000 for the POPG systems. The systems were then energy-minimized for 5000 steepest-descent steps, followed by canonical ensemble equilibration for 50 ps with a 1-fs timestep and 425 ps of NPT equilibration with a 2-fs timestep accomplished using the semiisotropic pressure-coupling scheme and the Berendsen barostat. Position and dihedral restraints were used during equilibration on the lipids and peptides to maintain lipid geometry and bilayer morphology and to prevent the peptides from interacting with the bilayers during equilibration. Production simulations were run for 1.6 μs and saved every 50 ps for the GROMACS mean squared displacement and radial distribution function analyses, every 100 ps for GROMACS solvent-accessible surface area analyses, and every 250 ps for the MDTraj analyses. The first 50 ns of the production simulations were omitted from all analyses except the binding time dependence (Fig. 5*A* and Fig. S11*A*). To enforce peptide binding to only the upper leaflet (*i.e.* to impose an energy penalty on peptides crossing the *z*-dimension periodic boundary and binding to lower leaflet), two inverted flat-bottom restraints in the *z*-direction were placed ∼1 nm away from the headgroup region of the lower leaflet. A restraint was placed on the peptides with a force constant of 1000 kJ mol^−1^ nm^−1^ and a radius of 2 nm, which served to prevent peptides from binding to the lower leaflet. A second restraint was placed on the lower leaflet phosphates of POPC with a force constant of 200 kJ mol^−1^ nm^−1^ and a radius of 0.5 nm to prevent the lower leaflet from drifting in the negative *z*-direction while still allowing for natural membrane deformations.

All simulations were performed using GROMACS 2016 (90, 91). Electrostatic and Lennard–Jones interactions were cut off at 1.2 nm, with electrostatics shifted from 0 nm to the cutoff and Lennard–Jones interactions shifted from 1.0 nm to the cutoff. All production runs were simulated in the NPT ensemble using the Parrinello–Rahman coupling scheme, with the temperature maintained at 303.15 K and pressure kept at 1.0 bar with semiisotropic coupling. The time constants for pressure and temperature couplings were 5.0 and 1.0 ps, respectively, and the compressibility value was set to 4.5 × 10^−5^ bar^−1^. Simulations were performed using periodic boundary conditions in all dimensions, and the simulation time step was 2 fs.

### Statistical analysis

All reported means are the average values of a minimum of three measurements from independent experimental samples. Statistical comparisons were performed by *t* test (not significant (*ns*), *p* > 0.05; •, *p* < 0.05; ••, *p* < 0.01; •••, *p* < 0.001; ••••, *p* < 0.0001).

## Data availability

All data are contained within the article.

## Author contributions

W. M., E. A. N., J. D. T., K. J. B., M. S., A. C., E. R. M., and N. N. A. conceptualization; W. M., E. A. N., J. D. T., K. J. B., M. S., A. C., M. P., X. H., N. A. E., E. R. M., and N. N. A. data curation; W. M., E. A. N., J. D. T., K. J. B., M. S., A. C., M. P., X. H., N. A. E., E. R. M., H. H. S., and N. N. A. formal analysis; W. M., E. A. N., J. D. T., K. J. B., E. R. M., and N. N. A. validation; W. M., E. A. N., J. D. T., K. J. B., M. S., A. C., M. P., X. H., N. A. E., E. R. M., and N. N. A. investigation; W. M., E. A. N., J. D. T., K. J. B., M. S., A. C., M. P., X. H., N. A. E., E. R. M., and N. N. A. methodology; W. M., E. A. N., J. D. T., K. J. B., E. R. M., H. H. S., and N. N. A. writing-original draft; W. M., E. A. N., J. D. T., K. J. B., E. R. M., H. H. S., and N. N. A. writing-review and editing; E. R. M. and N. N. A. supervision; E. R. M. and N. N. A. resources; E. R. M. and N. N. A. funding acquisition; N. N. A. visualization; N. N. A. project administration.

## Supplementary Material

Supporting Information

## Footnotes

5 The abbreviations used are:

OXPHOS: oxidative phosphorylation
ROS: reactive oxygen species
2′,6′-Dmt: 2′,6′-dimethyltyrosine
ANS: 1-anilinonaphthalene-8-sulfonic acid
CL: cardiolipin
DPH: 1,6-diphenyl-1,3,5-hexatriene
GCS: Gouy-Chapman-Stern
GP: generalized polarization
ITC: isothermal titration calorimetry
LUV: large unilamellar vesicle
MD: molecular dynamics
MIM: mitochondrial inner membrane
MLCL: trioleoyl monolysocardiolipin
MoA: mechanism of action
MPT: mitochondrial permeability transition
PC: phosphatidylcholine
PG: phosphatidylglycerol
POPG: 1-palmitoyl-2-oleoyl-*sn*-glycero-3-phosphoglycerol
POPC: 1-palmitoyl-2-oleoyl-*sn*-glycero-3-phosphocholine
SAXS: small-angle X-ray scattering
SS peptides: Szeto–Schiller peptides
TMRM: tetramethylrhodamine methyl ester
TOCL: 1′,3′-bis[1,2-dioleoyl-*sn*-glycero-3-phospho]-glycerol (tetraoleoyl cardiolipin)
ssNMR: solid-state NMR
CG-5N: Calcium Green-5N
laurdan: 6-dodecanoyl-2-dimethylaminonaphthalene
prodan: 6-propinoyl-2-dimethylaminonaphthalene.
