# Supplementary material for: The mitochondria-targeted peptide SS-31 binds lipid bilayers and modulates surface electrostatics as a key component of its mechanism of action
Source: J Biol Chem. 2020 Apr 9;295(21):7452–69. doi: 10.1074/jbc.RA119.012094 (PMC7247319; doi:10.1074/jbc.RA119.012094)
Supplement: Supporting Information [file supp_295_21_7452__index.html]

The mitochondria-targeted peptide SS-31 binds lipid bilayers and modulates surface electrostatics as a key component of its mechanism of action — SS-31 modulates lipid bilayer surface electrostatics — Supporting Information 

# The mitochondria-targeted peptide SS-31 binds lipid bilayers and modulates surface electrostatics as a key component of its mechanism of action

## Supporting Information

- Supporting Information (to be published online) - Supporting Information
