# Supplementary material for: The mitochondria-targeted peptide SS-31 binds lipid bilayers and modulates surface electrostatics as a key component of its mechanism of action
Source: J Biol Chem. 2020 Apr 9;295(21):7452–69. doi: 10.1074/jbc.RA119.012094 (PMC7247319; doi:10.1074/jbc.RA119.012094)
Supplement: Supporting Information [file supp_RA119.012094_157400_2_supp_506728_q8f1dr.pdf]

SUPPORTING INFORMATION

The mitochondria-targeted peptide SS-31 binds lipid bilayers and modulates surface electrostatics as a key component of its mechanism of action

Wayne Mitchell, Emily A. Ng, Jeffrey D. Tamucci, Kevin J. Boyd, Murugappan Sathappa, Adrian Coscia, Meixia Pan, Xianlin Han, Nicholas A. Eddy, Eric R. May, Hazel H. Szeto, and Nathan N. Alder

Table of Contents

**Supporting Information - Analytical Approaches**	**Page**
A. Quantitative analysis of peptide binding isotherms	1
B. Quantitative analysis of surface electrostatics	3
C. Nonlinear least squares fitting	4
D. Analysis of MD simulations	4
**Supporting Information - Results**	
A. Spectral characterization of SS-31 in solution	5
B. Fluorescence-based analysis of SS-31 membrane interactions	6
C. Quantitative analysis of SS-31 membrane binding isotherms	9
D. SS-31 titration time course analysis and ITC	9
E. Model membrane surface electrostatics and SS-31 binding	10
F. Assays for peptide-induced structural polymorphism of model membranes	11
G. Molecular dynamics analyses	14
H. Assays for measuring Ca^{2+} interactions with model membranes and mitochondria	17
**Supporting Information - Discussion**	
A. Lipid determinants of SS peptide binding	20
B. SS-31 equilibrium binding parameters	22
C. Effects of SS peptides on membrane physical properties	22
D. SS peptide mitochondrial distribution	23
**Supporting Information References**	24
**Supporting Information - Figures**	
Figure S1. Cardiolipin structure and biogenesis	28
Figure S2. The binding of amphipathic peptides to anionic membranes	29
Figure S3. Fluorescence characterization of SS-31 in aqueous buffer	30
Figure S4. Fluorescence characterization of SS-31 membrane interactions	31
Figure S5. Quantitative analysis of peptide membrane binding	32
Figure S6. Kinetic measurements of SS-31 addition to LUVs	33
Figure S7. Analysis of time course binding saturation measurements	34
Figure S8. Microcalorimetry analysis of SS-31 membrane binding	35
Figure S9. Relationship between SS peptide binding, ionic strength, and surface potential	36
Figure S10. Characterization of the effects of SS-31 on model membranes	37
Figure S11. Molecular dynamics simulations of SS-31 side chain insertion depths	38
Figure S12. Lipid radial distribution profiles from MD simulations	39
Figure S13. MSD of lipids with and without SS-31 from MD simulations	40
Figure S14. Acyl chain solvent accessible surface area from MD simulations	41
Figure S15. [ald]SS-31 binding isotherms with model membranes	42
Figure S16. Binding of [ald]SS-31 to isolated mitochondria	43
Figure S17. Characterization of fluorescence-based assays of calcium dynamics	44
Figure S18. Kinetic responses of $\Delta\Psi_m$ and external $[Ca^{2+}]$	45
Figure S19. NMR and respirometry measurements with calcium stress	46

<u>Supporting Information - Analytical Approaches</u>

A. Quantitative analysis of peptide binding isotherms

1. Fitting saturation binding curves to obtain parameters n and K_D

Models for the binding of SS-31 to membranes are based on the independent and reversible binding of free peptide in solution (P_f) to an accessible lipid binding site (M_f) that is composed of n free lipids (L_f) to form a peptide-membrane complex (PM):

$$[P]_f + [M]_f \rightleftarrows PM \qquad \textit{Eq. S1}$$

Here, the total number of peptide binding sites is related to the lipid concentration by $[M] = [L]_0/n$, where $[L]_0$ is the total concentration of accessible lipids. The equilibrium dissociation constant (K_{D}, in units of *M*) associated with this binding is:

$$K_{\mathrm{D}} = \frac{[P]_f[M]_f}{[PM]}$$

Binging isotherm data were fit to quadratic expansions of the Langmuir adsorption isotherm equation (1) to extract equilibrium binding data as follows:

For experiments in which a fixed concentration of L was titrated with P, binding data were fit by the following equation:

$$\theta = \frac{([P]_0 + [L]_0/n + K_D) - \sqrt{([P]_0 + [L]_0/n + K_D)^2 - (4[P]_0[L]_0/n)}}{2([L]_0/n)} \qquad \textit{Eq. S2}$$

In this equation, θ is the fractional saturation, $[P]_0$ and $[L]_0$ are the total P and accessible L concentrations, respectively, n is the lipid:peptide stoichiometry, and K_{D} is the equilibrium dissociation constant. In these experiments, the total number of peptide binding sites ($[L]_0/n$) is constant and θ is a measure of the fraction of lipid binding sites bound to peptide. Note that when binding saturation data are fit to Eq. S2, K_{D} is expressed in terms of molarity of peptide *binding sites* (not individual lipids); therefore, the term nK_{D} expresses the affinity of peptide for lipid monomers.

For experiments in which a fixed concentration of P was titrated with L, binding data were fit by:

$$\theta = \frac{(n[P]_0 + [L]_0 + K_D) - \sqrt{(n[P]_0 + [L]_0 + K_D)^2 - (4n[P]_0[L]_0)}}{2(n[P]_0)} \qquad \textit{Eq. S3}$$

In this equation, the number of accessible lipids required to bind all peptide $(n[P]_0)$ is constant and θ is a measure of the fraction of peptides bound. Example data and analysis of data fits to Langmuir isotherms

are shown for peptide titration (Figure S5 A,B; data fits based on Eq. S2) and for lipid titration (Figure S5 D,E; data fits based on Eq. S3).

2. Scatchard analysis

As an independent means of analysis, binding data were also evaluated by Scatchard formalism (2) as follows. Given the definition of the equilibrium association constant (K_{A}, in units of M^{-1}) in terms of peptide-lipid complex ($[PL]$) , free peptide ($[P]_f$) and free lipid ($[L]_f$):

$$K_{\mathrm{A}} = \frac{[PL]}{[P]_f[L]_f} \qquad \textit{Eq. S4}$$

This equation can be recast to include bound peptide ($[P]_b$) and bound lipid ($[L]_b$). In the case of peptide titrations (with constant lipid), given that $[L]_b/n = [PL]$ and that $[P]_f = [P]_0 - [P]_b$, Eq. S4 is rearranged such that:

$$\frac{[L]_b}{[L]_f} = nK_{\mathrm{A}}[P]_0 - nK_{\mathrm{A}}[P]_b \qquad \textit{Eq. S5}$$

Given the relationship $[P]_b = [L]_b/n$, Eq. S5 becomes

$$\frac{[L]_b}{[L]_f} = nK_{\mathrm{A}}[P]_0 - K_{\mathrm{A}}[L]_b$$

which can be rearranged as

$$\frac{[L]_b}{[L]_f[P]_0} = K_{\mathrm{A}}\left(n - \frac{[L]_b}{[P]_0}\right) \qquad \textit{Eq. S6}$$

Therefore, a plot of $[L]_b/[L]_f[P]_0$ *versus* $[L]_b/[P]_0$ yields a line with slope of $-K_{\mathrm{A}}$, a y-intercept of nK_{A}, and an x-intercept of n (Figure S5C).

In the case of lipid titrations (with constant peptide), given that $[P]_b = [PL]$ and that $[L]_f = [L]_0 - [L]_b$, Eq. S4 is rearranged such that:

$$\frac{[P]_b}{[P]_f} = K_{\mathrm{A}}[L]_0 - K_{\mathrm{A}}[L]_b \qquad \textit{Eq. S7}$$

Given the relationship $[L]_b = n[P]_b$, Eq. S7 becomes

$$\frac{[P]_b}{[P]_f} = K_{\mathrm{A}}[L]_0 - K_{\mathrm{A}}n[P]_b$$

which can be rearranged as

$$\frac{[P]_b}{[P]_f[L]_0} = K_{\mathrm{A}}\left(1 - n\frac{[P]_b}{[L]_0}\right) \qquad \textit{Eq. S8}$$

Therefore, a plot of $[P]_b/[P]_f[L]_0$ *versus* $[P]_b/[L]_0$ yields a line with slope of $-nK_\mathrm{A}$, a y-intercept of K_A, and an x-intercept of n^{-1} (Figure S5F).

3. Partition coefficients

Peptide binding isotherms from lipid titrations were independently fit to obtain the molar partition coefficient K_P as described (3), in accordance with the equation

$$F = \frac{F_0 + K_\mathrm{P}\gamma_\mathrm{L}[L]_0 F_\mathrm{L}}{1 + K_\mathrm{P}\gamma_\mathrm{L}[L]_0} \qquad Eq.\ S9$$

in which F is the measured fluorescence intensity at a given lipid concentration, $[L]_0$; F_0 and F_L are, respectively, the peptide fluorescence intensities in the absence of lipid (water only) and the fluorescence when completely lipid-bound (determined from F vs. $[L]_0$ plots); and γ_L is the mol fraction-weighted average of lipid molar volume. The partial molar volumes used in this equation were γ_POPC = 0.7564 M^{-1}, γ_POPG = 0.7238 M^{-1}, and γ_TOCL = 1.4453 M^{-1} from published values (4-6). The corresponding value for MLCL is not available, so we made the assumption that γ_MLCL = γ_TOCL.

B. Quantitative analysis of surface electrostatics

The electrostatic profile within the diffuse double layer of our model membranes was evaluated as we have described (7) using Gouy-Chapman-Stern formalism (8,9). The relationship between the charge density at the membrane surface (σ, in C m^{-2}) and the electrostatic potential of the aqueous phase at the surface of the membrane (ψ_0, in V) is described by a variation of the Gouy-Chapman equation:

$$\sigma = \frac{\psi_0}{|\psi_0|}\left[2\varepsilon_r\varepsilon_0 RT\sum_i C_{i\infty}\left(\exp\left(\frac{-Z_i F\psi_0}{RT}\right) - 1\right)\right]^{1/2} \qquad Eq.\ S10$$

where ε_r is the dielectric constant, ε_0 is the permittivity of free space, R is the molar gas constant, T is the absolute temperature, $C_{i\infty}$ is the bulk concentration of ionic species i, F is the Faraday constant, and Z_i is the formal charge on ionic species i. The distribution of ions within the interfacial region and normal to the membrane surface is determined by Boltzmann statistics, wherein the relationship between $C_{i\infty}$ and its surface concentration ($C_{0,i}$) is given by:

$$C_{0,i} = C_{i\infty}\exp\left(\frac{-Z_i F\psi_0}{RT}\right) \qquad Eq.\ S11$$

The adsorption of ions to the charged membrane surface is described by a Langmuir isotherm:

$$\sigma = \frac{\sigma^{\max}}{1 + K_{\mathrm{M}} C_{0,i}} \qquad Eq.\ S12$$

where $\sigma^{\max}$ is the maximum charge density of the membrane surface, determined by the mole fraction-weighted lipid headgroup formal charge and cross-sectional areas, and K_{M} is the association constant that describes the adsorption of solution ions.

The simultaneous evaluation of Eq. S10-S12 allows for the determination of ψ_0 as a function of $\sigma^{\max}$, $C_{i\infty}$, and K_{M}. This analysis was used to evaluate ψ_s (surface potential) under conditions of different lipid composition and bulk ionic strength (Fig. 3A, S9C) and to evaluate SS-31 binding isotherms based on measurements of zeta potential (Fig. 3B).

C. Nonlinear least squares fitting

Evaluation of binding data to simultaneously solve for n and K_{D} (Eq. S2 and S3), to solve for K_{P} (Eq. S9), and to evaluate membrane electrostatics (Eq. S10-S12) was performed as described (7) using *Wolfram Mathematica 10*. Note that some binding experiment series in this study included conditions in which binding isotherms did not reach saturation (e.g., Fig. 3A), rendering binding responses that could not, individually, be adequately fit by Eq. S2 or Eq. S3. In such cases, we performed a global analysis of all binding curves within a given experiment set, including those that did reach saturation, with the assumption that all binding curves will asymptote toward the same maximal value.

D. Analysis of MD simulations

From our simulations, we ascertained: (i) binding time-dependence and membrane insertion depths for each residue (Figures 5B and S11); (ii) binding poses of the peptide within the interfacial region (Figure 5C); (iii) radial distribution profiles of sidechain to lipid headgroup for each residue (Figures 5D and S12A) and between lipid headgroups (Figure 12C) for each system; (iv) co-diffusion of Arg and coordinated lipid phosphates (Figure S12B); (v) mean square displacement plots for each lipid with and without SS-31 and their corresponding diffusion coefficients (Figure S13); and (vi) mean solvent accessible surface area measurements of the acyl chain regions for each lipid with and without SS-31 (Figure S14).

The binding time-dependence, membrane insertion depth, and co-diffusion of Arg and coordinated lipid phosphates were analyzed using the MDTraj Python module (10) to process the trajectories and in-house Python scripts to conduct the analysis. The average and standard errors of the mean membrane insertion depths were calculated from selected residues (n=10 for Arg and Lys residues in all three lipid systems; n≥7 for 2′,6′-Dmt and Phe residues in all three lipid systems) which achieved stable positions within the bilayer and remained bound for the remainder of the trajectory. Radial

distribution profiles were generated using the GROMACS *gmx rdf* function (11,12). Mean square displacements were calculated using the *gmx* msd function in GROMACS (11,12) by tracking the phosphates of POPG and POPC and the central glycerol C2′ carbon of TOCL and MLCL. Area per lipid calculations were made using the GROMACS *gmx energy* function to extract the average x-y dimensions from three separate segments of the trajectory and dividing by the number of lipids per leaflet. Bilayer thicknesses were calculated using in-house scripts to determine differences in the average z-position of lipid phosphates in the upper and lower leaflets of each system. To calculate the average lipid phosphate z-positions, each lipid phosphate was treated as an independent sample to calculate mean z-positions over each trajectory. Solvent accessible surface area (SASA) measurements were calculated using the *gmx sasa* function in GROMACS (11,12), which uses the double cubic lattice method (13). For the SASA analyses, we defined the acyl chain region of a lipid as the carbon and hydrogens below the ester carbon. The upper leaflet of each bilayer that contained the SS-31 peptides was used for both the radial distribution profiles, the mean square displacement, and SASA analyses for the "with SS-31, upper leaflet" condition. The lower leaflet of each bilayer in "+SS-31" trajectories, but that was not in contact with peptides, was used for the diffusion coefficients and the SASA analyses for the "with SS-31, lower leaflet" condition. Both leaflets of the systems without any peptide were used for the mean square displacement analysis, radial distribution profiles (normalized for lipid count), and SASA analyses (normalized for lipid count) for the "without SS-31" condition. Diffusion coefficients were calculated from the mean square displacement analysis and fit to the linear portion of the curve (10 to 50 ns) using the Einstein relation:

$$\lim_{t\to\infty} \langle \| r_i(t) - r_i(0) \|^2 \rangle_{i\in A} = 6D_{\mathrm{A}}t$$

The average diffusion coefficients (Fig. S13B) and average acyl chain SASA measurements (Fig. S14) were calculated from the averages over three equal intervals of ~515 ns in the systems with peptide, and over three equal intervals of ~315 ns for the systems without peptide. 95% confidence intervals were calculated from the standard error of the averages from each interval of the respective trajectories. All images of the systems were created using VMD (14). All figures were created using the Matplotlib Python module (15).

Supporting Information - Results

A. Spectral characterization of SS-31 in solution (*pertaining to Figure S3*)

SS-31 contains two fluorescent side chains (Figure 1A): a phenylalanine (Phe) at position four and a 2′,6′- dimethyltyrosine (2′,6′-Dmt, tyrosine [Tyr] with methyl groups at the δ^1 and δ^2 positions of the aromatic ring) at position two. We selected the 2′,6′-Dmt moiety as a potential reporter for SS-31

membrane interactions because (i) Tyr has a significantly higher extinction coefficient and quantum yield than Phe, and (ii) fluorescence measurements can be designed to selectively measure Tyr fluorescence in the presence of Phe, because absorption and emission spectra of the two side chains are sufficiently separated (Phe absorbing and emitting at lower wavelengths) (16). The aromatic side chain of tyrosine contains a phenol chromophore with fluorescence properties that are sensitive to its microenvironment (17-19) and may be used to measure peptide and protein interactions with membranes (20-22).

Initially we characterized the spectral features of the 2′,6′-Dmt side chain of SS-31 in comparison with L-Tyr in aqueous buffer (Figure S3). We first measured absorbance and fluorescence spectra of L-Tyr and SS-31. In comparison with free tyrosine, SS-31 displayed a more structured and red-shifted absorbance with a concurrent shift in the wavelength of maximal fluorescence excitation ($\lambda_{ex}^{SS\text{-}31}$ = 281 to 283 nm) (Figure S3A, compare red and blue traces). We next measured the relationship between fluorescence yield and concentrations of L-Tyr or SS-31. The emission intensity of SS-31 linearly increased with concentration up to 100 μM (Figure S3B, red) and its emission was reduced in comparison with free L-Tyr (Figure S3B, blue). These steady-state measurements were consistent with shorter measured fluorescence lifetime of SS-31 in solution ($<\tau^{SS\text{-}31}>$ = 1.07 ns) in comparison with L-Tyr ($<\tau^{Tyr}>$ = 3.33 ns). To address the solvent accessibility of the 2′,6′-Dmt side chain of SS-31, we measured dynamic quenching of L-Tyr and SS-31 by the collisional quenching agent acrylamide. Stern-Volmer analysis showed a reduced solvent accessibility of the 2′,6′-Dmt in SS-31 ($K_{SV}^{SS\text{-}31}$ = 4.8 M^{-1}; k_q = 4.49 x 10^9 M^{-1} s^{-1}) in comparison with free L-Tyr (K_{SV}^{Tyr}= 17.1 M^{-1}; k_q = 5.14 x10^9 M^{-1} s^{-1}) (Figure S3C). Finally, to confirm that the Phe residue of SS-31 did not contribute to our measured signals, we compared emission spectra of SS-31 with SS-20, a peptide variant with two Phe residues (Figure 1B). Under the conditions used for our fluorescence measurements, SS-20 is spectrally silent (Figure S3D, note that the intensity of SS-20 emission overlaps with the abscissa of the graph).

Thus, taken together, the data of Figure S3 reveal the following: (i) compared with L-Tyr, the 2′,6′-Dmt side chain of SS-31 in solution is less solvent accessible, possibly due to steric shielding by neighboring side chains; (ii) in comparison with L -Tyr, SS-31 has a decreased fluorescence lifetime and emission intensity, likely due in part to proximity of the ε-NH_3 group of lysine, which is a strong quencher of phenolic fluorescence (23); and (iii) within μM concentrations, the intrinsic fluorescence emission of SS-31 is in the linear dynamic range.

B. Fluorescence-based analysis of SS-31 membrane interactions (*pertaining to Figure S4*)

We next addressed the ability of the 2′,6′-Dmt side chain of SS-31 to serve as a reporter for membrane interaction of the peptide (Figure S4). Membrane binding measurements of Figures S4A and S4B were performed by incubating 20 μM of SS-31 with large unilamellar vesicles (LUVs) in a low- or

high-salt buffer (buffer with no added salt or with 100 mM KCl added, respectively) for 20 min, followed by spectroscopic analysis. Binding of SS-31 to these model membranes is believed to be restricted to the external lipid leaflet in these studies. We therefore quantified added lipid as the effective lipid concentration of the outer leaflet (denoted as $[L]^{eff}$ or $[lipid]^{eff}$), which for LUVs of this size is half of the total lipid ($0.5x[L]^{tot}$) as described (24). In the experiments of Figures S4A and S4B, the $[L]^{eff}$ was 100 μM; hence, the peptide to effective lipid concentration ($[P]:[L]^{eff}$) was 1:5. For simplicity, we base all data analyses using this outer leaflet lipid concentration; in cases where the absolute total amount of lipid used, it will be reported as $[L]^{tot}$ or $[lipid]^{tot}$.

LUVs consisted of either pure POPC or 20 mol% anionic lipids (TOCL, MLCL, or POPG) in a host background of POPC. Emission scans of SS-31 in the absence and presence of LUVs revealed three important features (Figure S4A). First, although incubation of SS-31 with membranes did not cause a spectral shift in 2′,6′-Dmt fluorescence, it did effect an increase in emission intensity. We attribute this phenomenon to a transfer of the 2′,6′-Dmt moiety from the aqueous environment to a bilayer-inserted state. This is not likely due to a simple change in the dielectric of the side chain microenvironment, but rather to other factors that include: (i) a change in solvent hydrogen bonding with the phenolic hydroxyl group, (ii) a reversal of quenching by another side of the peptide (e.g., the lysine at position three), and (iii) specific polar interactions between 2′,6′-Dmt and lipid headgroups. Second, the magnitude of LUV-induced emission increase is lipid-dependent. As shown previously (25-28), SS-31 has a strong propensity to bind bilayers containing lipids with anionic headgroups, but binds very weakly to bilayers with a net zero charge. Our results show that the emission increase in the presence of LUVs containing only the zwitterionic POPC is very weak, and is much more robust in the presence of LUVs composed of 20 mol% anionic lipid. Further, binding to bilayers containing the dianionic TOCL and MLCL is significantly higher than it is to bilayers containing the monoanionic POPG. Finally, the LUV-induced emission increase is significantly reduced in the presence of high salt, particularly for bilayers with anionic lipids. This is explained by charge shielding of the anionic lipid headgroups by solution electrolytes, which decreases the interfacial surface charge (8,9). Together, these results show that: (i) the intrinsic emission of the 2′,6′-Dmt side chain of SS-31 increases with membrane binding, and (ii) that SS-31 interaction with lipid bilayers is sensitive to the bilayer surface charge density and to the presence of electrolytes in solution.

To compare the solvent accessibility of the 2′,6′-Dmt reporter when SS-31 is free in solution and when SS-31 is membrane-bound, we performed dynamic quenching experiments in the presence and absence of LUVs (Figure S4B). Note that due to the sensitivity of SS-31 membrane binding to the ionic strength of the solution, the use of a charged collisional quenching agent would have untoward effects on binding; we therefore used acrylamide (formal charge of zero) as a dynamic quencher in these

experiments. For this analysis, the measured fluorescence was corrected for the absorbance of exciting light by acrylamide as described (29). For SS-31 in the absence of membranes, the 2′,6′-Dmt moiety was solvent exposed, as expected, under low salt (K_{SV} = 5.7 M^{-1}) and high salt (K_{SV} = 5.4 M^{-1}) conditions. In the presence of LUVs under low salt conditions, acrylamide accessibility was almost negligible (K_{SV} = 0.15 M^{-1}, 0.02 M^{-1}, and 0.21 M^{-1} for LUVs containing 20% TOCL, MLCL, and POPG, respectively). The presence of high salt reduced the LUV-dependent protection from acrylamide for vesicles with 20% TOCL and MLCL (K_{SV} = 1.20 M^{-1} and 1.04 M^{-1}, respectively) and more so for vesicles with 20% POPG (K_{SV} = 2.19 M^{-1}). These results are consistent with the salt-sensitivity of membrane binding observed in our emission scans.

Reduction of the surface charge density of liposomes containing anionic lipids by the addition of cationic species can induce vesicle aggregation. To address whether the presence of peptides induced aggregation of our model membranes, we performed turbidity measurements over a range of peptide and lipid molar ratios (Figures S4C,D). Optical absorbance is an established means of monitoring changes in vesicle size (e.g., by flocculation, fusion, micellezation, etc.) due to soluble factors such as membrane-active peptides (30). First, we measured absorbance changes by titrating solutions with LUVs (20% TOCL, MLCL, or POPG) with increasing [SS-31] (Figure S4C). For comparison, we performed parallel experiments in which we titrated LUVs with divalent cations. Below a threshold [SS-31]:$[lipid]^{tot}$ ratio of 1:1 (or a $[Ca^{2+}]$:$[lipid]^{tot}$ ratio of 10:1), we observed no change in absorbance; above these thresholds, absorbance increases were observed within a narrow concentration range. These results are consistent with previous studies measuring Ca^{2+}-induced aggregation of cardiolipin-containing liposomes (31). Second, to account for any effects of lipid concentration, we measured the absorbance of solutions containing increasing concentrations of LUVs containing 20% TOCL in the absence or presence of SS-31 (Figure S4D). For this assay we analyzed a range of LUV concentrations (up to $[lipid]^{tot}$ = 250 μM) corresponding to the range of concentrations used in the majority of our SS-31 binding experiments. We also used a [SS-31]:$[lipid]^{tot}$ of 1:10, a peptide concentration approaching binding saturation for this lipid composition. Based on this assay, we did not observe peptide-induced aggregation at any LUV concentration.

Taken together, we conclude from the analyses of Figure S4 that: (i) when SS-31 is membrane-bound, the 2′,6′-Dmt side chain increases its emission yield, confirming that this is an excellent reporter for SS-31 bilayer interactions; (ii) the SS-31 bilayer interaction is strongly dependent on membrane surface charge and solution ionic strength; and (iii) over the range of [SS-31] and [LUV] typically used throughout this study, there is no observable aggregation of our model membranes.

C. Quantitative analysis of SS-31 membrane binding isotherms (*pertaining to Fig. S5*)

In Figure S5, we illustrate our approaches for fitting SS-31-bilayer binding measurements with simulated data sets. By one approach, SS-31 binding to lipid bilayers was analyzed as a Langmuir-type isotherm. For experiments performed by titrating a fixed concentration of lipid ($[L]_0$) with peptide ($[P]_0$), simulated binding curves (θ vs. $[P]_0$, fit with Eq. S2) originating from different n and K_D values are shown in Figure S5A. The total amount of bound peptide over each titration range ($[P]_b$ vs. $[P]_0$) for each associated case is shown in Figure S5B. By comparison, experiments performed by titrating a fixed concentration of peptide ($[P]_0$) with lipid ($[L]_0$) for the same n and K_D values produce binding curves (θ vs. $[L]_0$, fit with Eq. S3) shown in Figure S5D, with the total amount of bound peptide over each titration range shown in Figure S5E. Corresponding Scatchard plots for peptide (Eq. S6) and lipid (Eq. S8) titrations are shown in Figure S5, panels C and F, respectively.

D. SS-31 titration time course analysis and ITC (*pertaining to Figs. S6 to S8*).

SS-31 binding isotherms measured by peptide titration were generally conducted by time course measurements in which a solution of LUVs at a fixed lipid concentration was titrated with SS-31. Such experiments typically entailed total lipid concentrations up to 250 μM (i.e., up to $[L]^{eff}$ of 125 μM) with the progressive addition of SS-31 in 2 μM increments. This assay is based on the measurable increase in emission from the 2′,6′-Dmt side chain upon the transition from bulk aqueous solution to the lipid bilayer (Figure S4). To enhance the difference between emission intensity of the soluble vs. bound peptide, these assays included a fixed concentration of acrylamide in the aqueous medium (32).

Representative time courses of SS-31 titrations are shown in Figure S6. As expected, these traces show that the SS-31 emission jumps are high when lipid is available for binding, and become measurably reduced at a point during the titration when lipid binding sites become saturated. Beyond this point, fluorescence increases with further peptide addition are small and linear with added SS-31 because all added peptide either remains soluble or dynamically replaces pre-bound peptide.

The time course data from these measurements can be analyzed as shown in Figure S7. Plotting the stepwise emission intensities as a function of $[P]$:$[L]^{eff}$ and determining the best linear fits to pre- and post-binding site saturation phases of the titrations, the value of the abscissa at the intersection of the two lines corresponds to the $[P]$:$[L]^{eff}$ at saturation (i.e., $1/n$) (Figure S7 A,B). A summary of these analyses (Figure S7C) reveals the following. Under low salt conditions, SS-31 binds to LUVs with 20 mol% TOCL with an n value of ~5 (roughly one peptide bound per five lipid molecules, or one peptide per cardiolipin molecule on the external leaflet). For LUVs containing 10 mol% TOCL or 20 mol% POPG under low salt conditions, the n value roughly doubles to values of ~12 and 11, respectively. Together, these results suggest that the number of lipids that constitute an SS-31 "binding site" at saturation is

formally determined by the net charge at the bilayer surface (roughly one peptide per two lipid headgroup charges). This analysis also reveals the effect of ionic strength on the binding stoichiometry, as the *n* value for LUVs with 20 mol% TOCL increases to ~9 in the presence of high salt. Hence, the peptide-lipid stoichiometry is a function of surface charge density (σ), which is determined by both the intrinsic charge of the lipids and the concentration of electrolytes in the interfacial region.

Notably, the analysis of Figure S7 works well under high affinity interactions when added peptide essentially binds quantitatively to available lipid sites and the pre- and post-saturation regimes of the titrations can be easily delineated. However, under conditions in which the binding affinity is reduced (e.g., lower lipid headgroup charge and/or higher ionic strength), the difference between the two regimes becomes more difficult to delineate and binding parameters must be evaluated by curve fits using nonlinear least squares analysis (Figures 2 and 3).

Microcalorimetry experiments were conducted by the progressive addition of LUVs with different lipid composition (20% TOCL, MLCL, or POPG in a POPC background or 100% POPC as a control) at increments of 20 nmol total lipid (10 nmol effective lipid) to solutions of peptide. The starting concentrations of SS-31 in the sample cell were varied ([SS-31] = 175 μM for CL-containing membranes; [SS-31] = 87.5 μM for PG-containing membranes) so that a complete binding curve could be obtained over the injection course. Representative heat flow time courses corrected for heats of dilution (Figure S8) show that the peptide-bilayer interactions are exothermic and saturable. ITC data were analyzed as Wiseman plots (Figure 2D) to obtain K_A, *n*, ΔH and ΔS.

E. Model membrane surface electrostatics and SS-31 binding (*pertaining to Fig. S9*)

1. Effects of ionic strength on SS-31 binding

To address the salt-dependent reversibility of SS-31 membrane interactions, we measured the relative SS-31 membrane binding when increasing concentrations of salt were added before or after peptide was bound to membranes (Figure S9A). We found no difference in the ionic strength-dependent decrease in SS-31 bilayer interaction whether salt was added before or after peptide binding, quantified as either emission intensity (Figure S9A, *left*) or anisotropy (Figure S9A, *right*). To substantiate this observation, we performed kinetic measurements of relative SS-31 binding with progressive addition of monovalent or divalent cations (Figure S9B). The addition of salt caused rapid fluorescence-detected desorption of SS-31 from the bilayer surface that was commensurate with cation charge. We conclude that the binding of SS-31 to model membranes is highly reversible in a manner that is dependent on solution salt concentration. Hence, based on GCS models, cations "compete" with SS-31 for binding by two possible mechanisms: (i) electrostatic shielding, and (ii) specific complexation with lipid functional groups containing formal (phosphates) or partial (e.g., carbonyl oxygens) negative charges.

2. Effect of membrane binding on SS-31 formal charge

Our SS-31 binding analyses (Figure 2) suggest that when peptide is maximally bound to the surface of an anionic bilayer, there is a near balance of charge between peptide formal charge (+3) and headgroup phosphates (one peptide binds per every ~1.5 CL/MLCL molecules or per every ~3 PG molecules). However, our ζ profiles of Fig. 3C suggest that a measurable negative charge density exists among the headgroup phosphates even when SS-31 is maximally bound to membranes. This observation could originate from phenomena such as partial charge neutralization of SS-31 upon membrane binding (as found for other polybasic peptides, e.g., (33)) and/or changing conformations of ionized lipid headgroups that alter the electric potential sensed at the slip plane. To begin to address this question, we sought to determine the formal charge on bound SS-31 from the ζ profiles of Figure 3C, based on the additive charge densities of: ionized lipids (σ^{max}), bound SS-31 ($\sigma^{SS\text{-}31}$, taken from peptide titration binding isotherms (Figure 2A)), and adsorbed solution ions (σ^{ion}, based on K_M, the intrinsic association constant of ion complexation determined from LUVs in the absence of peptide). This analysis returned a formal charge on SS-31 of +1.7 and +2.3 for membranes containing 20% TOCL and POPG, respectively. Based on this analysis, bound SS-31 is approximately 30% charge-neutralized, which could, for example, result from the deionization of basic groups in the microenvironment of the bound state. Such partial charge neutralization of bound SS-31 could contribute to the persistence of negative surface potentials in model membranes even when peptide is maximally bound to the surface. Ongoing work in our group is aimed at using NMR- and electrokinetic-based approaches to address this question by examining the structural basis of the side chain-lipid headgroup interactions and the effect of peptide on headgroup orientation. We are also working toward developing computational binding analyses in conjunction with our MD analyses that extend traditional GCS models to: (i) replace "smeared charge" assumptions with "discreetness of charge" effects that could result in altered lateral organization of lipids upon peptide binding; (ii) account for atomic details of the peptide and membrane; and (iii) include Born repulsion (image charge) effects in electrostatic interaction models (34,35).

F. Assays for peptide-induced structural polymorphism of model membranes (*pertaining to Fig. S10*).

1. SAXS measurements with model membranes

SAXS measurements of lipid samples yield x-ray diffraction patterns that convey information about polymorphic state and bilayer dimensions (36). SAXS profiles for LUVs of different lipid composition in the presence of different molar ratios of SS-31:lipid are shown in Figure S10A. These plots show scattering intensity $I(q)$ as a function of momentum transfer q, where $q = (4\pi/\lambda) \sin\theta$, λ is the wavelength of the x-ray beam, and θ is half of the angle between the incident beam and scattered

radiation. Scattering profiles of our LUVs containing anionic lipid show a broad peak between q = ~0.05 and 0.25 Å^{-1}, typical of unilamellar vesicles. By contrast, profiles of LUVs containing POPC only (not shown) display a superposition of this broad peak and two peaks corresponding to first- and second-order Bragg reflections (with q spacings of n2π/d, where n is the peak order and d is the repeat distance accounting for the bilayer and intervening water), suggesting the presence of some oligolamellar vesicles.

We fit our SAXS data using the Global Analysis Program (GAP) (37,38), kindly provided by Dr. Georg Pabst (University of Graz). Fitting was performed using the equation

$$I(q) = \frac{(1 - N_{\text{diff}})S(q)[F(q)]^2 + N_{\text{diff}}[F(q)]^2}{q^2} \qquad \textit{Eq. S13}$$

where N_{diff} is the fraction number of uncorrelated bilayers per scattering domain, $F(q)$ is the bilayer form factor, and $S(q)$ is the interbilayer structure factor based on Caillé theory. From these fits, the following parameters are shown beneath each corresponding plot: d (the lamellar repeat distance), N_{diff}, z_H (the Gaussian distribution center of the polar heads), and η (the fluctuation parameter related to bilayer bending rigidity). These values show no systematic trends that correlate with different molar amounts of SS-31. Hence, we conclude that SS-31 caused no SAXS-detected changes in membrane structure.

2. ^{31}P ssNMR measurements with model membranes

Phosphorous NMR allows for the analysis of lipid phosphate headgroups, yielding specific spectral lineshapes characteristic of gel or liquid crystalline lamellar, inverted H_{II} and cubic phases (39). As a complement to our SAXS analyses, we used ^{31}P solid state NMR to test for any peptide-induced changes in our model membranes. The major phospholipids used throughout this work all have main transition temperatures below freezing (T_m^{POPC} = -2ºC; T_m^{TOCL} = -8.4ºC; T_m^{POPG} = -2ºC) (40-42). Therefore, at the temperatures used throughout this study, LUVs prepared as binary mixtures of these miscible lipids are expected to yield ^{31}P NMR spectra with powder patterns consistent with the liquid crystalline (Lα) phase.

To characterize the ^{31}P NMR spectra of the LUV samples used in this study, we hydrated a lipid film (20 mol% TOCL, 80 mol% POPC) in aqueous buffer and processed the dispersion in three ways: (i) preparation of multilamellar vesicles (MLVs); (ii) processing of MLVs by four freeze/thaw (liquid N_2 / 35ºC water bath) cycles; and (iii) extrusion through 0.1 μm polycarbonate membrane to produce LUVs. Analysis of these samples by ^{31}P ssNMR (Figure S10B) revealed that MLVs produced asymmetric lineshapes (low-field shoulder and high-field peak, CSA 54.4 ppm) characteristic of axially symmetric phospholipid in a lamellar phase. By comparison, MLVs subject to freeze/thaw cycles show some evidence of motional averaging (CSA 55.0 ppm), and samples that were extruded to produce unilamellar

vesicles of uniform diameter (LUVs, ~100 nm) showed complete averaging of the NMR spectrum, yielding a single isotropic peak. This observation is consistent with greater rotational mobility/tumbling of the smaller and more homogeneous LUVs compared with larger MLVs with stacked lamellae (43). We conclude that the LUVs used with SS-31 titration (Figure 4B) are indeed ~100 nm lamellar vesicles, although their size causes partial motional averaging of their spectral lineshapes.

3. Membrane-bound fluorescent probes

DPH is a rod-shaped fluorophore that partitions near the center of lipid bilayers and serves as an anisotropy probe for lipid dynamics and order due to the sensitivity of its rotational diffusivity to the microviscosity of its surroundings (44) (Figure S10C). The steady-state anisotropy of DPH ($\langle r \rangle^{DPH}$) increases with microviscosity (i.e., with decreased fluid dynamics of the hydrocarbon tails) and can report changes in membrane phase state, as occurs with thermotropic transitions between lamellar gel (Lβ) and liquid crystalline (Lα) phases. DMPC and TMCL are short acyl chain (14:0) variants of PC and CL whose Lβ to Lα phase transition temperatures (Tm) are within an experimentally tractable range (T_m^{DMPC} = 24°C and T_m^{TMCL} = 47°C). LUVs composed of a binary mixture of 20% TMCL and 80% DMPC and containing DPH yielded temperature-dependent maximum and minimum $\langle r \rangle^{DPH}$ values of 0.265 and 0.084 (corresponding to Lβ and Lα phases, respectively) with a sharp transition in $\langle r^{DPH} \rangle$ centered at 28.5°C, corresponding to a cooperative Lβ to Lα phase change (Figure S10D). Having established the range of $\langle r \rangle^{DPH}$ expected of this PC/CL system, we then used DPH anisotropy measurements to address the SS-31 response of binary lipid systems of longer chain and more unsaturated lipids used throughout this study (20% TOCL or 20% POPG in a host background of POPC). With increasing SS-31, we observed low $\langle r \rangle^{DPH}$ values that corresponded to the Lα phase for both TOCL- and POPG-containing membranes, with no measurable effect of peptide, even at saturating concentrations (Figure S10E). We conclude that SS-31 binding does not affect the rotational diffusion of the DPH probe, consistent with the binding of the peptide in the headgroup region with negligible effect on hydrocarbon chain dynamics.

Laurdan and prodan are interface-localized probes that contain the same naphthalene-based solvatochromic fluorescent moiety, but partition at different depths within in membrane due to differences in their nonpolar substituents: prodan (with a propionyl tail) resides near the level of phospholipid glycerol groups, whereas larudan (with a lauryl tail) resides deeper, near the level of the *sn*-1 carbonyl (45) (Figure S10C). Emission scans of laurdan- and prodan-containing LUVs composed of 20 mol% TOCL or POPG in a POPC background in the presence of different SS-31:$[Lip]^{eff}$ ratios are shown in Figure S10F. TOCL-containing membranes revealed modest, but consistent, peptide-dependent and saturable changes in the spectra of the probes. Specifically, laurdan fluorescence showed an SS-31-dependent decrease in I_{490} vs. I_{440} (equivalent to a blueshift), causing an increase in GP^{LAU}; prodan fluorescence showed an SS-31-dependent decrease in I_{480} and I_{520} relative to I_{420}, causing an increase in

3wGP^{PRO}. These changes correlate with decreases in probe hydration (reduced probe dipolar relaxation), indicative of higher order/lipid packing in the interfacial region (46-48). Measurable peptide-dependent increases in GP^{LAU} and in 3wGP^{PRO} were also observed for POPG-containing membranes, but were much reduced in comparison with TOCL-containing bilayers. Note that due to differences in the SS-31 binding capacities of POPG- and TOCL-containing bilayers (Figure 2), the titration range of [SS-31] for POPG bilayers was half of that for TOCL bilayers in these experiments.

G. Molecular dynamics analyses (*pertaining to Figs. S11-S14*).

1. Characterization of general membrane structural parameters

For our simulated membrane systems, the average area per lipid for bilayers of different composition were (mean ± SE): 20% TOCL, 75.49 Å^2 ± 0.04; 20% MLCL, 71.91 Å^2 ± 0.01; and 20% POPG, 64.03 Å^2 ± 0.11. Bilayer thickness was calculated as the phosphate-to-phosphate (P-P) distance between leaflets in the presence and absence of SS-31 in the upper leaflet. Average P-P distances were (mean ± SE): 20% TOCL (no SS-31), 3.98 nm ± 0.04; 20% TOCL (with SS-31), 3.95 nm ± 0.04; 20% MLCL (no SS-31), 3.90 nm ± 0.04); 20% MLCL (with SS-31), 3.87 nm ± 0.04; 20% POPG (no SS-31), 3.90 nm ± 0.04; 20% POPG (with SS-31), 3.87 nm ± 0.05. These measures of area per lipid and bilayer thickness are consistent with MD work from our group (49,50) and others (e.g., (51)). To complement these P-P distances that were calculated over the bilayer ensemble, we also calculated distances between upper leaflet phosphates and the bilayer COM (phosphate z-positions) for individual lipids (Figure S11A). Given the acyl chain asymmetry of MLCL, we refer to the headgroup phosphate on the side of the lipid with the full acyl chain complement as "P1" and to the phosphate on the side lacking an *sn*-2 chain as "P3" (corresponding to the "native " and "lyso" phosphates, P_N and P_L, respectively in (50)). Although the headgroup phosphates of TOCL are equivalent, we distinguish them as "P1" and "P3", corresponding to the side from which the acyl chain was cleaved in generating MLCL from TOCL. As expected, these phosphate z-positions are roughly half the distances of our calculated P-P bilayer thicknesses. Notably, however, the presence of SS-31 caused a slight but consistent reduction in P-P bilayer thickness, as well as reduction in the z-position specifically of anionic lipid phosphates (Figure S11A). We discuss the potential origin of this peptide-dependent effect on phosphate distances below.

2. Measurement of side chain insertion depth

In each MD binding trajectory (Figure S11B), basic side chains revealed rapid membrane association with z-coordinates that remained generally consistent from the point of membrane binding until the end of the simulation. By comparison, the 2′,6′-Dmt side chains, and to a greater extent the Phe side chains, displayed rapid transitions between the water- and nonpolar core-exposed states until

becoming stably bound in the acyl chain region. Residue-specific membrane insertion depths (Figure S11C) were determined for bound peptides, which were defined as those having both aromatic groups buried in the acyl chain region. For each bound peptide in the three different bilayer systems, we quantified the average z-coordinate of each side chain (z^{pos}) based on landmark atoms (the D-Arg Cζ atom; the 2′,6′-Dmt Oη atom; the Lys Nζ atom; or the Phe Cζ atom). Specifically, the average and standard errors of the mean membrane insertion depths were calculated from selected residues ($n = 10$ for Arg and Lys residues and $n \geq 7$ for 2′,6′-Dmt and Phe residues in all three lipid systems) that achieved stable positions within the bilayer and remained bound throughout the trajectory. Position variability was quantified as the 95% confidence intervals of z^{pos} values for each side chain. We observed no significant side-chain specific differences in penetration depth among the three lipid systems.

3. Radial distribution profiles: lipid distributions around SS-31 residues

The proximity of different lipid headgroups around each side chain landmark atom (defined above) was measured by lateral (x-y plane) radial distribution profiles (Figure S12A). For basic side chains, we observed three distinct radial shells of phosphate groups: a primary density located ~4Å from the side chain atom, as well as secondary and tertiary densities located ~6Å and ~9-10Å from the side chain atom, respectively. The primary peak, consistent with the Arg-phosphate and Lys-phosphate hydrogen bonding distances (52), shows two features for TOCL-containing membranes. First, both basic side chains show a strong preference for TOCL phosphates over POPC phosphates in this primary shell, consistent with the greater accessibility of the charged phosphate group in the former (53). Second, the peaks are higher for Arg than they are for Lys, consistent the more extensive hydrogen bonding contacts possible by the guanidino group relative to the primary amine, as also seen with MD simulations of penetratin (54). Compared with TOCL, the magnitude of the primary phosphate shell around Arg and Lys is reduced for POPG, but the general trends are similar. Interestingly, however, for MLCL-containing bilayers, the primary phosphate shell around Arg is not significantly enriched in MLCL phosphates over POPC phosphates. This is likely attributable, in part, to disruption of intermolecular phosphate contacts due to hydrogen bond contacts with the hydroxyl at the *sn*-2 position of the lyso side of MLCL (50). We also note that this reduction in Arg-phosphate contacts in MLCL-containing bilayers appears to be compensated by enhanced contacts between Lys and MLCL phosphates. The secondary and tertiary phosphate shells likely consist primarily of: (i) phosphates on the same headgroup as the phosphate in the primary density (for di-phosphate TOCL and MLCL) and/or (ii) phosphates corresponding to those in the primary density of the alternate basic residue of a given peptide. As a complement to this analysis, co-diffusion between Arg and lipid phosphates from six different peptides in a 100 ns block of a given simulation (Figure S12B) supports preferential interaction between this basic side chain and TOCL

phosphates. The hydrogen bond network that stabilizes the interaction between the Arg guanidino group and phosphates illustrates the potential for Arg to mediate interaction with multiple lipid headgroup phosphates (55).

4. Effects of SS-31 on lipid bilayer properties

Lipid-to-lipid radial distribution profiles (based on phosphate-phosphate radii) revealed two primary peaks in the absence of peptide, corresponding to successive lipid shells centered around 6Å and 9Å, respectively (Figure S12C, *black traces*). In comparison with this radial distribution based on lipid self-association without peptide, the presence of SS-31 caused a slight but measurable enhancement in the local concentration of anionic lipid (Figure S12C, *red traces*). This effect most likely originates from localization of anionic lipids in the vicinity of bound peptide by specific interactions with basic side chains (Figure S12A,B). We also note that the observed SS-31-dependent decrease in the average z-positions of anionic (but not zwitterionic) lipid phosphates (Figure S11A) is likely attributable, at least in part, to the polar interactions that these phosphates make with the basic side chains of the peptide. That is, the presence of peptide causes anionic lipid phosphates to reside deeper in the bilayer (by ca. 0.4-0.5Å), perhaps reflecting a balance between maintaining the position of the headgroup within the aqueous interface and the interaction between the phosphate and the basic side chain.

Based on the lateral mean square displacement of lipid phosphates, we observed marked SS-31-dependent decreases in the diffusivity (D_{xy}) of all tested lipids (Figure S13). Specifically, the presence of SS-31 caused a decrease in D^{TOCL} from 0.52 x 10^{-7} cm^2 s^{-1} to 0. 34 x 10^{-7} cm^2 s^{-1}, a decrease in D^{MLCL} from 0.53 x 10^{-7} cm^2 s^{-1} to 0.35 x 10^{-7} cm^2 s^{-1}, and a decrease in D^{POPG} from 0.74 x 10^{-7} cm^2 s^{-1} to 0. 48 x 10^{-7} cm^2 s^{-1}. The presence of peptide also caused a decrease in D^{POPC} for all systems tested. Furthermore, we compared the diffusivity of lipids between upper (peptide-accessible) and lower (peptide non-accessible) leaflets in simulations containing SS-31. Although the lower leaflet displayed trends toward SS-31-dependent decrease in D_{xy} in these simulations, they did not significantly differ from the no-peptide simulations. Therefore, we conclude from this analysis that the binding of SS-31 causes a marked reduction in lipid lateral diffusion on the leaflet to which peptide is bound.

Our analysis of the solvent accessible surface area (SASA) of acyl chains in our simulations (Figure S14) revealed two key features. First, using increased SASA as a measure of disturbance in lipid packing (acyl chain exposure), we found that the presence of TOCL caused greater local packing defects than did MLCL, and that both CL variants caused greater packing defects than POPG. These results, consistent with our previous work (50), are attributable to the molecular geometries of these anionic lipids (TOCL, and to a lesser extent, MLCL, have lower effective headgroup volumes relative to acyl chain volume occupancy, than does POPG). Second, this analysis showed peptide-dependent decreases in

solvent accessibility of all tested lipids; specifically, $SASA^{TOCL}$ decreased from 12.0 nm^2 to 9.8 nm^2, $SASA^{MLCL}$ decreased from 9.4 nm^2 to 7.8 nm^2, and $SASA^{POPG}$ decreased from 5.8 nm^2 to 4.7 nm^2. Consistent with our analyses of lipid diffusion, SASA was only measurably decreased on the leaflet to which SS-31 was bound. These results suggest that the binding of SS-31 has the effect of reducing the accessibility of solvent (and solvated ions) to acyl chains that would otherwise be exposed to the interface, largely by lipid packing defects.

H. Assays for measuring Ca^{2+} interactions with model membranes and mitochondria (*pertaining to Figs. S17-S19*).

1. Modeling of SS peptide effects on ψ_x decay and ion distribution

Profiles of distance-dependent potential decay and ion distribution based on GCS theory shown in Figure 7A were determined by: (i) assuming a lipid bilayer composed of 20% CL in a host background of zwitterionic lipid (2*e* per five lipids, $\sigma \sim -4.9\text{x}10^{-3}\ e\ \text{Å}^{-1} = -7.8\text{x}10^{-2}\ C\ \text{m}^{-2}$); (ii) using bulk ionic concentrations of monovalent 1:1 electrolyte (e.g., K^+Cl^-) of 80 mM and 2:1 electrolyte (e.g., $Ca^{2+}Cl^-_2$) of 1 μM; and (iii) using a bulk concentration of SS-31 (where present) of 100 nM, wherein peptide is modeled as a trivalent ion.

2. Characterization of fluorescence-based analyses of calcium dynamics and $\Delta\Psi_m$

CG-5N is a membrane-impermeant probe whose fluorescence intensity increases upon binding calcium. To properly evaluate the responses of CG-5N under the different experimental conditions used in this study, we first measured SS peptide-dependent changes in probe emission under conditions used for experiments with model membranes (Figure 7B) and under conditions used for experiments with isolated mitochondria (Figure 7C,D). In these control experiments, calcium and SS peptides were added in concentrations commensurate with the respective assays of Figure 7.

Given the potential for some peptides to bind metal ions (56), we first addressed the potential effects of SS-20 and SS-31 on CG-5N fluorescence. In the minimal buffer used for the measurement of model membranes supplemented with calcium, the addition of SS-20 caused a nearly negligible decrease in CG-5N emission in comparison with dilution effects of vehicle-only titration, whereas the addition of SS-31 caused a slightly greater decrease in CG-5N emission (Figure S17A). Notably, however, the quenching effect of both peptides was significantly less than that of the metal ion chelator EDTA, which in these measurements was added in molar amounts 2.5x lower than peptide. Our characterization of the ability of SS peptides to decrease the accumulation of Ca^{2+} ions within the interface of anionic membranes under equilibrium conditions is shown in Figure 7B and in Figures S17B,C. The main conclusion from these analyses is that SS peptides bind to anionic lipid bilayers in the headgroup region,

thereby shifting the equilibrium binding of Ca^{2+} ions away from coordination at the membrane and toward bulk solution. This conclusion is predicated on the assumptions that CG-5N accurately reports [Ca^{2+}] in the bulk solution and that SS peptides do not significantly influence bulk [Ca^{2+}] in a manner other than by competing for Ca^{2+} binding at the bilayer surface. Our control tests shown in Figure S17A do indicate that, under these experimental conditions, SS peptides (SS-31 in particular) may have slight effects on CG-5N spectral properties (e.g., through a dynamic quenching mechanism) and on the bulk [Ca^{2+}]. However, they do not counter the conclusions from Figures 7B or S17B,C for the following reasons: (i) If SS peptides have a slight quenching effect on CG-5N fluorescence, this effect is *opposite* to what is observed in Figures 7B and S17C (that peptides show an increase CG-5N emission due to increased bulk [Ca^{2+}]); hence, any untoward quenching effect is likely negligible under these conditions; (ii) If SS-31 has the effect of chelating bulk phase Ca^{2+} ions that is significant under our experimental conditions, the responses in Figures 7B and S17C would be different between SS-31 and SS-20; the fact that they are nearly identical indicates that any putative chelation behavior by SS-31 is very unlikely to have an influence on these results.

The buffer used for calcium measurements with mitochondria (20 mM HEPES, 300 mM sucrose, 2 mM potassium phosphate, 0.05% BSA, pH 7.5, supplemented with variable concentrations of $CaCl_2$) was adapted from a recent study on Ca^{2+} uptake into yeast mitochondria (57). By comparing CG-5N emission intensity in CFB buffer with and without BSA, we observed that the presence of BSA caused a robust (10.7-fold) reduction in CG-5N fluorescence. We attribute this to the ability of albumin to bind Ca^{2+} (as well as other metal) ions (58). We evaluated the response of the CG-5N probe to reagents used in our mitochondrial calcium transient measurements (Figure S17D). The most notable results of these time traces are as follows. First, vehicle-only controls for additions of respiratory substrate NADH and calcium ionophore ETH-129 had minor effects on CG-5N emission in the absence (sample 1) and presence (sample 2) of mitochondria. Second, the presence of NADH itself increased CG-5N emission (samples 3 and 5). We attribute this NADH-dependent increase in CG-5N emission to the presence of Ca^{2+} in stock concentrations of NADH obtained from the vendor (Sigma N8129) because the presence of EDTA suppressed this NADH-dependent fluorescence increase (not shown). Importantly, this effect of respiratory substrate on CG-5N emission did not confound the analysis of time courses and calcium transients of Figure 7C or S18A,B because: (i) all samples were given identical concentrations of NADH, and (ii) all peptide treatments were performed prior to spectral analysis and fluorescence normalization allowed for direct comparison of all samples. Third, because *S. cerevisiae* mitochondria lack a calcium uniporter, in the absence of ETH-129 (sample 3), the addition of calcium resulted in an increase in CG-5N emission that was sustained over time (i.e., calcium remained in the bulk phase). This is consistent with the absence of dynamic changes in TMRM and CG-5N emission in samples lacking ETH-129 (see

Figure S18A, “no ETH-129”), confirming that these spectral changes did indeed reflect Ca^{2+} uptake. Fourth, when mitochondria were provided respiratory substrate (NADH) to generate a membrane potential, addition of ETH-129 resulted in a decay of CG-5N emission (sample 5). This was due to the membrane potential-dependent uptake of calcium that was present in the buffer system containing isolated mitochondria. Also in sample 5, the addition of calcium results in a transient response in which the [Ca^{2+}] of the bulk solution immediately increases, followed by the electrophoretic uptake into the matrix, manifest as a return of CG-5N emission to the base value. The latter shows the complete uptake of solution calcium into energized mitochondria.

TMRM is a potentiometric probe that is used to measure relative transmembrane potential ($\Delta\Psi_m$) of isolated mitochondria. Under the conditions used in this study, TMRM was analyzed in quench mode such that upon establishment of the $\Delta\Psi_m$, this lipophilic cationic dye accumulates in mitochondria in accordance with a Nernstian distribution and emission intensity decreases. The response of TMRM to $\Delta\Psi_m$ is shown in Figure S17E: addition of respiratory substrate caused a decrease in TMRM emission as the membrane potential was established, and dissipation of the $\Delta\Psi_m$ by the K^+ ionophore valinomycin caused an increase in the TMRM signal. Under our measurement conditions, the presence of SS peptide did not measurably affect the magnitude of the steady-state $\Delta\Psi_m$ established in isolated mitochondria.

3. NMR- and polarography-based analyses of mitochondrial calcium stress

Our protocols to evaluate the ^{31}P NMR spectra of isolated mitochondria were adapted from previous studies (59-62). Isolated mitochondria were suspended at high concentration (70 mg ml^{-1}) in a buffer compatible with NMR measurements (600 mM sorbitol; 20 mM HEPES-KOH, pH 7.5, consistent with that used in (62)). For each sample, NMR measurements (512 total transients) were taken over 26 min with temperature maintained at 4°C. We first compared the ^{31}P spectra of isolated mitochondria and hydrated dispersions of mitochondria-extracted lipids (Figure S19A). The lineshape of mitochondrial lipids revealed a powder pattern consistent with a lamellar bilayer of lipids with axial symmetry, with a CSA span of 31 ppm and a high-field peak at -12 ppm. The lineshape of our mitochondrial samples revealed multiple peaks, including an isotropic signal (“Iso”) and three distinct high-field peaks (“L1-L3”). The large isotropic peak likely originates from molecules undergoing fast reorientation on the NMR timescale, including NTPs, inorganic phosphate, and soluble phosphoproteins. The mitochondrial L2 peak coincides with the peak from our extracted lipids, suggesting that this peak corresponds to a lipid bilayer signal. Mitochondrial peak L3, shifted ~10 ppm upfield from the L2 lamellar component, may correspond to motionally-restricted lipids associated with membrane proteins.

We used NMR analysis as a means of measuring the ability of SS-31 to mitigate stress caused by high levels of calcium. To this end, our highly concentrated samples of mitochondria isolated from WT *S.*

cerevisiae were pre-incubated with or without 600 μM SS-31 on ice for 1h. This corresponds to a peptide concentration of 8.5 nmol SS-31 per mg mitochondrial protein, which is comparable to previous studies (e.g., (63)) in which 10-100 μM SS-31 was used with isolated mitochondria at ~0.4 mg ml^{-1} (25 to 250 nmol SS-31 per mg mitochondrial protein). Samples were then incubated with vehicle only or up to 5 mM $CaCl_2$ (i.e., up to ~700 nmol Ca^{2+} per mg mitochondrial protein) followed by NMR analysis. Compared with control samples that did not receive SS-31 or $CaCl_2$ treatment, incubation with SS-31 did not cause a major disruption of the ^{31}P spectra, although we did observe a slight shift of the L2 peak to a more shielded position (~1.0 ppm) in the presence of SS-31, whereas the other peaks did not change (Figure S19B). Addition of $CaCl_2$ resulted in the complete loss of the L3 component of our spectra, regardless of the presence of SS-31 (compare Figure S19B and Figure 7E). However, SS-31 did offset the calcium-dependent decay of the L2 component (Figure 7E,F).

Parallel measurements of mitochondrial oxygen consumption were based on conditions similar to our NMR measurements, but with the following differences. First, mitochondria received higher concentrations of SS-31 and $CaCl_2$ (54.4 nmol SS-31 and 1 μmol Ca^{2+} per mg mitochondrial protein, respectively). Second, respirometry measurements were made in a buffer MRB (Minimal Respiration Buffer) with reduced osmoticum (300 mM sucrose) and supplemented with 1 mg ml^{-1} BSA and 10 mM KH_2PO_4, pH 7.5. Oxygen consumption rates in this buffer and in the absence of calcium stress were higher than normal (~280 nmol O_2 min^{-1} mg^{-1}). To confirm that this elevated rate was due to the buffer conditions and not our isolated mitochondria, parallel respirometry measurements made in our Standard Respiration Buffer (SRB) showed reduced state 2 oxygen consumption rate (~190 nmol O_2 min^{-1} mg^{-1}) (Figure S19C).

Supporting Information - Discussion

A. Lipid determinants of SS peptide binding

In this study, the lipid determinants of SS-31 membrane interactions were determined based on empirical binding isotherms with model membranes (Figures 2 and S15) and MD simulations (Figures 5 and S11). Consistent with previous results (25,27), we found that SS-31 had negligible interaction with membranes composed solely of zwitterionic lipids, and required anionic lipids for saturable binding. Independent lines of evidence in this study support that the primary determinant of SS-31 binding is the number of negative charges within the lipid headgroup region. First, the binding of SS-31 to membranes containing tetra-acyl CL (TOCL) or its lysolipid variant (MLCL) was virtually indistinguishable, as demonstrated in model membranes (Figure 2) and isolated mitochondria (Figure 6A, WT *vs.* Δ*taz1*). Notably, this supports the ability of SS-31 to act as a therapeutic for the treatment of Barth syndrome, which is characterized by an increase in the MLCL/CL ratio of the MIM (64). Second, membranes

containing monoanionic PG supported roughly half of the SS-31 binding capacity observed in membranes with equal concentrations of CL (Figure 2). In this regard, mitochondria lacking CL biosynthesis sustained near-WT levels of peptide binding because PG was increased to roughly twice the normal CL concentration (Figure 6A, WT vs. Δ*crd1*). Together, these results support a model in which SS peptide binding to lipid bilayers depends on the anionic surface charge density, not the identity of a particular component lipid. Indeed, it has long been observed that nonspecific electrostatic attraction occurs between basic peptides and anionic lipid bilayers regardless of the chemical nature of either group (e.g., (65)). This model is supported by our atomistic MD work, showing that bilayer-docked SS-31 resides stably in the interfacial region with similar residue-specific penetration depths and lipid interactions among TOCL, MLCL, and POPG-containing bilayers (Figures 5, S11, and S12). Yet based on ITC measurements, the noncovalent interactions that mediate SS-31 binding appear to be dominated by entropy rather than by the energy of polar contacts (Figure 2D,E), underscoring the importance of nonpolar interactions between aromatic side chains of the peptide and the acyl chain region in determining binding stability. Therefore, a key role of the bilayer surface charge is likely the establishment of a strong electric field (see Figure S2) that increases the effective concentration of peptides in the interfacial region, where they are then able to establish binding interactions, which are dominated by hydrophobic contacts (Figure 8A). We note that although CL is not strictly required for SS-31 binding, it is likely essential for peptide action within mitochondrial membranes for two main reasons. First, under conditions of normal lipid metabolism, CL is the lipid that contributes the most to the strong negative surface charge of the MIM. As shown by our lipidomics data (Figure 6A), the concentration of dianionic CL is greater than that of other monoanionic phospholipids (PS, PA, PG); other highly charged lipids including phosphoinositides (the phosphorylated variants of PI) are generally confined to cytoplasmic leaflets of the endomembrane system (66) and are thus not abundant in mitochondria. Second, the inverted conical molecular geometry of CL, promoted by small headgroup volume and highly unsaturated fatty acids (cf. Figure S16A), creates packing defects in CL-rich bilayers. One manifestation of this packing defect is increased SASA of acyl chains (Figure S14), which may be critical for providing peptide side chain access to the nonpolar domains of membranes. As a corollary, specific physicochemical features of CL may be essential for downstream (post-binding) effects of SS peptides on membrane properties (see below). Hence, considering that polypeptide binding to lipid bilayers is determined by a combination of surface charge density and packing defects (67), we propose that SS peptides target the specialized environment of the MIM through a combination of a strong surface electrostatics and lipid packing defects imparted by features such as high [CL], highly unsaturated acyl chains, and low sterol content.

B. SS-31 equilibrium binding parameters

The equilibrium membrane binding parameters of SS-31 measured in this study elucidate aspects of the molecular MoA of these compounds. First, the lipid:peptide stoichiometry (n) serves as an index for peptide binding density (n scales inversely with the number of peptides bound per unit membrane area). We show that under conditions of maximal peptide binding density, model membranes composed of 20 mol% CL and 20 mol% PG bind SS-31 with $n \approx$ 6-7 (area occupancy ~500-600 Å^2) and $n \approx 15$ (area occupancy ~ 1000 Å^2), respectively. To put these values into context, consider a typical cell with a total mitochondrial volume of $2x10^3$ μm^3 and a total MIM surface area of $2x10^5$ μm^2 (68). Assuming, conservatively, that 10% of the MIM surface is available for peptide binding, this translates into a mitochondrial SS-31 concentration of 400 μM at maximal binding density. This estimate, although highly approximate, is consistent with the observed 1000 to 5000-fold mitochondrial accumulation in cells incubated with low nM amounts of peptide (63). Second, the binding affinity (K_D) of SS-31 for membranes was found to be in the low μM range. Compared with typical drug compounds that bind molecular targets with much higher affinity (e.g., K_D in the nM and lower range), the binding affinity of SS peptides to membranes is very weak. This low affinity range is to be expected if the primary mode of peptide interaction is with the thermally disordered lipid bilayer rather than with a molecular binding pocket with a defined covalent structure. Furthermore, the low μM affinity of SS-31 for membranes is consistent the pharmacokinetic profile of SS peptides, showing rapid clearance of the peptide after administration (28) as well as peptide release from cultured cells upon media exchange (69). Notably, the correspondence between SS-31 binding parameters with model membranes and with isolated mitochondria observed in this study supports a model in which the primary mode of SS peptide interaction is with lipid bilayers (Figure 6B). However, it remains possible (and perhaps likely) that a fraction of SS peptides may interact with proteins (e.g., within acidic pockets) and/or at protein-lipid interfaces, particularly given the protein-rich nature of mitochondrial membranes.

C. Effects of SS peptides on membrane physical properties

This study addressed the effects of SS peptides on membrane physical properties. Using ^{31}P NMR and SAXS, we found that SS-31 did not disturb the lamellar bilayers of model LUVs, even at high peptide binding densities (Figure 4A,B). This is consistent with previous work (63) and the present study (Figure S17E), showing that SS peptides, even at high concentrations, do not dissipate ion gradients ($\Delta\Psi_m$) of respiring mitochondria. As noted above, given their amphipathic character, SS peptides resemble AMPs. This shared amphipathicity likely drives the interactions with bacterial membranes (AMPs) and mitochondrial membranes of bacterial origin (SS peptides). We propose that the short (four-residue) length of SS peptides is the predominant feature that keeps them from disrupting membrane

structure, unlike AMPs, which are significantly longer (70). SS-31 did, however, affect properties of the interfacial membrane region in a dose-dependent manner. First, based on membrane-bound solvatochromic probes, SS-31 caused a modest but repeatable increase in lipid packing, particularly for CL-containing bilayers (Figure 4C), a result consistent with our analysis of peptide-dependent reduction in acyl chain solvent accessible surface area (Figure S14). Due to its conical molecular geometry, CL can cause localized exposure of acyl chains to the aqueous interface (50). Intercalation of SS-31 aromatic chains into these pockets could fill such interfacial "voids" (packing defects), which could in turn have implications for bilayer properties such as mechanostability and elasticity. Second, based on computational approaches, we found that SS-31 caused a reduction in the lateral diffusivity of lipids (Figure S13), consistent with a model in which peptide binds tightly enough to anionic lipids that they co-diffuse for significant (at least μsec) time scales. Third, using electrokinetic and fluorescence-based approaches, we found that SS-31 decreases the surface charge (and hence the ψ_s) of both model (Figure 3B,C) and mitochondrial (Figure 6C) membranes. All of these observed effects are likely interrelated. For example, by partially neutralizing the charge of anionic lipids, the binding of SS-31 will reduce the hydration and effective cross-sectional area of headgroups, as well as decrease inter-lipid electrostatic repulsion, all of which could increase lipid packing. We therefore propose a model for the molecular MoA of SS-31 in which the primary effect of bilayer interaction is the down-regulation of the ψ_s of mitochondrial membranes. In support of this model, we found that SS peptides caused the partial neutralization of the strong negative surface charge density of mitochondrial membranes, but appeared to do so in a way that does not cause positive charge overcompensation (Figures 3B and 6C).

D. SS peptide mitochondrial distribution

In our binding model, it is important to consider how peptides may distribute within the membrane interface. SS peptide occupancy at or near the surface of anionic membranes is driven by the high ($Z = +3$) positive charge density that dictates peptide equilibrium distribution in the electrostatic field of the bilayer and promotes ionic interactions with negatively charged membrane-bound groups. Hence, SS peptides bind the membrane surface with micromolar affinity that is entropically and enthalpically favored (Figure 2); however, given that the binding is relatively weak and reversible depending on local ionic strength (Figure 3), a fraction of these small polybasic amphiphiles are likely to exist untethered to the membrane surface, but stably reside within the electric field of the double diffuse layer of the anionic mitochondrial membranes (Figure 8B). This distribution is likely to vary laterally along the MIM in different mitochondrial subcompartments that have different surface charge characteristics. Noting that ion distribution near a charged membrane surface is an exponential function of formal charge (Eq. S11), the polybasic nature of SS peptides is critical in allowing them to bind anionic bilayers in competition

with physiological concentrations of mono- and divalent cations (trivalent cations are rare in biological systems). Hence, in accordance with GCS theory (Figure 7A), the accumulation of SS peptides at the interface will have the effect of shifting the balance of other ionic species (e.g., solvated metal ions and amphitropic basic proteins) away form the membrane surface. Finally, given that CL may be enriched on the inner leaflet of the MIM (71), SS peptides may preferentially accumulate on the matrix-facing side of this membrane, provided that a pathway exists for them to traverse the MIM. Ongoing work in our group is aimed toward understanding the dynamic distribution of SS peptides among mitochondrial subcompartments.

Supporting Information - References

1. Devaux, P. F., and Seigneuret, M. (1985) Specificity of lipid-protein interactions as determined by spectroscopic techniques. *Biochim Biophys Acta* **822**, 63-125
2. Reshetnyak, Y. K., Andreev, O. A., Segala, M., Markin, V. S., and Engelman, D. M. (2008) Energetics of peptide (pHLIP) binding to and folding across a lipid bilayer membrane. *Proc Natl Acad Sci U S A* **105**, 15340-15345
3. Santos, N. C., Prieto, M., and Castanho, M. A. (2003) Quantifying molecular partition into model systems of biomembranes: an emphasis on optical spectroscopic methods. *Biochim Biophys Acta* **1612**, 123-135
4. Greenwood, A. I., Tristram-Nagle, S., and Nagle, J. F. (2006) Partial molecular volumes of lipids and cholesterol. *Chem Phys Lipids* **143**, 1-10
5. Pan, J., Cheng, X., Sharp, M., Ho, C. S., Khadka, N., and Katsaras, J. (2015) Structural and mechanical properties of cardiolipin lipid bilayers determined using neutron spin echo, small angle neutron and X-ray scattering, and molecular dynamics simulations. *Soft Matter* **11**, 130-138
6. Pan, J., Heberle, F. A., Tristram-Nagle, S., Szymanski, M., Koepfinger, M., Katsaras, J., and Kucerka, N. (2012) Molecular structures of fluid phase phosphatidylglycerol bilayers as determined by small angle neutron and X-ray scattering. *Biochim Biophys Acta* **1818**, 2135-2148
7. Sathappa, M., and Alder, N. N. (2016) The ionization properties of cardiolipin and its variants in model bilayers. *Biochim Biophys Acta* **1858**, 1362-1372
8. McLaughlin, S. (1977) Electrostatic potentials at membrane-solution interfaces. *Curr. Top. Membr. Transp.* **9**, 71-144
9. Oshima, H. (2010) *Biophysical Chemistry of Interfaces*, Wiley, Hoboken
10. McGibbon, R. T., Beauchamp, K. A., Harrigan, M. P., Klein, C., Swails, J. M., Hernandez, C. X., Schwantes, C. R., Wang, L. P., Lane, T. J., and Pande, V. S. (2015) MDTraj: A Modern Open Library for the Analysis of Molecular Dynamics Trajectories. *Biophys J* **109**, 1528-1532
11. Abraham, M. J., Murtola, T., Schulz, R., Pail, S., Smith, J. C., Hess, B., and Lindahl, E. (2015) GROMACS: High performance molecular simulations through multi-level parallelism from laptops to supercomputers. *SoftwareX* **1-2**, 19-25
12. Hess, B., Kutzner, C., van der Spoel, D., and Lindahl, E. (2008) GROMACS 4: Algorithms for Highly Efficient, Load-Balanced, and Scalable Molecular Simulation. *J Chem Theory Comput* **4**, 435-447
13. Eisenhaber, F., Linjnzaad, P., Argos, P., Sander, C., and Scharf, M. (1995) The double cubic lattice method: efficient approaches to numerical integration of surface area and volume and dot surface contouring of molecular assemblies. *The Journal of Computational Chemistry* **16**, 273-284

14. Humphrey, W., Dalke, A., and Schulten, K. (1996) VMD: visual molecular dynamics. *J Mol Graph* **14**, 33-38, 27-38
15. Hunter, J. D. (2007) Matplotlib: A 2D graphics environment. *Computing In Science and Engineering* **9**, 90-95
16. Lakowicz, J. R. (2006) *Principles of Fluorescene Spectroscopy, Third Edition*, Springer, Singapore
17. Antosiewicz, J. M., and Shugar, D. (2016) UV-Vis spectroscopy of tyrosine side-groups in studies of protein structure. Part 1: basic principles and properties of tyrosine chromophore. *Biophys Rev* **8**, 151-161
18. Antosiewicz, J. M., and Shugar, D. (2016) UV-Vis spectroscopy of tyrosine side-groups in studies of protein structure. Part 2: selected applications. *Biophys Rev* **8**, 163-177
19. Fornander, L. H., Feng, B., Beke-Somfai, T., and Norden, B. (2014) UV transition moments of tyrosine. *J Phys Chem B* **118**, 9247-9257
20. Aivazian, D., and Stern, L. J. (2000) Phosphorylation of T cell receptor zeta is regulated by a lipid dependent folding transition. *Nat Struct Biol* **7**, 1023-1026
21. Douliez, J., Michon, T., and Marion, D. (2000) Steady-state tyrosine fluorescence to study the lipid-binding properties of a wheat non-specific lipid-transfer protein (nsLTP1). *Biochim Biophys Acta* **1467**, 65-72
22. Poveda, J. A., Prieto, M., Encinar, J. A., Gonzalez-Ros, J. M., and Mateo, C. R. (2003) Intrinsic tyrosine fluorescence as a tool to study the interaction of the shaker B "ball" peptide with anionic membranes. *Biochemistry* **42**, 7124-7132
23. Permayakov, E. A. (2017) *Luminescent Spectroscopy of Proteins*, CRC Press
24. Seelig, J. (1997) Titration calorimetry of lipid-peptide interactions. *Biochim Biophys Acta* **1331**, 103-116
25. Birk, A. V., Chao, W. M., Bracken, C., Warren, J. D., and Szeto, H. H. (2014) Targeting mitochondrial cardiolipin and the cytochrome c/cardiolipin complex to promote electron transport and optimize mitochondrial ATP synthesis. *Br J Pharmacol* **171**, 2017-2028
26. Birk, A. V., Chao, W. M., Liu, S., Soong, Y., and Szeto, H. H. (2015) Disruption of cytochrome c heme coordination is responsible for mitochondrial injury during ischemia. *Biochim Biophys Acta* **1847**, 1075-1084
27. Birk, A. V., Liu, S., Soong, Y., Mills, W., Singh, P., Warren, J. D., Seshan, S. V., Pardee, J. D., and Szeto, H. H. (2013) The mitochondrial-targeted compound SS-31 re-energizes ischemic mitochondria by interacting with cardiolipin. *J Am Soc Nephrol* **24**, 1250-1261
28. Szeto, H. H. (2014) First-in-class cardiolipin-protective compound as a therapeutic agent to restore mitochondrial bioenergetics. *Br J Pharmacol* **171**, 2029-2050
29. Coutinho, A., and Prieto, M. (1993) Ribonuclease T1 and alcohol dehydrogenase fluorescence quenching by acrylamide: a laboratory experiment for undergraduate students. *J. Chem. Educ.* **70**, 425-428
30. Matsuzaki, K., Murase, O., Sugishita, K., Yoneyama, S., Akada, K., Ueha, M., Nakamura, A., and Kobayashi, S. (2000) Optical characterization of liposomes by right angle light scattering and turbidity measurement. *Biochim Biophys Acta* **1467**, 219-226
31. Wilschut, J., Nir, S., Scholma, J., and Hoekstra, D. (1985) Kinetics of Ca2+-induced fusion of cardiolipin-phosphatidylcholine vesicles: correlation between vesicle aggregation, bilayer destabilization, and fusion. *Biochemistry* **24**, 4630-4636
32. Ferre, R., Melo, M. N., Correia, A. D., Feliu, L., Bardaji, E., Planas, M., and Castanho, M. (2009) Synergistic effects of the membrane actions of cecropin-melittin antimicrobial hybrid peptide BP100. *Biophys J* **96**, 1815-1827
33. Troiano, J. M., McGeachy, A. C., Olenick, L. L., Fang, D., Liang, D., Hong, J., Kuech, T. R., Caudill, E. R., Pedersen, J. A., Cui, Q., and Geiger, F. M. (2017) Quantifying the Electrostatics of Polycation-Lipid Bilayer Interactions. *J Am Chem Soc* **139**, 5808-5816

34. Ben-Tal, N., Honig, B., Peitzsch, R. M., Denisov, G., and McLaughlin, S. (1996) Binding of small basic peptides to membranes containing acidic lipids: theoretical models and experimental results. *Biophys J* **71**, 561-575
35. Murray, D., Arbuzova, A., Hangyas-Mihalyne, G., Gambhir, A., Ben-Tal, N., Honig, B., and McLaughlin, S. (1999) Electrostatic properties of membranes containing acidic lipids and adsorbed basic peptides: theory and experiment. *Biophys J* **77**, 3176-3188
36. Tyler, A. I., Law, R. V., and Seddon, J. M. (2015) X-ray diffraction of lipid model membranes. *Methods Mol Biol* **1232**, 199-225
37. Heftberger, P., Kollmitzer, B., Heberle, F. A., Pan, J., Rappolt, M., Amenitsch, H., Kucerka, N., Katsaras, J., and Pabst, G. (2014) Global small-angle X-ray scattering data analysis for multilamellar vesicles: the evolution of the scattering density profile model. *J Appl Crystallogr* **47**, 173-180
38. Pabst, G., Rappolt, M., Amenitsch, H., and Laggner, P. (2000) Structural information from multilamellar liposomes at full hydration: full q-range fitting with high quality x-ray data. *Phys Rev E Stat Phys Plasmas Fluids Relat Interdiscip Topics* **62**, 4000-4009
39. Auger, M. (2000) Biological membrane structure by solid-state NMR. *Curr Issues Mol Biol* **2**, 119-124
40. Koynova, R., and Caffrey, M. (1998) Phases and phase transitions of the phosphatidylcholines. *Biochim Biophys Acta* **1376**, 91-145
41. Mandal, A., and van der Wel, P. C. A. (2016) MAS (1)H NMR Probes Freezing Point Depression of Water and Liquid-Gel Phase Transitions in Liposomes. *Biophys J* **111**, 1965-1973
42. Murzyn, K., Rog, T., and Pasenkiewicz-Gierula, M. (2005) Phosphatidylethanolamine-phosphatidylglycerol bilayer as a model of the inner bacterial membrane. *Biophys J* **88**, 1091-1103
43. Fenske, D. B., and Cullis, P. R. (1993) Acyl chain orientational order in large unilamellar vesicles: comparison with multilamellar liposomes: a 2H and 31P nuclear magnetic resonance study. *Biophys J* **64**, 1482-1491
44. Kaiser, R. D., and London, E. (1998) Location of diphenylhexatriene (DPH) and its derivatives within membranes: comparison of different fluorescence quenching analyses of membrane depth. *Biochemistry* **37**, 8180-8190
45. Jurkiewicz, P., Cwiklik, L., Jungwirth, P., and Hof, M. (2012) Lipid hydration and mobility: an interplay between fluorescence solvent relaxation experiments and molecular dynamics simulations. *Biochimie* **94**, 26-32
46. Parasassi, T., Krasnowska, E. K., Bagatolli, L., and Gratton, E. (1998) Laurdan and prodan as polarity-sensitive fluorescent membrane probes. *J Fluorescence* **8**, 365-373
47. Amaro, M., Reina, F., Hof, M., Eggeling, C., and Sezgin, E. (2017) Laurdan and Di-4-ANEPPDHQ probe different properties of the membrane. *J Phys D Appl Phys* **50**, 134004
48. Parasassi, T., De Stasio, G., Ravagnan, G., Rusch, R. M., and Gratton, E. (1991) Quantitation of lipid phases in phospholipid vesicles by the generalized polarization of Laurdan fluorescence. *Biophys J* **60**, 179-189
49. Boyd, K. J., Alder, N. N., and May, E. R. (2017) Buckling Under Pressure: Curvature-Based Lipid Segregation and Stability Modulation in Cardiolipin-Containing Bilayers. *Langmuir* **33**, 6937-6946
50. Boyd, K. J., Alder, N. N., and May, E. R. (2018) Molecular Dynamics Analysis of Cardiolipin and Monolysocardiolipin on Bilayer Properties. *Biophys J* **114**, 2116-2127
51. Wilson, B. A., Ramanathan, A., and Lopez, C. F. (2019) Cardiolipin-Dependent Properties of Model Mitochondrial Membranes from Molecular Simulations. *Biophys J* **117**, 429-444
52. Su, Y., Mani, R., Doherty, T., Waring, A. J., and Hong, M. (2008) Reversible sheet-turn conformational change of a cell-penetrating peptide in lipid bilayers studied by solid-state NMR. *J Mol Biol* **381**, 1133-1144

53. Lewis, R. N., and McElhaney, R. N. (2009) The physicochemical properties of cardiolipin bilayers and cardiolipin-containing lipid membranes. *Biochim Biophys Acta* **1788**, 2069-2079
54. General, I. J., and Asciutto, E. K. (2017) Structure and dynamics of Penetratin's association and translocation to a lipid bilayer. *AIP Advances* **7**, 035008
55. Tang, M., Waring, A. J., and Hong, M. (2007) Phosphate-mediated arginine insertion into lipid membranes and pore formation by a cationic membrane peptide from solid-state NMR. *J Am Chem Soc* **129**, 11438-11446
56. Cai, X., Yang, Q., Lin, J., Fu, N., and Wang, S. (2017) A Specific Peptide with Calcium-Binding Capacity from Defatted Schizochytrium sp. Protein Hydrolysates and the Molecular Properties. *Molecules* **22**
57. Carraro, M., Checchetto, V., Sartori, G., Kucharczyk, R., di Rago, J. P., Minervini, G., Franchin, C., Arrigoni, G., Giorgio, V., Petronilli, V., Tosatto, S. C. E., Lippe, G., Szabo, I., and Bernardi, P. (2018) High-Conductance Channel Formation in Yeast Mitochondria is Mediated by F-ATP Synthase e and g Subunits. *Cell Physiol Biochem* **50**, 1840-1855
58. Besarab, A., DeGuzman, A., and Swanson, J. W. (1981) Effect of albumin and free calcium concentrations on calcium binding in vitro. *J Clin Pathol* **34**, 1361-1367
59. Cullis, P. R., de Kruijff, B., Hope, M. J., Nayar, R., Rietveld, A., and Verkleij, A. J. (1980) Structural properties of phospholipids in the rat liver inner mitochondrial membrane. *Biochim Biophys Acta* **600**, 625-635
60. Gasanov, S. E., Shrivastava, I. H., Israilov, F. S., Kim, A. A., Rylova, K. A., Zhang, B., and Dagda, R. K. (2015) Naja naja oxiana Cobra Venom Cytotoxins CTI and CTII Disrupt Mitochondrial Membrane Integrity: Implications for Basic Three-Fingered Cytotoxins. *PLoS One* **10**, e0129248
61. Overall, S. A., Zhu, S., Hanssen, E., Separovic, F., and Sani, M. A. (2019) In Situ Monitoring of Bacteria under Antimicrobial Stress Using (31)P Solid-State NMR. *Int J Mol Sci* **20**
62. Sani, M. A., Keech, O., Gardestrom, P., Dufourc, E. J., and Grobner, G. (2009) Magic-angle phosphorus NMR of functional mitochondria: in situ monitoring of lipid response under apoptotic-like stress. *FASEB J* **23**, 2872-2878
63. Zhao, K., Zhao, G. M., Wu, D., Soong, Y., Birk, A. V., Schiller, P. W., and Szeto, H. H. (2004) Cell-permeable peptide antioxidants targeted to inner mitochondrial membrane inhibit mitochondrial swelling, oxidative cell death, and reperfusion injury. *J Biol Chem* **279**, 34682-34690
64. Ikon, N., and Ryan, R. O. (2017) Barth Syndrome: Connecting Cardiolipin to Cardiomyopathy. *Lipids* **52**, 99-108
65. Kim, J., Mosior, M., Chung, L. A., Wu, H., and McLaughlin, S. (1991) Binding of peptides with basic residues to membranes containing acidic phospholipids. *Biophys J* **60**, 135-148
66. Shewan, A., Eastburn, D. J., and Mostov, K. (2011) Phosphoinositides in cell architecture. *Cold Spring Harb Perspect Biol* **3**, a004796
67. Bigay, J., and Antonny, B. (2012) Curvature, lipid packing, and electrostatics of membrane organelles: defining cellular territories in determining specificity. *Dev Cell* **23**, 886-895
68. Jakovcic, S., Swift, H. H., Gross, N. J., and Rabinowitz, M. (1978) Biochemical and stereological analysis of rat liver mitochondria in different thyroid states. *J Cell Biol* **77**, 887-901
69. Zhao, K., Luo, G., Zhao, G. M., Schiller, P. W., and Szeto, H. H. (2003) Transcellular transport of a highly polar 3+ net charge opioid tetrapeptide. *J Pharmacol Exp Ther* **304**, 425-432
70. Kumar, P., Kizhakkedathu, J. N., and Straus, S. K. (2018) Antimicrobial Peptides: Diversity, Mechanism of Action and Strategies to Improve the Activity and Biocompatibility In Vivo. *Biomolecules* **8**
71. Krebs, J. J., Hauser, H., and Carafoli, E. (1979) Asymmetric distribution of phospholipids in the inner membrane of beef heart mitochondria. *J Biol Chem* **254**, 5308-5316

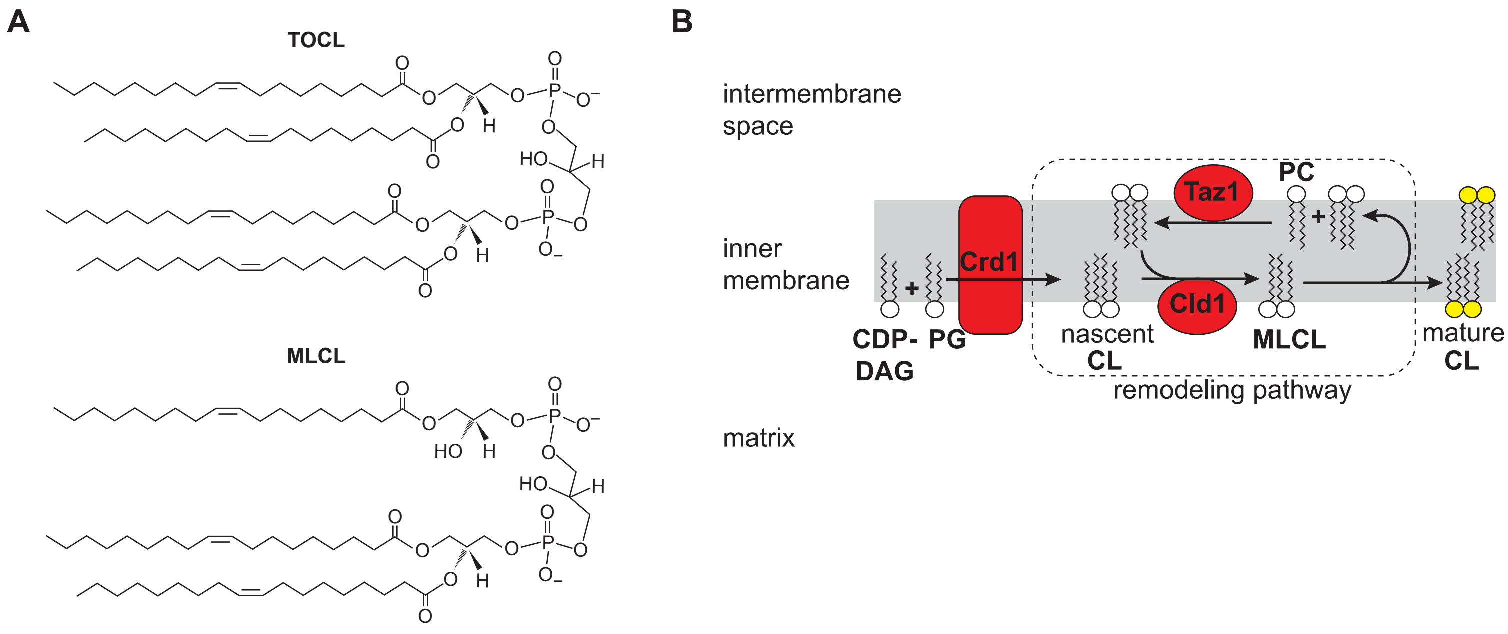

Figure S1. Cardiolipin structure and biogenesis. *A*, Chemical structures of tetraoleoyl cardiolipin (TOCL) and monolysocardiolipin (MLCL). *B*, Cardiolipin biosynthesis and remodeling in yeast. The committed step of CL synthesis is the condensation of phosphatidylglycerol (PG) and CDP-diacylglycerol (CDP-DAG) by cardiolipin synthase (Crd1) to produce nascent CL. The remodeling cycle that produces mature CL is initiated by the lipase Cld1, which removes an acyl chain to generate MLCL. The acyl-CoA independent transacylase tafazzin (Taz1) then mediates the transfer of an acyl chain from a donor phospholipid (e.g., PC) to regenerate tetra-acyl CL. This remodeling pathway yields mature CL species that are enriched in unsaturated fatty acids.

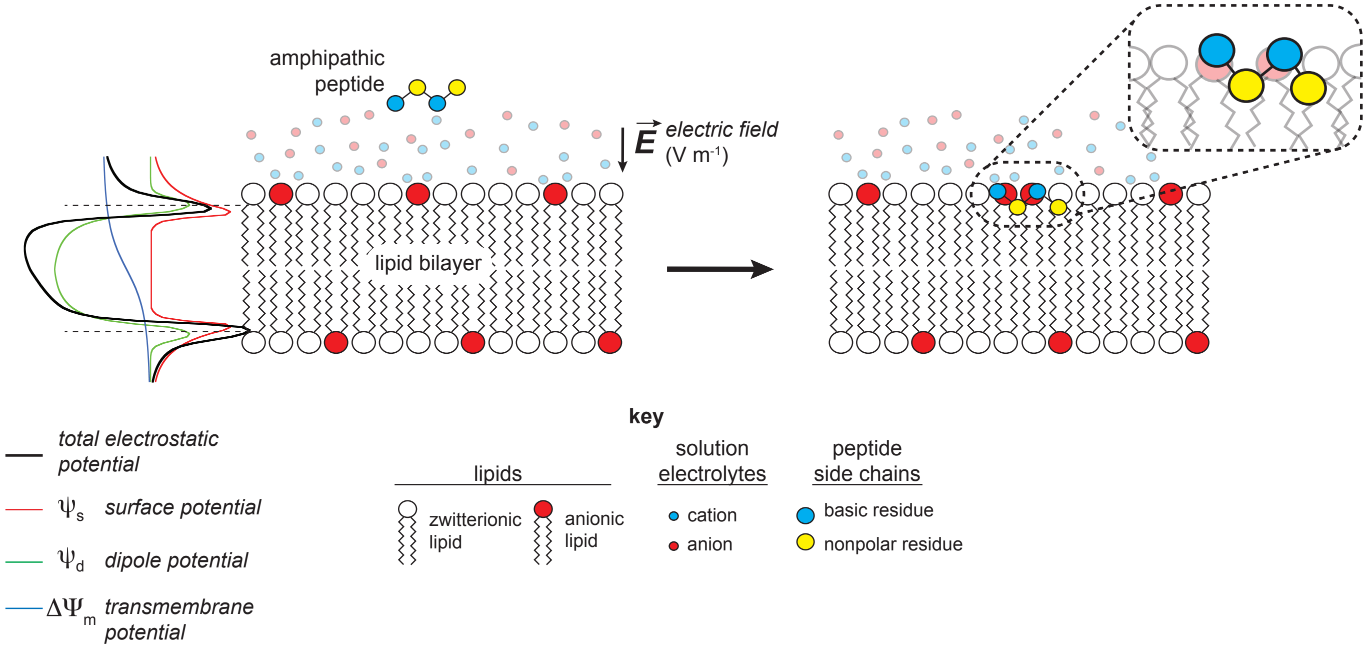

Figure S2. The binding of amphipathic peptides to anionic membranes. The electrostatic potential of biomembranes consists of three superimposed potentials: the surface potential (ψ_s, arising from the net charges in the membrane interface); the dipole potential (ψ_d, from intramolecular dipole moments of interfacial chemical groups and water molecules), and the transmembrane potential ($\Delta\Psi_m$, from charge imbalance of aqueous solutions on either side of the membrane).

Peptides containing both basic and nonpolar side chains bind to lipid bilayers with a net negative charge *via* electrostatic and hydrophobic interactions. A lipid bilayer can be characterized by the surface charge density (σ, C m^{-2}), based on the number of formal charges imparted by ionized headgroups per membrane surface area. σ is related to surface potential (ψ_s, mV) by Gouy Chapman-Stern formalism. The surface charge of anionic bilayers creates a strong electric field (***E***) that attracts positively charged ions and molecules (governed by Boltzmann statistics), creating a diffuse double layer with an enrichment of cations near the bilayer surface. Cations can electrostatically shield the negative charge density of the bilayer and may undergo complexation with lipid headgroups with an appreciable association constant (K^{ion}). An amphipathic peptide such as SS-31 is attracted to the bilayer surface by long-range Coulombic forces originating from the lipid surface charges. This has the effect of accumulating such basic peptides at the bilayer surface at concentrations significantly larger than the bulk peptide concentration. At the bilayer, the peptide basic side chains can form electrostatic interactions with anionic lipid headgroups and the nonpolar side chains can intercalate into the acyl chain region, stabilized by the hydrophobic effect.

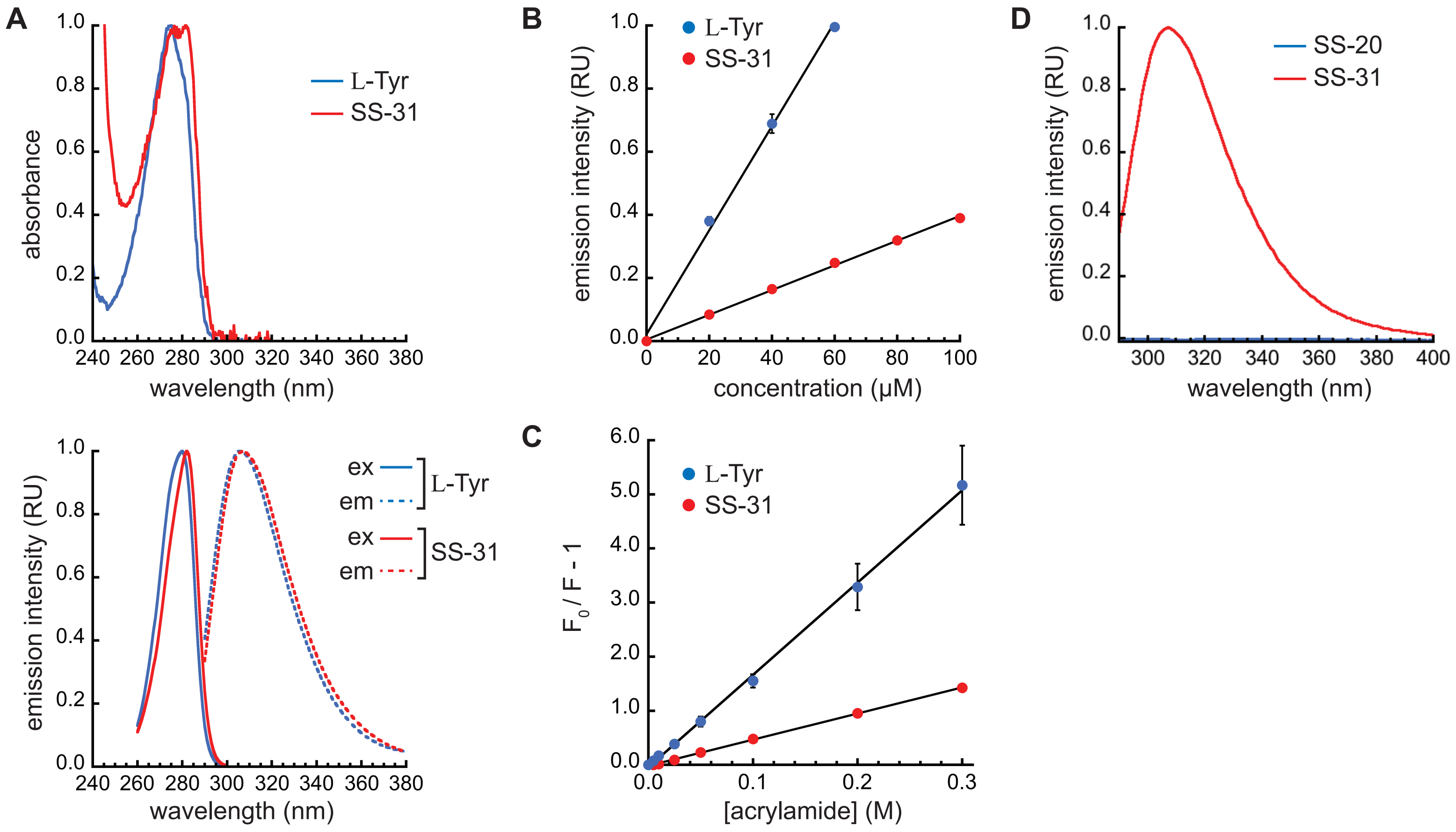

Figure S3. Fluorescence characterization of SS-31 in aqueous buffer. *A*, Normalized absorption spectra (upper panel) and excitation/emission spectra (lower panel) of 20 µM L-Tyr (blue) and 20 µM SS-31 (red). All traces are signal averages of n=3 independent samples. *B*, Emission intensity of increasing concentrations of L-Tyr (blue) and SS-31 (red) (n=3 ± SD). Lines represent linear fits to the data (R>0.99 for both L-Tyr and SS-31 regressions). *C*, Stern-Volmer plots of fluorescence quenching of L-Tyr (blue) and SS-31 (red) by acrylamide (n=3 ± SD). Lines represent fits to the Stern-Volmer equation (R>0.99 for both L-Tyr and SS-31 regressions). *D*, Normalized emission spectra of 20 µM SS-31 (red) and 20 µM SS-20 (blue).

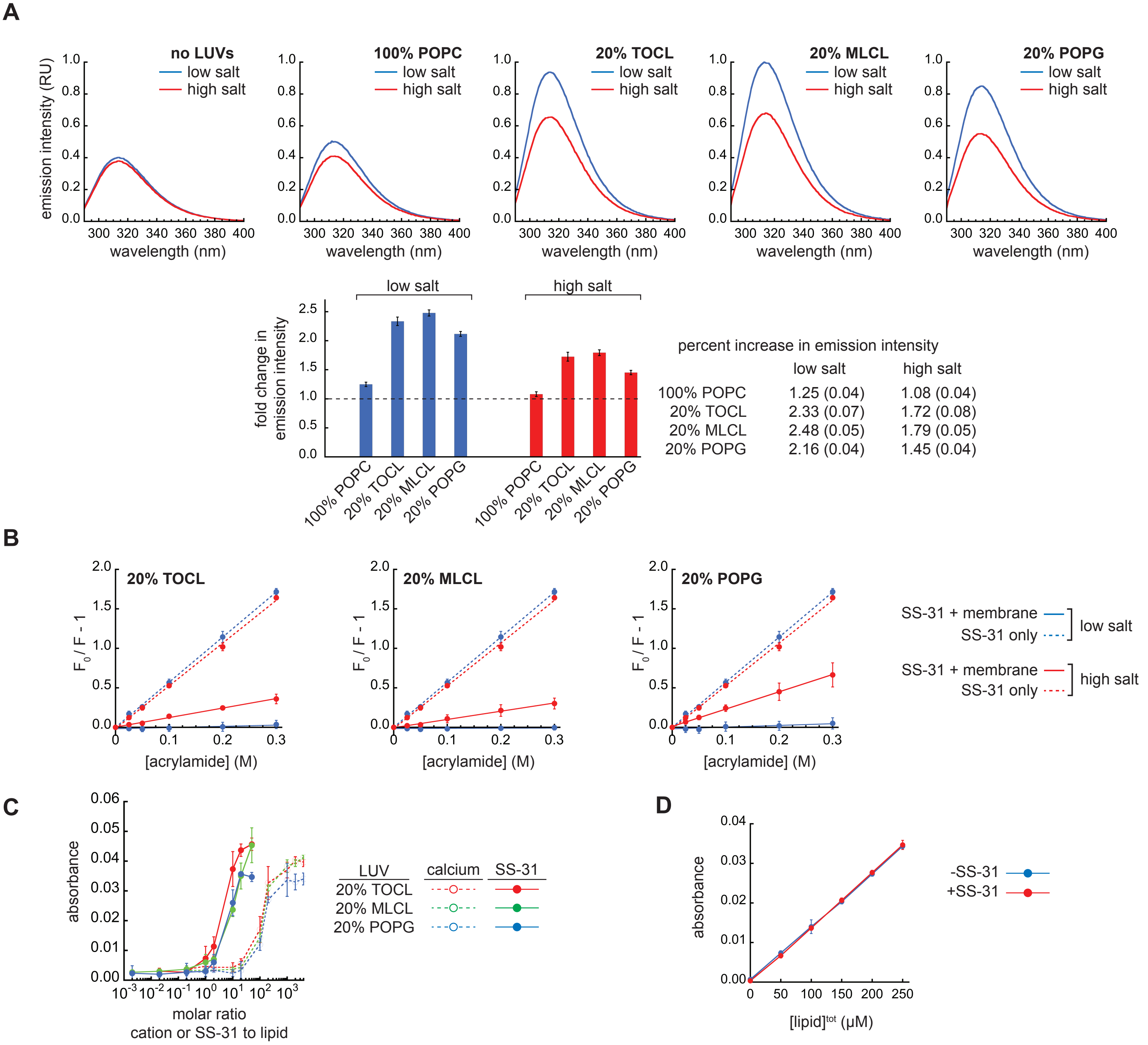

Figure S4. Fluorescence characterization of SS-31 membrane interactions. *A*, Above, normalized emission scans of 20 μM SS-31 (λ_{ex} = 281 nm, λ_{em} = 290 to 400 nm) in solution (no LUVs) and in the presence of LUVs composed of 100% POPC, or containing 20 mol% TOCL, MLCL, or POPG in a background of POPC as indicated. Measurements were made under low salt (blue) or high salt (red) conditions. Traces are signal averages of n=6 independent samples. Below, fold increase in SS-31 emission for lipid-containing samples relative to the respective (low or high salt) emission in the absence of LUVs. Values shown are means (n=6 ± SD) determined from the emission scans above. *B*, Stern-Volmer plots of acrylamide fluorescence quenching of 20 μM SS-31 in solution (dashed lines) or in the presence of LUVs containing 20 mol% TOCL, MLCL, or POPG as indicated (solid lines) under low salt (blue) or high salt (red) conditions (n=3 ± SD). Lines represent fits to the Stern-Volmer equation (R>0.99 for all regressions). Note that data for SS-31 in solution (dashed line fits) are from a single set of experiments but are shown in all panels for comparison. *C* and *D*, Absorbance-based analysis of liposome aggregation. LUVs were incubated with the indicated salts or peptide followed by measurements of absorbance at 440 nm. C) LUVs ($[lipid]^{tot}$ = 25 μM) with the indicated lipid composition were preincubated with $CaCl_2$ ("calcium") or peptide ("SS-31") at different molar ratios followed by turbidity measurements (n=3 ± SD). D) LUVs ($[lipid]^{tot}$ = 0 to 250 μM) were preincubated with or without SS-31 at a 1:10 molar ratio (peptide : $[lipid]^{tot}$) followed by turbidity measurements (n=3 ± SD).

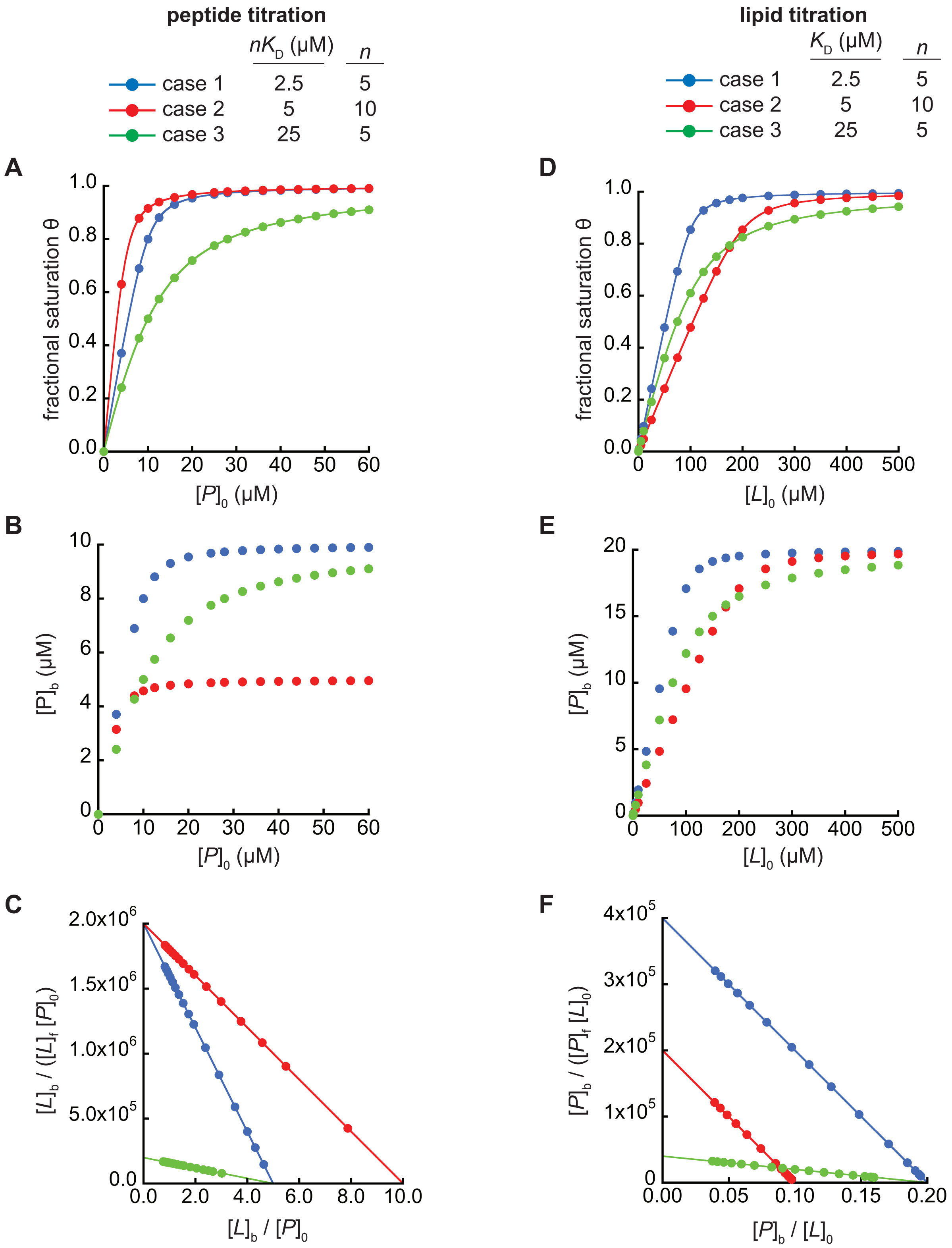

Figure S5. Quantitative analysis of peptide membrane binding. Simulated peptide-bilayer binding data are shown for three scenarios: case 1 (blue: high affinity, low *n*), case 2 (red: mid affinity, high *n*), and case 3 (green: low affinity, low *n*). *A-C*, titration of a fixed concentration of lipid ($[L]_0$=50 μM) with peptide ($[P]_0$, up to 60 μM); *D-F*, titration of a fixed concentration of peptide ($[P]_0$=20 μM) with lipid ($[L]_0$, up to 500 μM). *A,D*: Binding plots showing fractional saturation derived from Eq. S2 (panel A) or Eq. S3 (panel E). *B,E*: Plots of bound peptide as a function of titrant *P* (panel B) or *L* (panel E). *C,F*: Scatchard analyses derived from Eq. S6 (panel C) or Eq. S8 (panel F).

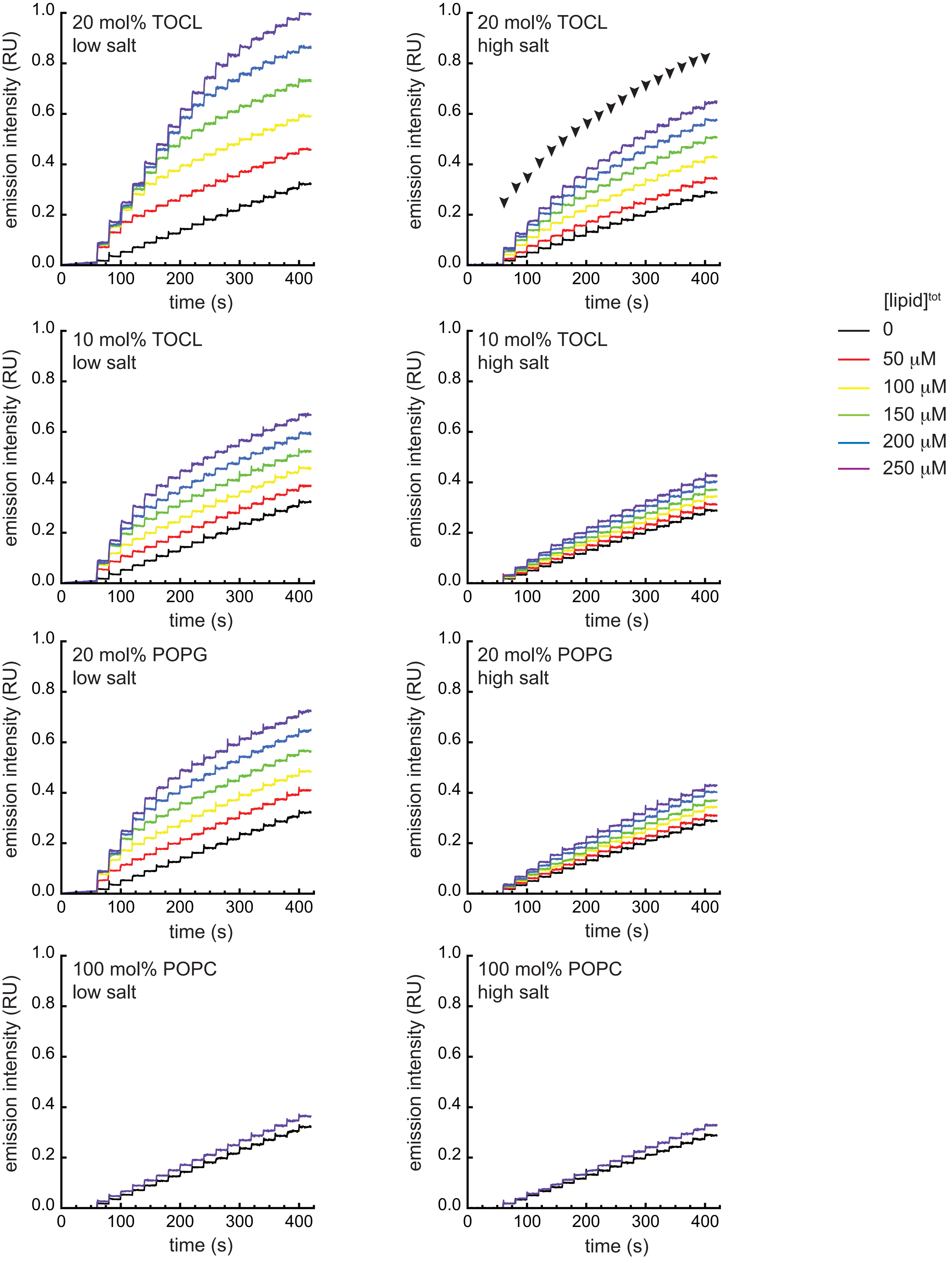

Figure S6. Kinetic measurements of SS-31 addition to LUVs. Titration of different concentrations of LUVs ($[\text{lipid}]^{\text{tot}}$ = 0, 50, 100, 150, 200, and 250 μM as indicated) with SS-31 at 2 μM increments. Peptide injections commenced at t=60 s and continued at 20 s intervals for a total of 18 injections ($[\text{SS-31}]^{\text{final}}$ = 36 μM). Injection points are displayed as arrowheads for the 20 mol% TOCL high salt sample. Note that the low- and high-salt time courses for peptide addition in the absence of membrane ($[\text{lipid}]^{\text{tot}}$ = 0, black traces) are identical, but are shown in each panel for comparison.

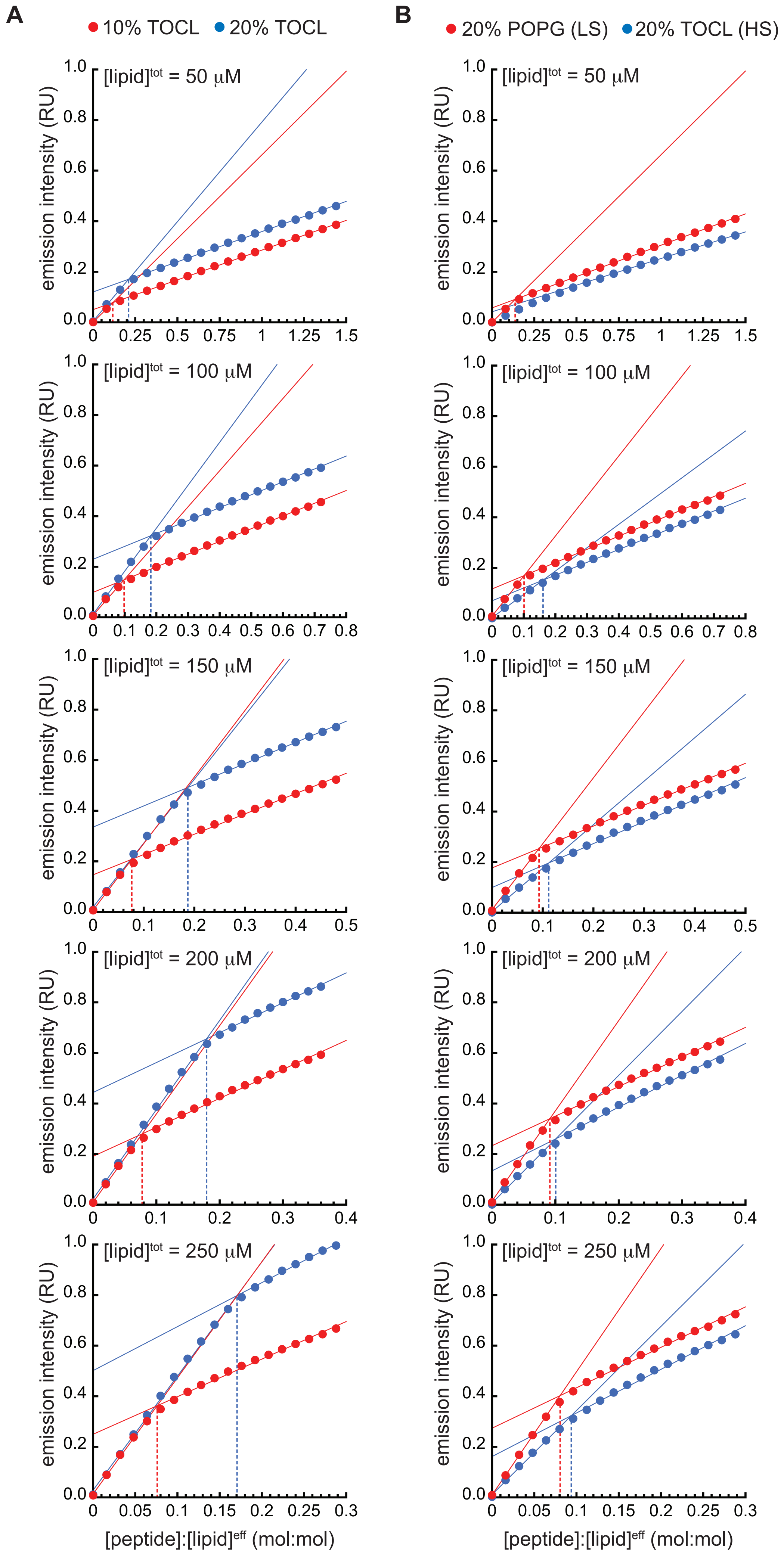

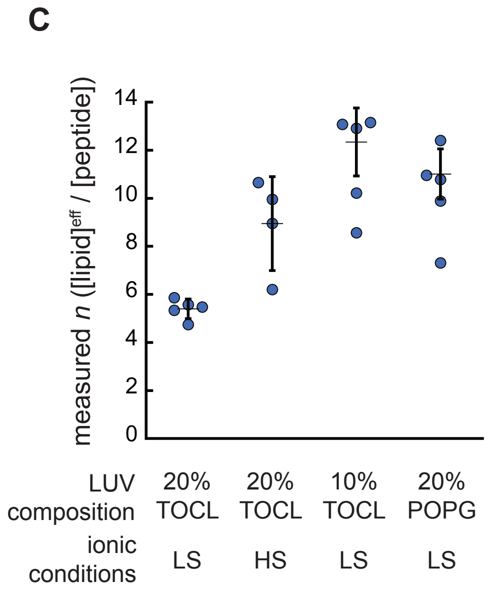

Figure S7. Analysis of time course binding saturation measurements. SS-31 fluorescence as a function of [peptide]:[lipid]^{eff} (mol:mol) from peptide titrations into solutions of LUVs at the total lipid concentration indicated. Calculated inflection points for each titration delineate pre- and post-saturation stages of the titration, with each stage fit to a linear function shown by lines with cognate colors. Dashed drop lines to the abscissa mark the [peptide]:[lipid]^{eff} at the inflection point for each titration. *A*, SS-31 titrations of LUVs composed of 10% TOCL (*red*) or 20% TOCL (*blue*) under low salt conditions. *B*, SS-31 titrations of LUVs composed of 20% POPG under low salt conditions (*red*) or 20% TOCL under high salt conditions (*blue*). *C*, Measured values of [lipid]^{eff}:[peptide] (=n) for each LUV type and salt condition shown in panels A and B. Individual points are shown with means ± SD.

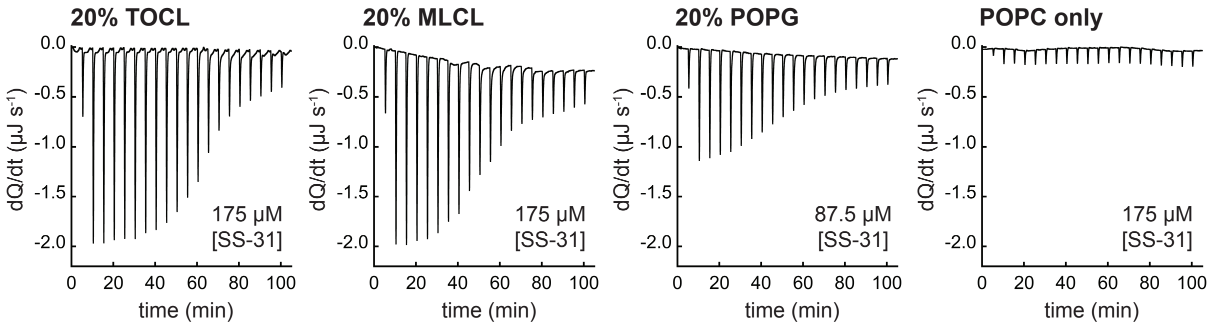

Figure S8. Microcalorimetry analysis of SS-31 membrane binding. Representative ITC raw data (heat flow time courses) obtained by titration of SS-31 at the indicated concentrations with LUVs of different lipid composition. Downward peaks represent exothermic events from injections of LUVs (10 nmol effective lipid each).

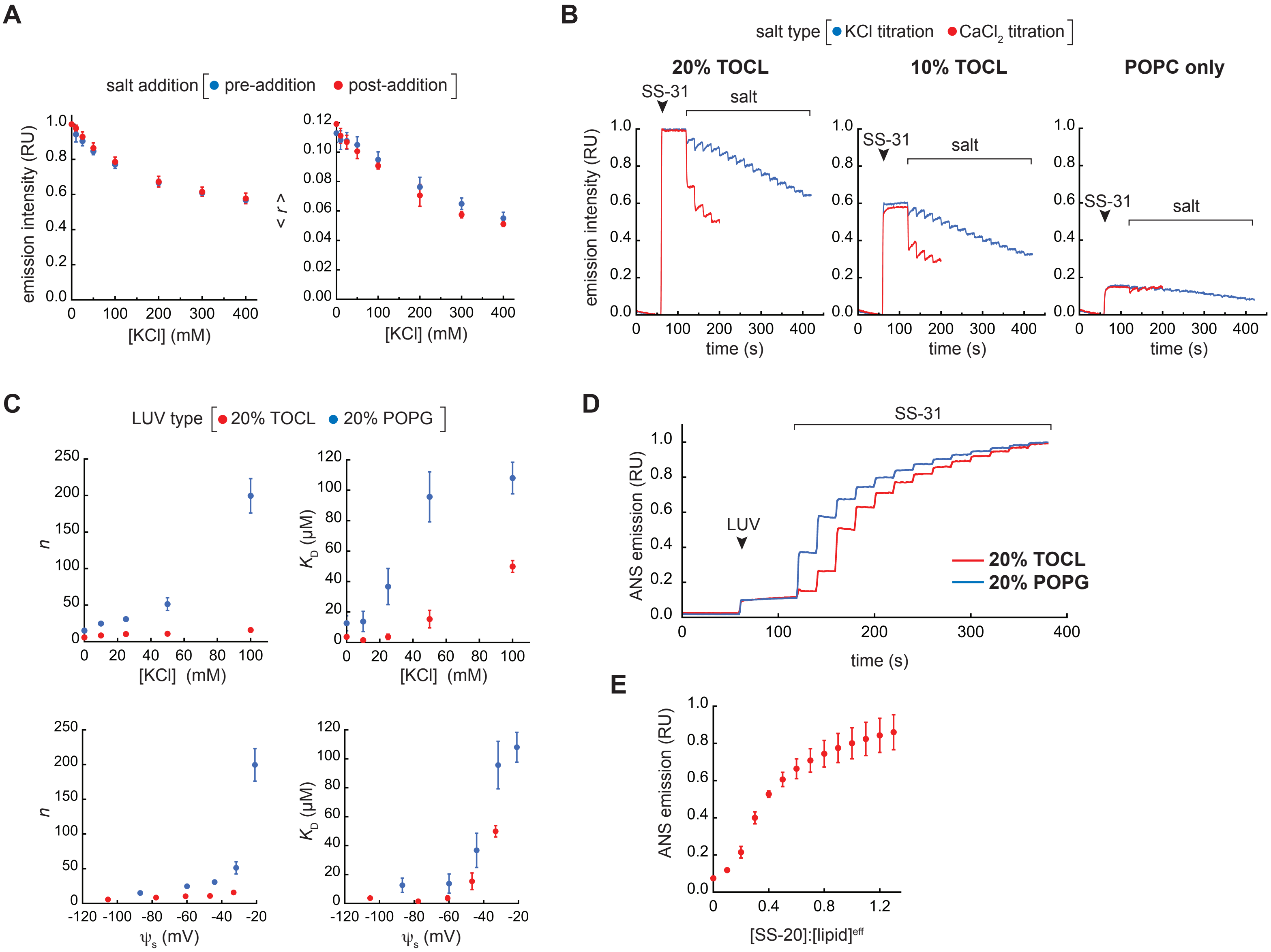

Figure S9. Relationship between SS peptide binding, ionic strength, and surface potential. *A*, Effect of pre- and post-salt addition. Different concentrations of KCl were added to LUVs (20 mol% TOCL) before binding of SS-31 (pre-addition, *blue*) or after the binding of SS-31 (post-addition, *red*) followed by measurements of emission intensity (*left*) or steady state anisotropy (*right*) of SS-31. *B*, Time courses of ionic strength titration. Representative time courses of endogenous SS-31 emission in the presence of LUVs with progressive addition of salts. At t=60 s (*black arrowheads*), SS-31 was added to suspensions of LUVs (20 mol% TOCL, 10 mol% TOCL, or POPC only). Titration of salts (starting at t=120 s) proceeded by addition of KCl (15 μmol increments, *blue traces*) or $CaCl_2$ (0.5 μmol increments, *red traces*) at 20 s intervals. Ca^{2+} titrations were truncated prior to the point of calcium-induced lipid aggregation (determined from Figure S4C). *C*, Salt-dependent equilibrium binding parameters. Binding isotherms shown in Figure 3A were used to calculate *n* and K_D at different ionic strengths, shown as a function of [KCl] (*upper panels*) and as a function of ψ_s, calculated from GCS formalism, Eq. S10-S12 (*lower panels*). *D*, Time courses of ANS emission with SS-31 titration. Representative time courses of ANS emission with SS-31 addition to suspensions of LUVs. At t=60 s (*black arrowhead*), LUVs composed of 20 mol% TOCL (*red trace*) or 20 mol% POPG (*blue trace*) were added to measurement buffer ([ANS] = 1 μM) at a concentration of $[lipid]^{eff}$ = 50 μM. Titration of SS-31 (starting at t=120 s) proceeded by peptide addition (10 nmol increments) at 20 s intervals. *E*, Effect of SS-20 on surface potential. Measurements of ANS fluorescence are shown as a function of $[SS\text{-}20]:[lipid]^{eff}$ for membranes containing 20% TOCL. Values shown are means (n=4 ± SD).

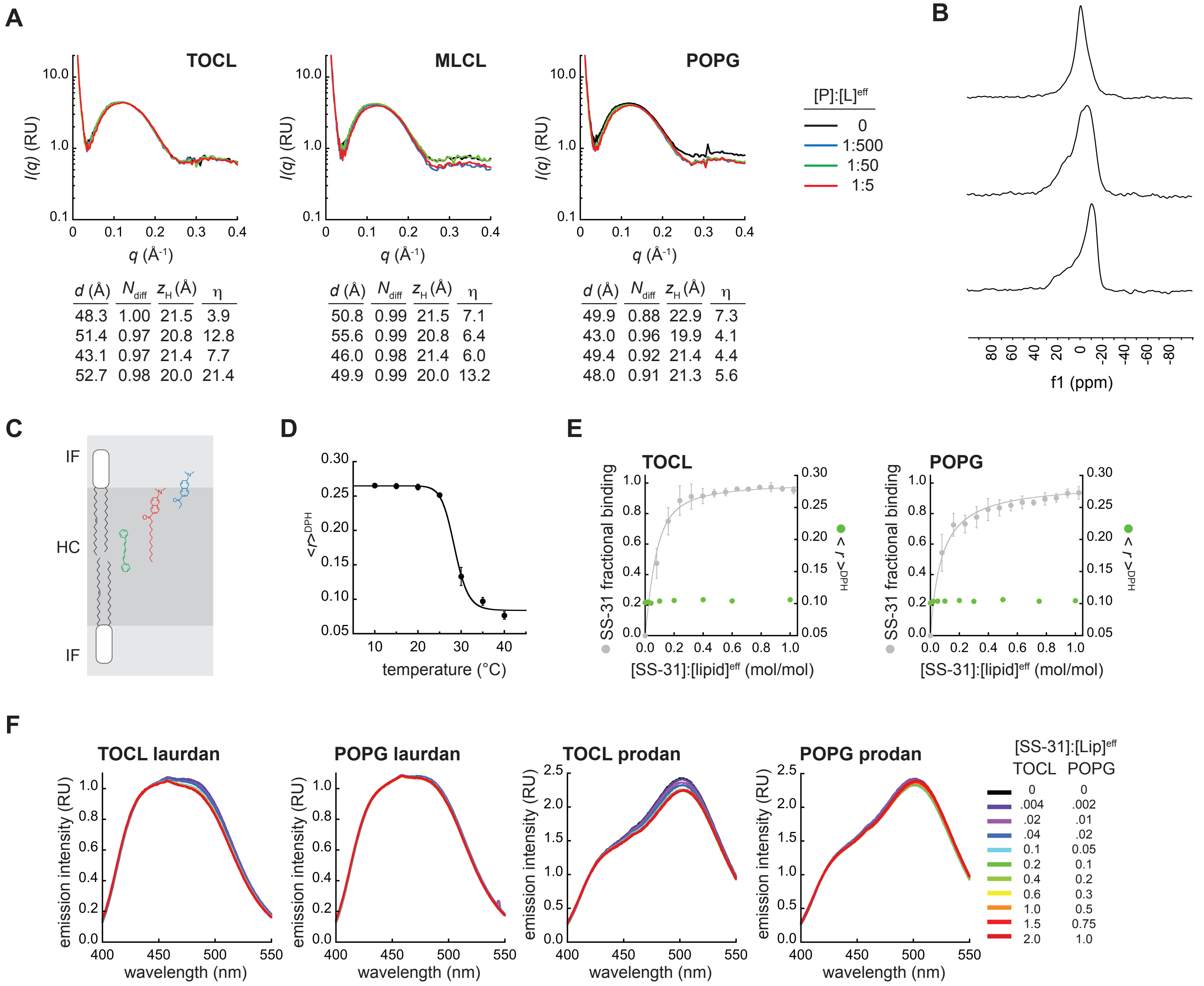

TOCL

d (Å)	N_{diff}	z_H (Å)	η
48.3	1.00	21.5	3.9
51.4	0.97	20.8	12.8
43.1	0.97	21.4	7.7
52.7	0.98	20.0	21.4

MLCL

d (Å)	N_{diff}	z_H (Å)	η
50.8	0.99	21.5	7.1
55.6	0.99	20.8	6.4
46.0	0.98	21.4	6.0
49.9	0.99	20.0	13.2

POPG

d (Å)	N_{diff}	z_H (Å)	η
49.9	0.88	22.9	7.3
43.0	0.96	19.9	4.1
49.4	0.92	21.4	4.4
48.0	0.91	21.3	5.6

Figure S10. Characterization of the effects of SS-31 on model membranes. *A*, Synchrotron SAXS. Background-corrected scattering density profiles are shown for LUVs containing anionic lipid (20% TOCL, MLCL, or POPG) or POPC only with the SS-31:lipid molar ratios indicated. Values reported in the table below are taken from fits of the data to Eq. S13 using the Global Analysis Program (GAP). *B*, ^{31}P ssNMR. ^{31}P NMR spectra of lipids (80 mol% POPC, 20 mol% TOCL) prepared as: (i) MLVs (*lower trace*), (ii) MLVs with freeze/thaw cycles (*middle trace*), or (iii) 100 nm LUVs made by extrusion (*upper trace*). *C*, Membrane fluorescent probes. Approximate positions of fluorescent reporters DPH (*green*), laurdan (*red*) and prodan (*blue*) relative to the interfacial region (IF) and hydrocarbon core (HC) of the lipid bilayer. *D-E*, Anisotropy measurements of DPH-containing membranes. *D*, LUVs containing 80 mol% DMPC and 20 mol% TMCL with DPH were incubated in temperatures at 5°C increments and measured for steady-state anisotropy of DPH ($<r>^{DPH}$). Values shown are means (n=3 ± SD). Fit to a sigmoidal function (*black line*) yields a maximal $<r>^{DPH}$ of 0.265 (corresponding to the Lβ phase), a minimal $<r>^{DPH}$ of 0.084 (corresponding to the Lα phase), and an inflection point at 28.5°C. *E*, LUVs containing 80 mol% POPC and 20 mol% TOCL or POPG as indicated were incubated with increasing [SS-31] and measured for $<r>^{DPH}$. Values shown are means (n=3 ± SD). Data and fits in gray show SS-31 fractional saturation (calculated from Fig. 2A, $[lipid]^{eff}$ = 25 μM). *F*, Emission scans of laurdan- and prodan-containing membranes. LUVs composed of 20% TOCL or 20% POPG, containing laurdan or prodan as indicated, were incubated in the presence of varying $[SS\text{-}31]:[lipid]^{eff}$ as shown. Scans are signal averages (n=3) of background-subtracted spectra, normalized relative to λ_{em}=440 nm (laurdan) or λ_{em} = 420 nm (prodan) to facilitate visualization.

A

20 mol% TOCL	w/ SS31	No SS31
TOCL P1	1.92	1.97
	1.89 - 1.94	1.97 - 1.98
TOCL P3	1.92	1.96
	1.89 - 1.94	1.95 - 1.97
POPC P	2.00	2.00
	1.99 - 2.01	1.99 - 2.01
**20 mol% MLCL**	**w/ SS31**	**No SS31**
MLCL P1	1.90	1.93
	1.88 - 1.91	1.92 - 1.94
MLCL P3	1.97	2.02
	1. 95 - 1.99	2.01 - 2.03
POPC P	1.94	1.93
	1.93 - 1.95	1.93 - 1.94
**20 mol% POPG**	**w/ SS31**	**No SS31**
POPG P	1.88	1.92
	1.86 - 1.89	1.92 - 1.93
POPC P	1.95	1.95
	1.94 - 1.95	1.95 - 1.96

B

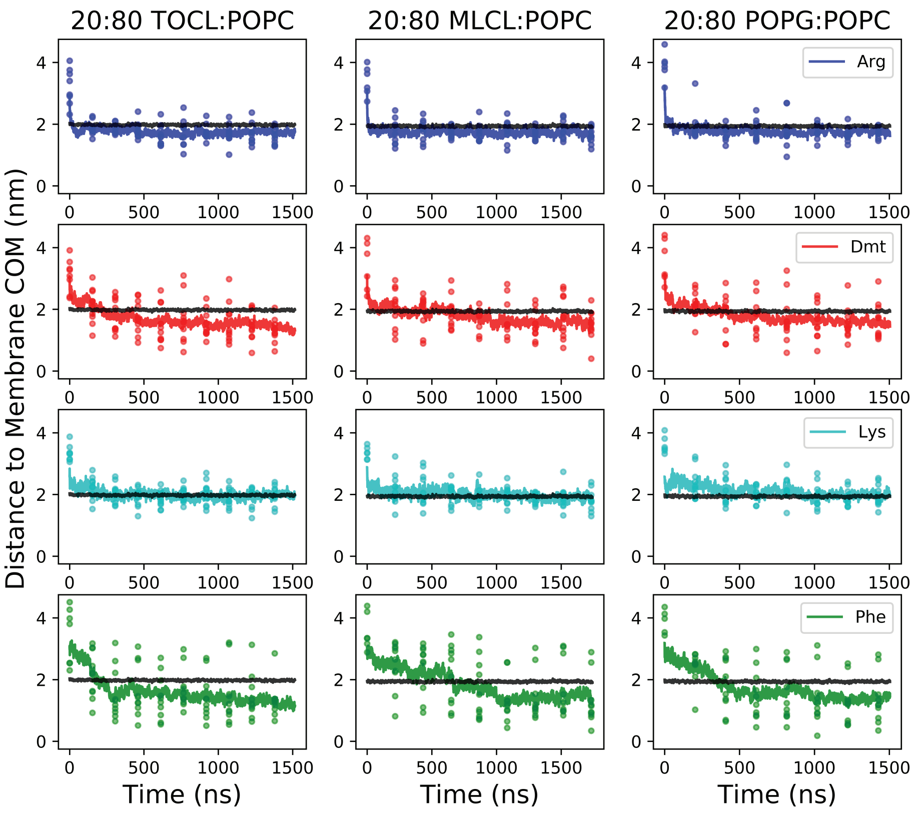

C

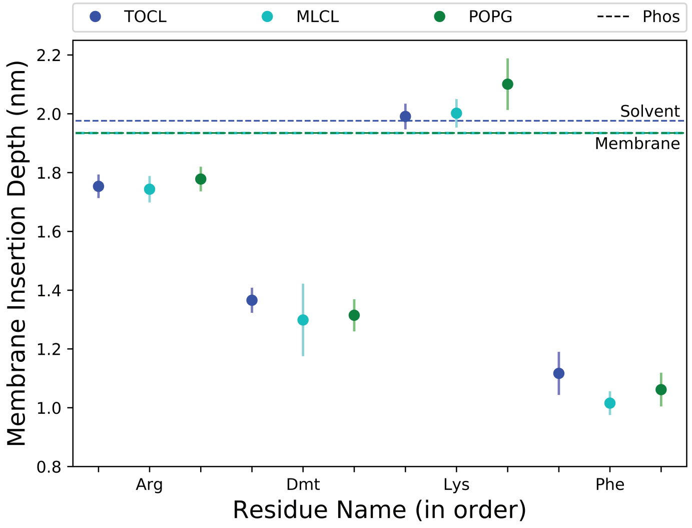

SS-31 Landmark Atoms	20:80 TOCL:POPC		20:80 MLCL:POPC		20:80 POPG:POPC	
	Z^{pos} (nm)	95% CI	Z^{pos} (nm)	95% CI	Z^{pos} (nm)	95% CI
**Arg, C_ζ**	1.75	1.71 - 1.79	1.74	1.70 - 1.79	1.78	1.74 - 1.82
**Dmt, O_η**	1.37	1.32 - 1.41	1.30	1.18 - 1.42	1.31	1.26 - 1.37
**Lys, N_ζ**	1.99	1.95 - 2.03	2.00	1.95 - 2.05	2.10	2.01 - 2.19
**Phe, C_ζ**	1.12	1.04 - 1.19	1.02	0.98 - 1.06	1.06	1.00 - 1.12

Figure S11. MD simulations: lipid phosphate positions and SS-31 side chain insertion depths. *A*, Lipid-specific phosphate z-positions normalized to the bilayer center of mass (COM). Values shown for each lipid and phosphate type are means in nm (*upper box*) ± 95% CI (*lower box*). *B*, Time course profiles are shown for individual SS-31 side chain positions relative to bilayers composed of the indicated lipids. Positions along the z-axis were determined as the distance between each side chain and the calculated COM of the membrane. The black line shows the average position of all phosphates in the upper leaflet. Colored dots show the z coordinates (z^{pos}) of individual side chains, and colored lines represent the average z coordinates. *C*, Comparison of membrane insertion depths from MD simulations for the "peptide burial state". *Left,* average membrane insertion depths (z^{pos}, n=10 ± 95% CI) for landmark atoms of each side chain of SS-31 in bilayers of different lipid compositions indicated in comparison with average z^{pos} levels of headgroup phosphates. The dashed horizontal lines represent the COM of the upper leaflet phosphates; the 20% MLCL and 20% POPG systems are overlapping and are represented by the green line, and the 20% TOCL system is represented by the blue line. *Right*, table showing values of z^{pos} of SS-31 side chains. Each residue assumed a distinct membrane insertion depth with no significant difference observed among the different lipid systems.

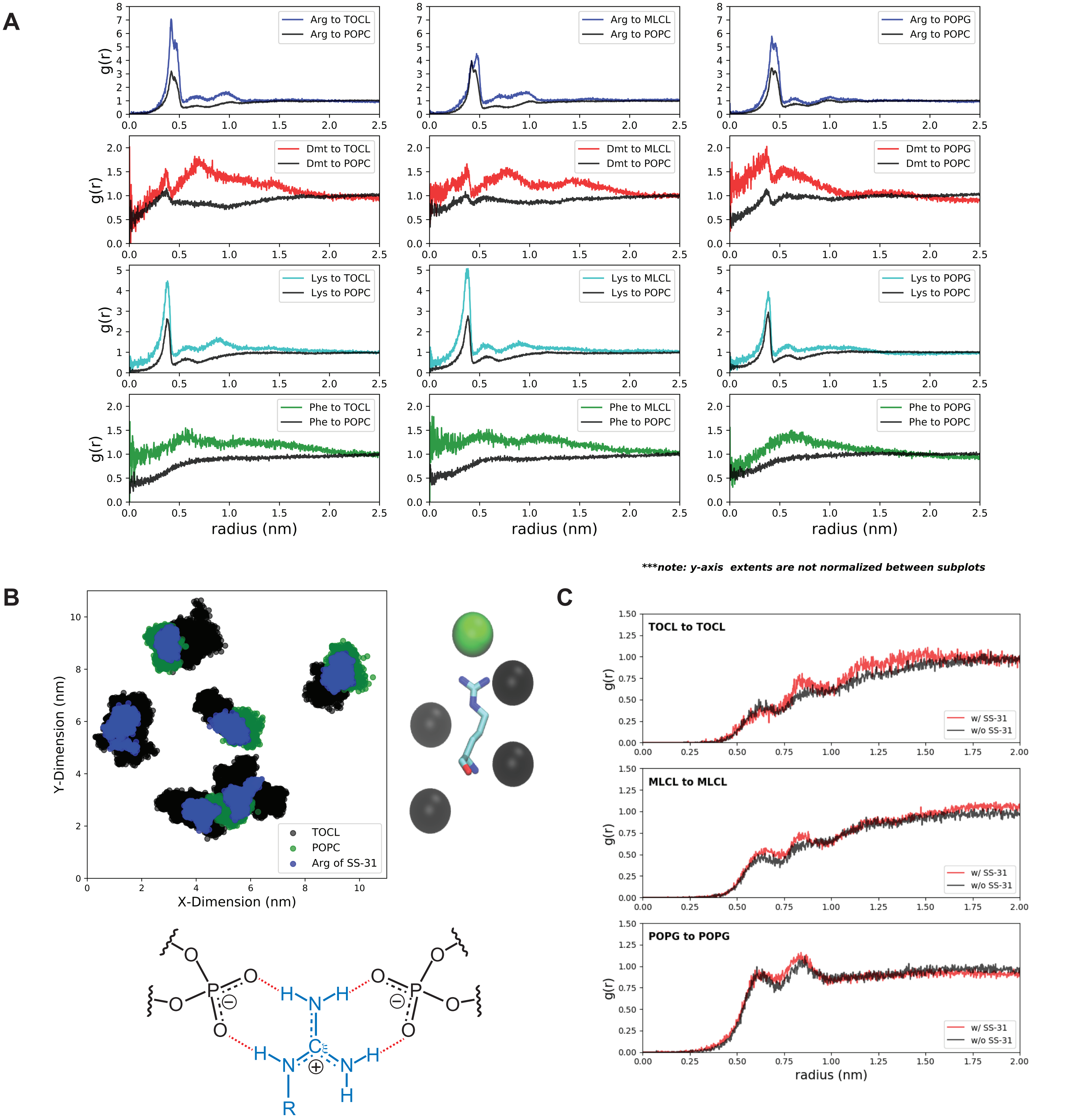

Figure S12. Lipid radial distribution profiles from MD simulations. *A*, Lipid distributions around SS-31 residues. Lateral (x-y plane) radial distribution profiles from side chain landmark atoms (the D-Arg Cζ atom; the 2′,6′-Dmt Oη atom; the Lys Nζ atom; or the Phe Cζ atom) to lipid headgroup phosphate atoms (TOCL and MLCL P_1, POPG and POPC, P). Note that plots have different y-axis scales to aid in distinguishing between curves. The landmark atoms on the aromatic residues (Dmt, Phe) can overlap with the phosphates by being positioned above or below the headgroups in the Z-direction, leading to elevated concentrations below the van der Waals radius. *B*, Coordination between SS-31 side chains and lipid phosphates. *Upper left,* top-down view of the lipid bilayer (in x-y space) showing the diffusion range of Arg (central Cζ atom, *blue*) over a 100 ns range within a typical simulation. POPC phosphates (*green*) and TOCL phosphates (*black*) that were proximal to the Arg over the time course are shown. *Upper right*, snapshot of typical orientation between Arg (*ball and stick representation*) and headgroup phosphates. *Lower*, schematic of bidentate Arg-phosphate complex stabilized by multiple hydrogen bonding interactions. *C*, Lipid-to-lipid concentration with and without SS-31 from MD simulations. Radial distribution profiles of phosphate to phosphate in each lipid headgroup (TOCL and MLCL P_1, POPG, P) in the upper leaflet of systems with SS-31 (*red*) and bilayers of systems without SS-31 (*black*).

A

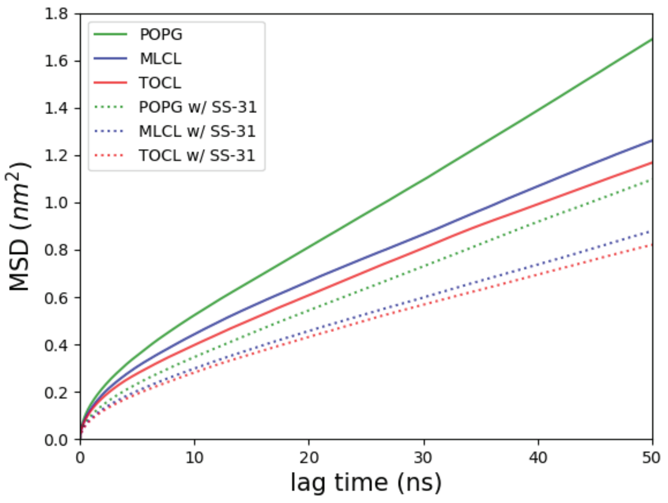

B

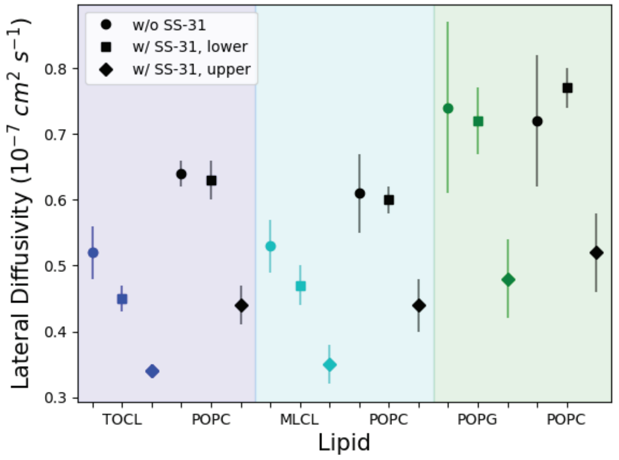

Lipid System	20:80 TOCL:POPC		20:80 MLCL:POPC		20:80 POPG:POPC	
	D_{xy} (10^{-7} cm^2 s^{-1})	95% Conf. Interval	D_{xy} (10^{-7} cm^2 s^{-1})	95% Conf. Interval	D_{xy} (10^{-7} cm^2 s^{-1})	95% Conf. Interval
Anionic Lipid w/ SS-31, upper	0.34	0.33 - 0.34	0.35	0.32 - 0.38	0.48	0.42 - 0.54
Anionic Lipid w/ SS-31, lower	0.45	0.43 - 0.47	0.47	0.44 - 0.51	0.72	0.67 - 0.77
Anionic Lipid	0.52	0.48 - 0.55	0.53	0.49 - 0.57	0.74	0.61 - 0.87
POPC w/ SS-31, upper	0.44	0.42 - 0.47	0.44	0.41 - 0.48	0.52	0.46 - 0.58
POPC w/ SS-31, lower	0.63	0.60 - 0.66	0.60	0.58 - 0.62	0.77	0.74 - 0.80
POPC	0.64	0.62 - 0.66	0.61	0.55 - 0.66	0.72	0.62 - 0.82

Figure S13. Mean square displacement of lipids with and without SS-31 from MD simulations. *A*, Representative mean square displacement analysis in the lateral (xy) plane of phosphates from headgroups from anionic lipids. *B*, Lateral (xy) lipid diffusion coefficients for bilayers with and without SS-31. Diffusion constants were calculated from mean square displacement analyses of each lipid's headgroup phosphate(s). The figure (*left*) and table (*right)* includes diffusion constants (D_{xy}) for lipid systems without any SS-31 at all, as well as separate diffusion constants for the upper (peptide-accessible) and lower (opposing) leaflets.

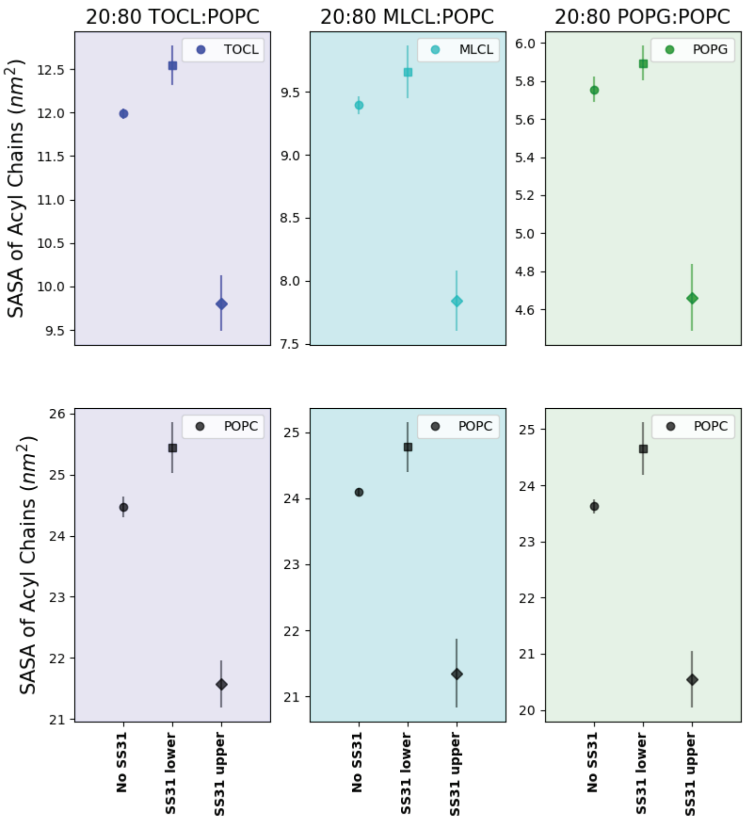

Lipid System	20:80 TOCL:POPC		20:80 MLCL:POPC		20:80 POPG:POPC	
	Acyl Chain SASA (nm^2)	95% Conf. Interval	Acyl Chain SASA (nm^2)	95% Conf. Interval	Acyl Chain SASA (nm^2)	95% Conf. Interval
Anionic Lipid w/ SS-31, upper	9.8	9.5 - 10.1	7.8	7.6 - 8.1	4.7	4.5 - 4.8
Anionic Lipid w/ SS-31, lower	12.5	12.3 - 12.8	9.7	9.4 - 9.9	5.9	5.8 - 6.0
Anionic Lipid	12.0	11.9 - 12.1	9.4	9.3 - 9.5	5.8	5.7 - 5.9
POPC w/ SS-31, upper	21.6	21.2 - 22.0	21.3	20.8 - 21.9	20.5	20.0 - 21.0
POPC w/ SS-31, lower	25.4	25.0 - 25.9	24.8	24.4 - 25.1	24.7	24.2 - 25.1
POPC	24.5	24.3 - 24.6	24.1	24.0 - 24.2	23.6	23.5 - 23.8

Figure S14. Acyl chain solvent accessible surface area from MD simulations. The average SASA of each lipid acyl chain region was calculated for all tested lipids with and without SS-31. Confidence intervals (95%) were calculated from the standard error of the mean from three equal regions of each trajectory. The figure (*left*) and table (*right*) include SASA measurements for lipid systems in the absence of SS-31 as well as separate SASA measurements for the upper (peptide-exposed) and lower (lacking peptide) leaflets of systems with SS-31. The presence of SS-31 in the upper leaflet decreased SASA of the acyl chain region for all tested lipids compared to both a) the lower leaflet of the same SS-31 containing system, and b) bilayer systems without SS-31. The presence of SS-31 in the upper leaflet also increased the SASA of the lower (opposing) leaflet compared with systems lacking SS-31 for specific lipids, including TOCL and POPC in the 20:80 TOCL:POPC system, POPC in the 20:80 MLCL:POPC system, and POPC in the 20:80 POPG:POPC system.

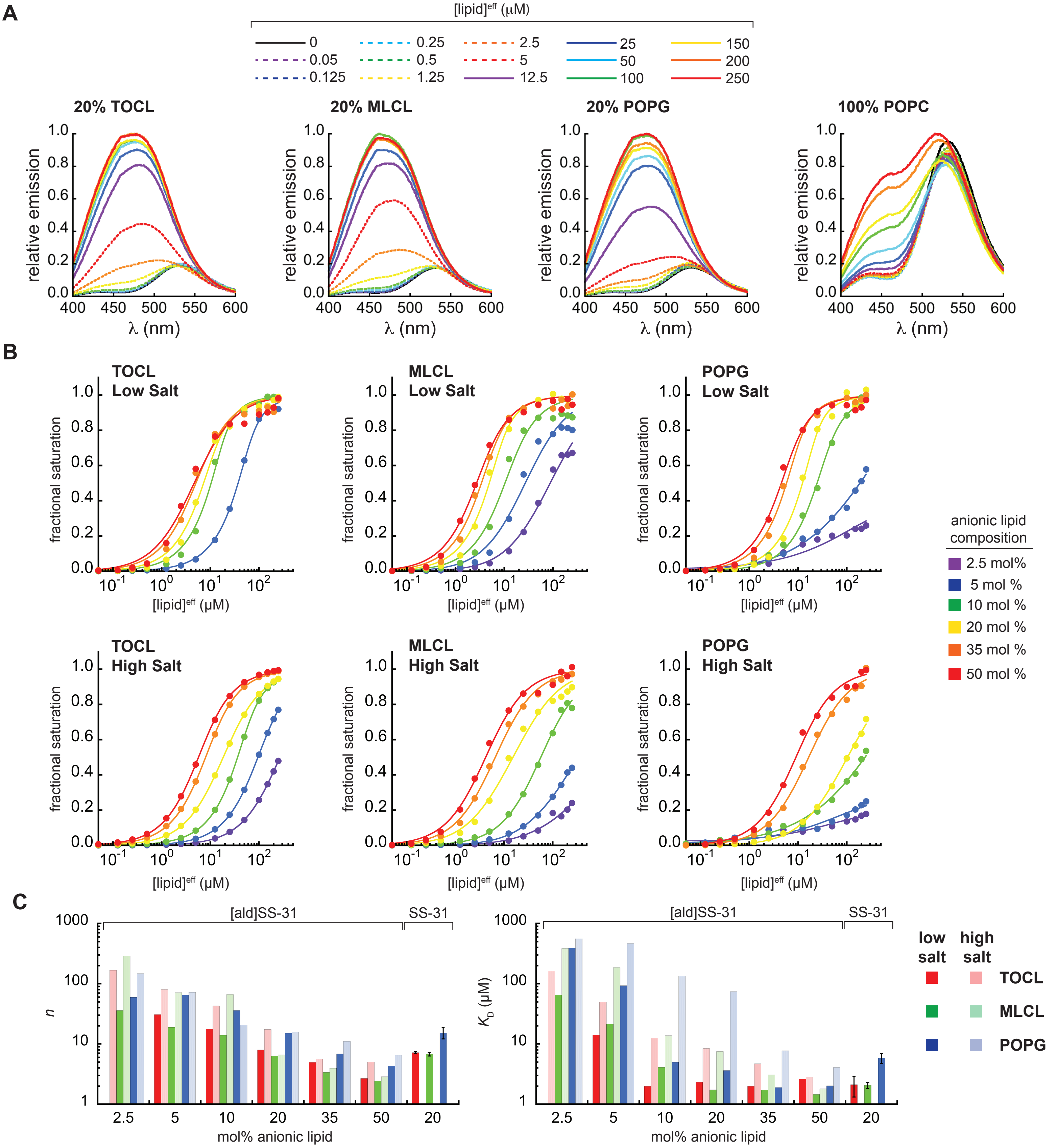

Figure S15. [ald]SS-31 binding isotherms with model membranes. *A*, [ald]SS-31 fluorescence scans. Emission spectra (λ_{ex} = 360 nm; λ_{em} = 400-600 nm) of 1 μM [ald]SS-31 in the presence of increasing concentrations of LUVs containing 20 mol% anionic lipid (TOCL, MLCL, or POPG) in a POPC background, or 100% POPC as indicated. Note that for each panel, emission intensities are shown as values scaled relative to the maximum emission for each particular lipid system to facilitate viewing of all spectra from 100% POPC samples. *B*, [ald]SS-31 binding isotherm composite. Binding curves for 1 μM [ald]SS-31 with liposomes containing anionic lipids (TOCL, MLCL, or POPG) at the concentrations indicated with a background of POPC. Measurements were made in the presence of low salt (*upper panels*) or high salt (*lower panels*). *C*, Equilibrium binding parameters. Values under "[ald]SS-31" show calculated parameters *n* and K_D from lipid titration isotherms shown in panel B, fit according to Eq. S3 for each sample (LUVs containing the indicated mol% of TOCL, MLCL, or POPG under high and low salt conditions). For comparison, values under "SS-31" show *n* and K_D values based on binding curves from endogenous SS-31 fluorescence (Figure 2B).

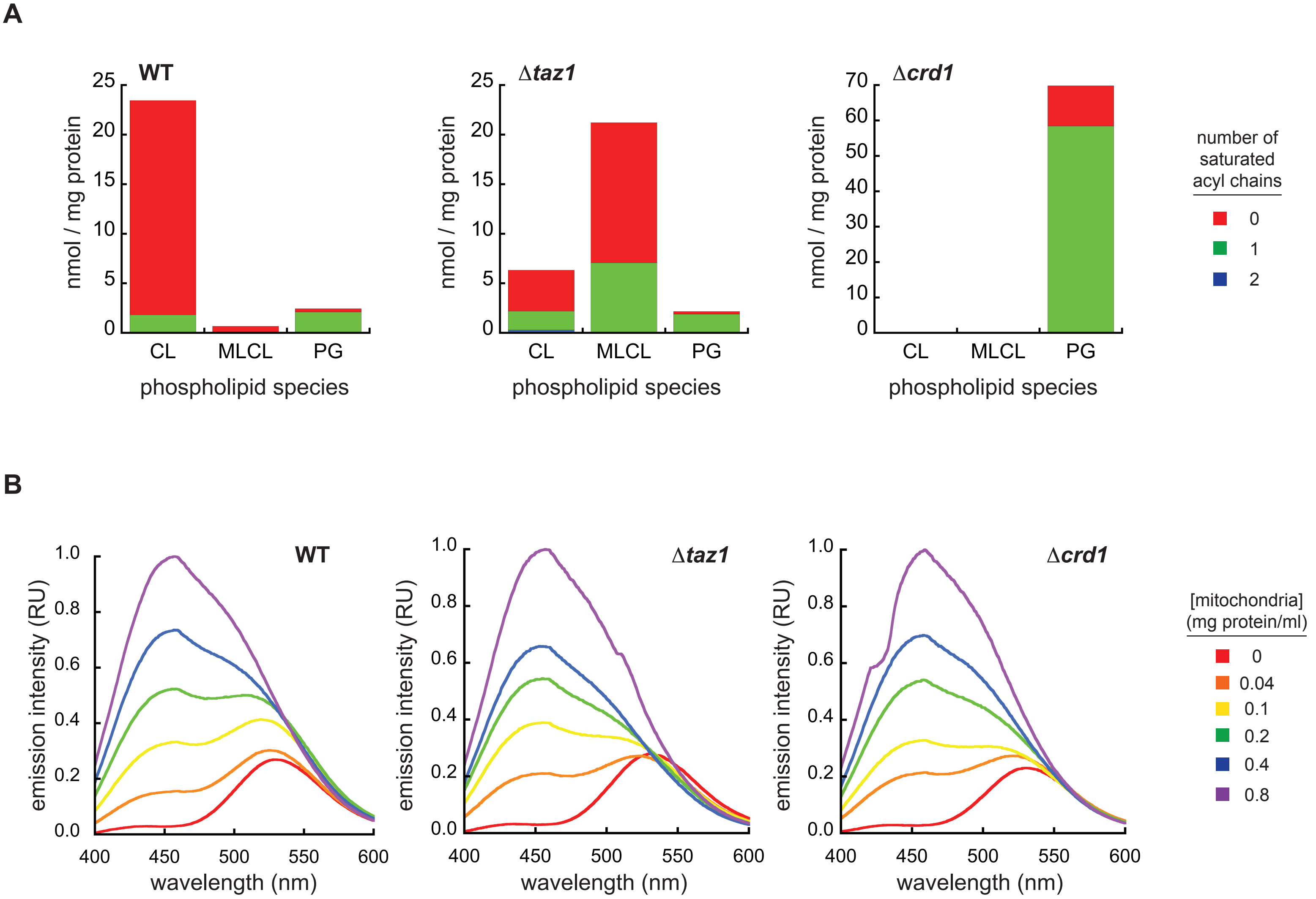

Figure S16. Interaction of SS-31 with isolated mitochondria. *A*, Acyl chain distribution of anionic phospholipids in mitochondria from different yeast strains. Based on lipidomics analysis of mitochondria from WT, Δ*taz1*, and Δ*crd1* yeast strains, anionic phospholipids CL, MLCL and PG were classified based on chain saturation as indicated (quantified as nmol lipid per mg mitochondrial protein). Shown are the means of n=3 individual samples per strain. *B*, Representative background-subtracted and normalized emission spectra containing 1 μM [ald]SS-31 with the indicated concentration of isolated mitochondria.

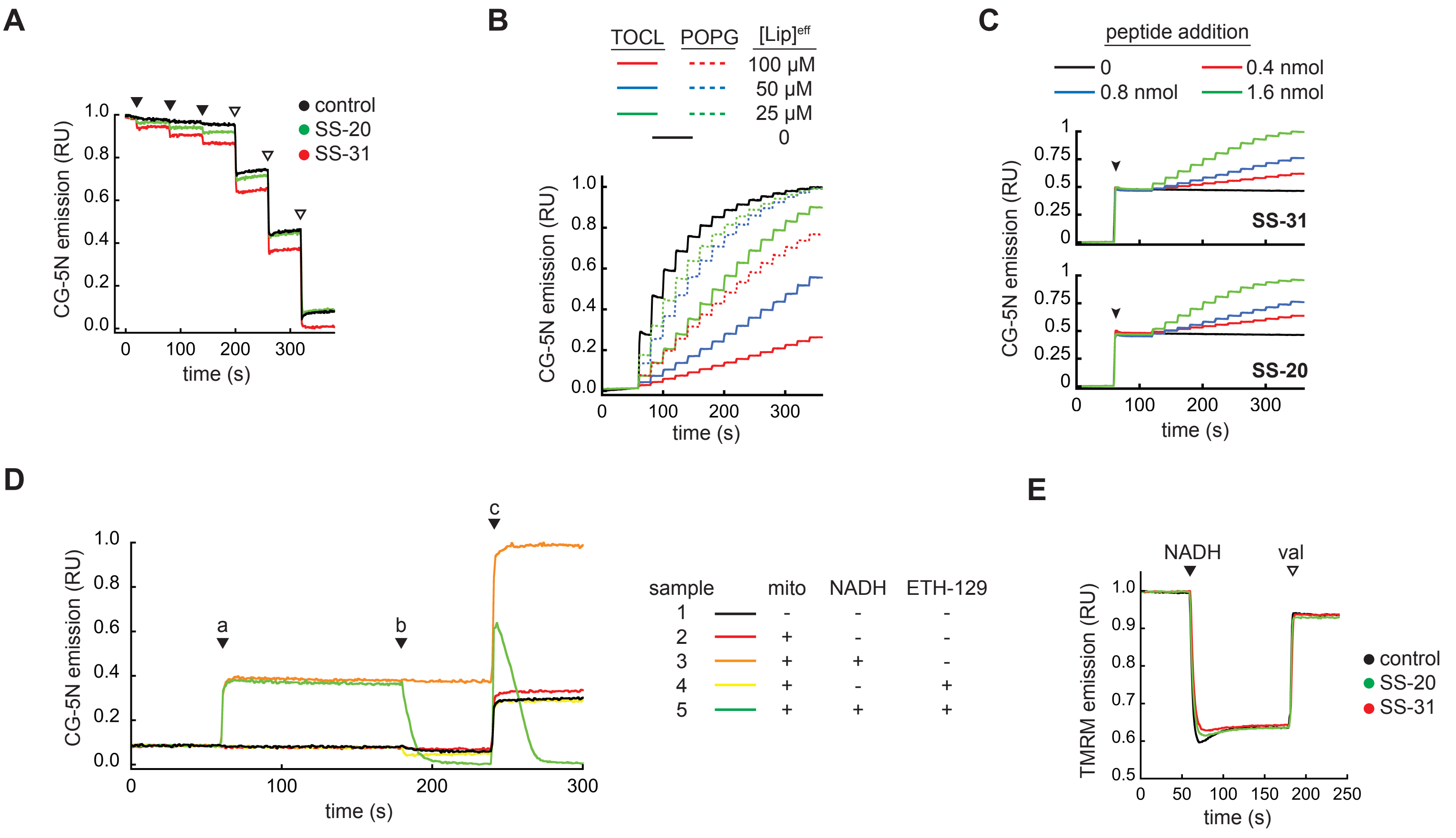

Figure S17. Fluorescence-based assays of calcium dynamics and $\Delta\Psi_m$. *A*, Effect of SS peptides on CG-5N fluorescence under conditions used to measure model membranes. Samples containing minimal LUV buffer (20 mM HEPES, pH 7.5) in the presence of 1 μM CG-5N and supplemented with 2.5 μM $CaCl_2$ were tested for the effects of SS peptide on probe emission. Time course measurements show three additions of peptide (2.5 nmol each addition) or vehicle only (*black arrowheads*) followed by three additions of EDTA, pH 7.5 (1 nmol each addition, *open arrowheads*). *B*, Effect of anionic bilayers on the CG-5N response to added Ca^{2+}. Time course measurements of CG-5N emission in the absence or presence of LUVs (20 mol% TOCL or POPG in a host POPC background) at the concentrations indicated. Starting at t=60 s, $CaCl_2$ was titrated in increments of 1 nmol at 20 s intervals. *C*, Effect of SS peptides on interfacial Ca^{2+} binding. Time course measurements of CG-5N emission in solutions containing LUVs (20 mol% TOCL, $[Lip]^{eff}$ = 100 μM) subjected to 5 nmol $CaCl_2$ addition (arrowhead, t=60s) followed by addition of SS-20 or SS-31 at the indicated amounts at 20 s intervals. *D*, Calcium concentration time courses. Time course measurements of CG-5N emission with samples containing mitochondria measurement buffer (20 mM HEPES, 300 mM sucrose, 2 mM potassium phosphate, 0.05% BSA, pH 7.5) containing 1 μM CG-5N with or without mitochondria (50 μg ml^{-1}) as indicated. At the indicated time points (*black arrowheads*), the following additions were made: a) 1 mM NADH or buffer only; b) 5 μM ETH-129 or DMSO only; c) 25 μM $CaCl_2$. Emission data were normalized relative to values just before addition at point a. *E*, Membrane potential time courses. Time course measurements of TMRM emission with samples identical to those in panel B, but containing 50 nM TMRM, with mitochondria in the absence of peptide (control) or preincubated with 25 μM SS-31 or SS-20 as indicated. NADH (1 mM) and valinomycin (1 μM) were added at time points indicated by *black* and *open arrowheads,* respectively. Emission data were normalized relative to values just prior to NADH addition.

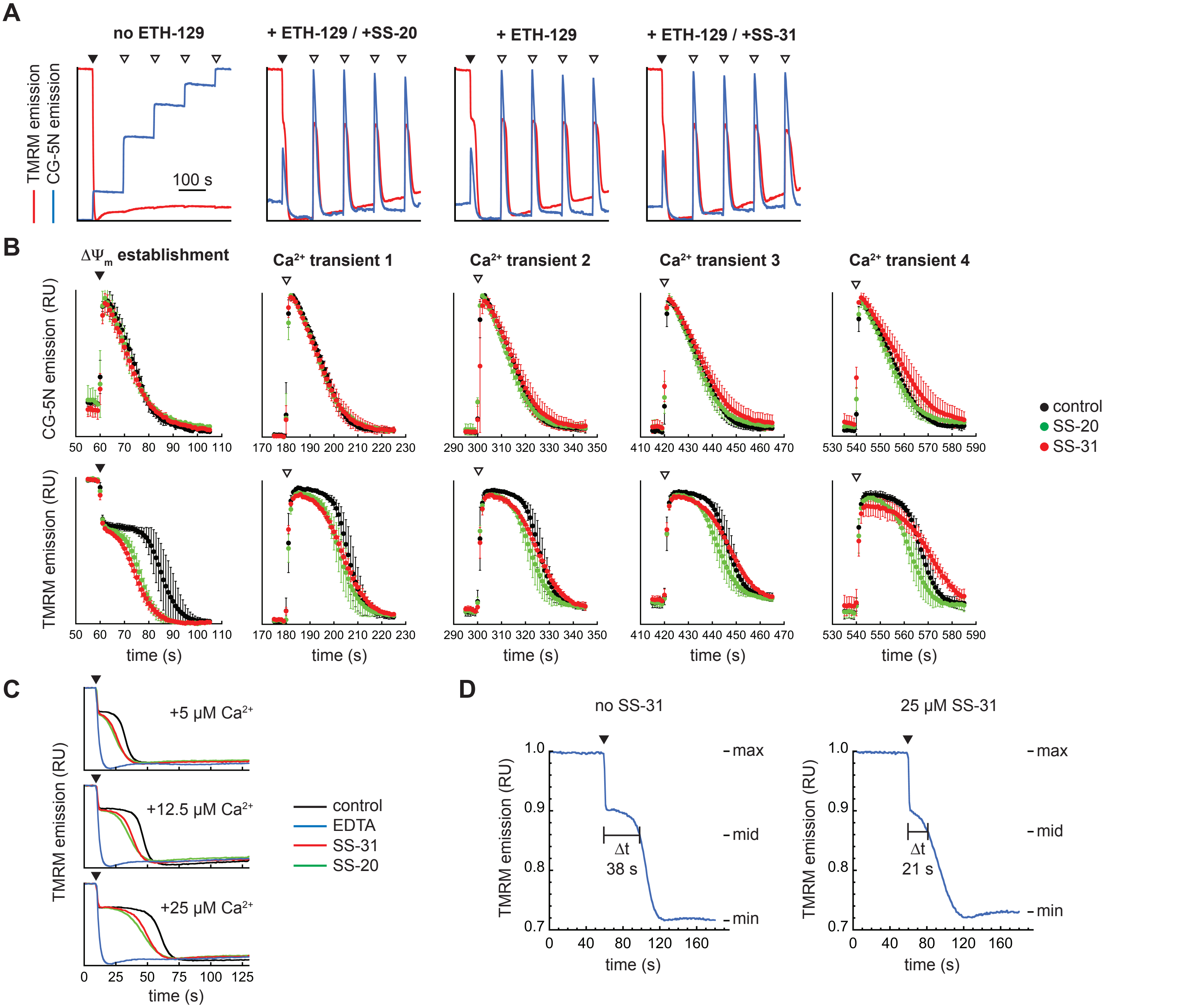

Figure S18. Kinetic responses of $\Delta\Psi_m$ and external [Ca^{2+}]. *A*, Effect of SS peptides on mitochondrial Ca^{2+} uptake. Time courses of TMRM ($\Delta\Psi_m$) or CG-5N (external [Ca^{2+}]) emission were made in solutions containing mitochondria (50 µg ml^{-1}) pre-treated with or without 5 µM ETH-129, 25 µM SS-20 and/or 25 µM SS-31 as indicated. Time courses included $\Delta\Psi_m$ generation following addition of 1 mM NADH (*black arrowheads*) and calcium transients following addition of 25 µM $CaCl_2$ (*open arrowheads*). To facilitate comparisons, both TMRM and CG-5N emission data were normalized relative to the full range of the probe response over the course of each measurement. Note that the "+ETH-129" and the "+ETH-129 / + SS-31" panels are identical to those in Figure 7C, shown here to enable direct comparison. *B*, Expanded views of $\Delta\Psi_m$ establishment and Ca^{2+} transients are shown for CG-5N measurements (*upper panels*) and TMRM measurements (*lower panels*). Data represent individual traces (n=3 ± SD) from Figure S18A from samples in the presence of ETH-129 and in the absence of SS peptide (control), or in the presence of 25 µM SS-20 or SS-31 as indicated. *C*, TMRM traces of $\Delta\Psi_m$ generation following addition of 1 mM NADH (*black arrowheads*) in samples containing the indicated [$CaCl_2$] and supplemented with vehicle only (control), 25 µM EDTA, 25 µM SS-20, or 25 µM SS-31 as indicated. *D*, Quantitative analysis of $\Delta\Psi_m$ establishment for the dose response curve shown in Figure 7D. TMRM traces of mitochondria in the presence of 25 µM $CaCl_2$ and ETH-129 were measured over time courses in which respiratory substrate (1 mM NADH) was added at t=60 s (*black arrowhead*) and the $\Delta\Psi_m$ was established over an additional 120 s. From the kinetic traces of TMRM emission, three points were measured: max (average of points prior to NADH addition), min (average of last 20 s of traces) and mid (average of max and min, representing the midpoint of the TMRM emission range). As a quantitative measure of the Ca^{2+}-dependent temporal delay in $\Delta\Psi_m$ establishment, we calculated Δt as the time required to reach the mid TMRM value following respiratory substrate addition.

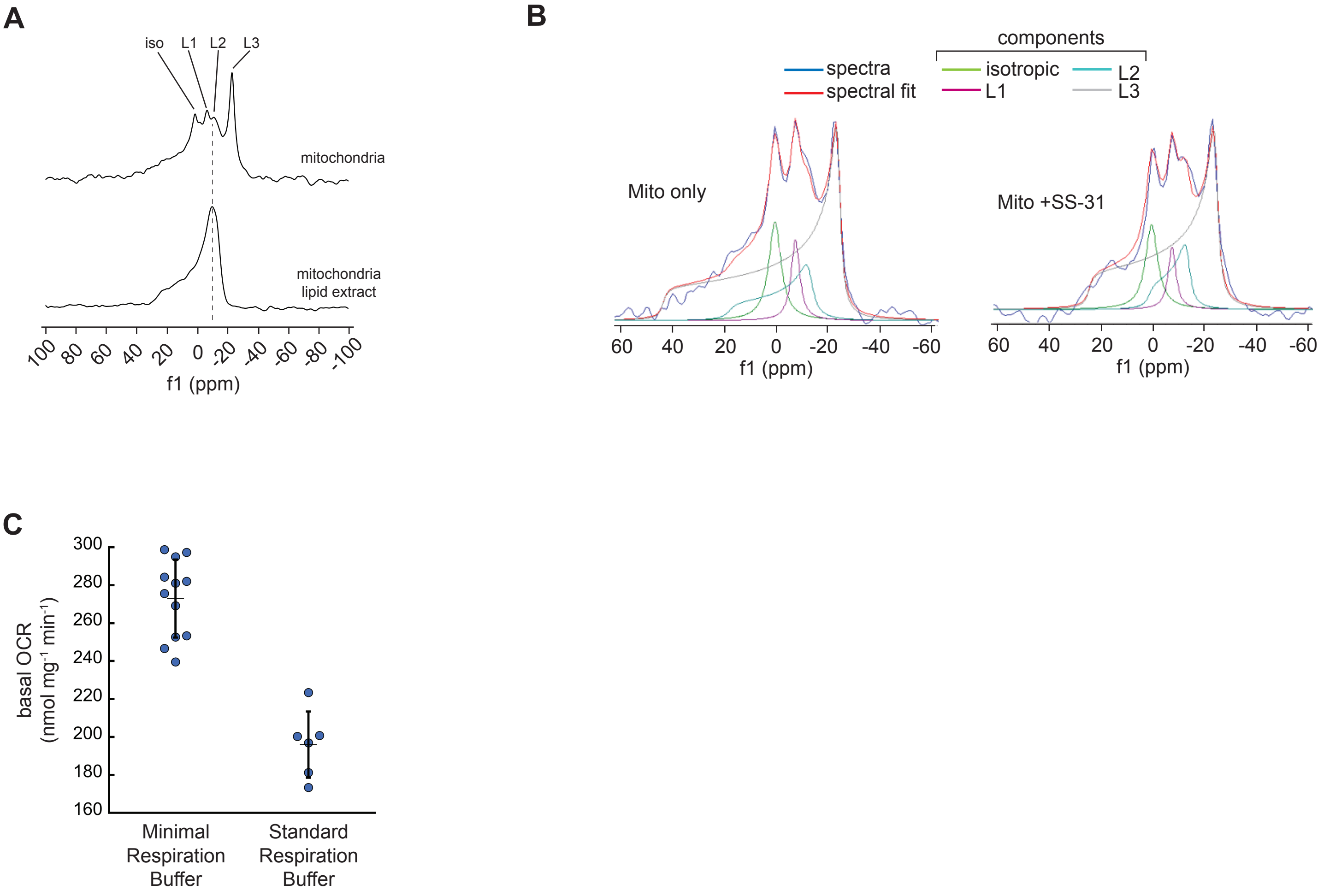

Figure S19. NMR and respirometry measurements with calcium stress. *A* and *B*, ^{31}P ssNMR measurements. *A*, Static wide-line ^{31}P NMR spectra of isolated mitochondria (*upper panel*) and MLVs prepared from extracted mitochondrial lipids (*lower panel*). *B*, Spectra of isolated mitochondria in the absence (*left*) or presence (*right*) of SS-31. Based on spectral deconvolution (dmFit), identified peaks from mitochondria samples include an isotropic component at 0 ppm ("Iso") and three high-field signals labeled "L1", "L2" and "L3" centered at -7, -12, and -23 ppm, respectively. *C*, Condition-dependent respirometry measurements. Basal oxygen consumption rates are shown for measurement under Minimal Respiration Buffer (data from Figure 7G) and Standard Respiration Buffer as indicated. Individual points are shown with means ± SD.
